# Synthesis, Structure, Carbohydrate Enzyme Inhibition, Antioxidant Activity, In Silico Drug-Receptor Interactions and Drug-Like Profiling of the 5-Styryl-2-Aminochalcone Hybrids

**DOI:** 10.3390/molecules26092692

**Published:** 2021-05-04

**Authors:** Malose J. Mphahlele, Emmanuel Ndubuisi Agbo, Yee Siew Choong

**Affiliations:** 1 Department of Chemistry, College of Science, Engineering and Technology, University of South Africa, Private Bag X06, Florida 1710, South Africa; piruesbest@yahoo.com; 2 Institute for Research in Molecular Medicine (INFORMM), Universiti Sains Malaysia, Penang 11800, Malaysia

**Keywords:** styryl-aminochalcones, intramolecular hydrogen bonding, α-glucosidase, α-amylase, antioxidant, drug-receptor interaction, cytotoxicity

## Abstract

The 2-amino-5-(3/4-fluorostyryl)acetophenones were prepared and reacted with benzaldehyde derivatives to afford the corresponding 5-styryl-2-aminochalcone hybrids. The *trans* geometry of the styryl and α,β-unsaturated carbonyl arms, and the presence of NH^…^O intramolecular hydrogen bond were validated using ^1^H-NMR and X-ray data. The 2-amino-5-styrylacetophenones and their 5-styryl-2-aminochalcone derivatives were screened in vitro for their capability to inhibit α-glucosidase and/or α-amylase activities. Their antioxidant properties were evaluated in vitro through the 2,2-diphenyl-1-picrylhydrazyl (DPPH) and nitric oxide (NO) free radical scavenging assays. Kinetic studies of the most active derivatives from each series against α-glucosidase and/or α-amylase activities have been performed supported by molecular docking studies to determine plausible protein–ligand interactions on a molecular level. The key aspects of the pharmacokinetics of these compounds, i.e., absorption, distribution, metabolism, and excretion have also been simulated at theoretical level. The most active compounds from each series, namely, **2a** and **3e**, were evaluated for cytotoxicity against the normal monkey kidney cells (Vero cells) and the adenocarcinomic human epithelial (A549) cell line to establish their safety profile at least in vitro.

## 1. Introduction

Non-insulin-dependent diabetes mellitus is a serious global health concern and according to the World Health Organization’s (WHO’s) report of 2018, about 90% patients suffering from diabetes have been diagnosed for type 2 diabetes mellitus (T2DM) [1]. This metabolic disorder is characterized by an increased level of glucose in the blood known as post-prandial hyperglycemia (PPHG), which causes vascular complications leading to damage of vital organs [2,3]. The pancreas becomes unable to produce enough insulin or fail to compensate for insulin resistance due to glucotoxicity and lipotoxicity, resulting in T2DM [4]. α-Amylase is secreted from the pancreas, and this enzyme is responsible for converting large starch and glycogen molecules into simpler absorbable sugars [1,5] that are, in turn, converted by α-glucosidase into glucose for intestinal absorption [6,7]. Dual inhibition of the activity of intestinal α-glucosidase and pancreatic α-amylase suppresses carbohydrate digestion, in turn, delay glucose uptake leading to reduced blood sugar levels [1,8,9]. Glucosidase inhibitors continue to attract considerable interest in medicinal chemistry due to their promising therapeutic potential in the treatment of disorders such as diabetes, human immunodeficiency virus (HIV) infection, metastatic cancer, and lysosomal storage diseases [10]. Oxidative stress is one of the most common factors underlying insulin resistance and diabetes [11,12,13,14]. Oxidative stress may result from the interaction of scavenger receptors, such as RAGE, with advanced glycoxidation end-products (AGEs) formed from the non-enzymatic glycation of proteins, lipids and nucleic acids with reducing sugars and with the products of glucose metabolism and their oxidation products, a phenomenon which is fostered by chronic hyperglycemia [15]. It is envisaged that compounds that can delay, inhibit, or prevent the oxidative damage by scavenging free radicals will help to ameliorate complications such as coronary heart disease or stroke, and non-vascular pathologies such as cancer [16].

**Figure 1:**
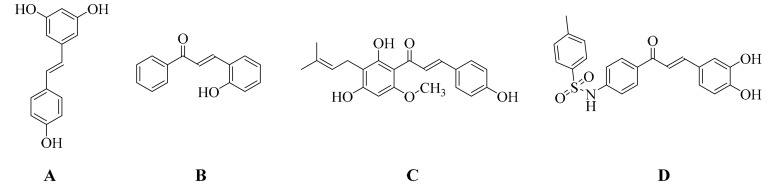
Structures of resveratrol (**A**), hydroxychalcones (**B**,**C**) and 4-(*p*-toluenesulfonamide)chalcone (**D**) with antihyperglycemic properties.

Nature-based small molecular weight ligands such as stilbenes (1,3-diphenylpropenes) and chalcones (1,3-diphenylprop-2-ene-1-ones) are not only important for the inhibition of cancer, but are also useful for the treatment of diabetes, cardiovascular and neurological diseases as well as other chronic diseases [17,18]. Resveratrol (*trans*-3,4,5-trihydroxystilbene) **A** (Figure 1), for example, has been found to be capable of delaying the absorption of carbohydrates and lower postprandial glucose concentrations in high-fat diet-induced hyperglycemic mice [19]. Substituted chalcones either from natural or synthetic sources, on the other hand, are well known insulin sensitizers, which modulate diverse antidiabetic targets [20]. Several studies verified the effectiveness of chalcone-based compounds as antihyperglycemic and/or hypoglycemic agents through in vitro and in vivo experimental responses [21]. A series of hydroxychalcones (**B** and **C**), 4-aminochalcones and the 4-(*p*-toluenesulfonamide)chalcone (**D**) derivatives have been evaluated for inhibitory effect in vitro against α-glucosidase, α-amylase, and β-amylase activities [22]. Among these classes of α,β-unsaturated carbonyl compounds, only the 4-aminochalcones and their *N*-tosyl derivatives were found to exhibit significant and increased inhibitory activities against these carbohydrate hydrolyzing enzymes, respectively. An in vivo study carried out with alloxan induced diabetic Wister male albino rats (100 mg/kg) revealed the 4-aminochalcones to exhibit significant antidiabetic efficacy with decreased blood glucose levels in the diabetic rats compared with control rats [23]. Molecular docking (in silico) study of the 4-aminochalcones into α-glucosidase binding sites revealed π–π stacked, π-cationic, polar, electrostatic and hydrophobic bonding interactions with key residues in the binding pockets of this enzyme [24]. The relatively planar conformation in the case of flavonoid derivatives such as quercetin, for example, is considered to allow for increased conjugative effect resulting in stronger α-glucosidase inhibitory and antioxidant activity than the non-planar analogues [25]. Computational studies also showed that electron donating groups on an aromatic ring increase the electron density at the *ortho* and *para* positions of the π-system resulting in increased antioxidant activity while electron withdrawing groups decrease the activity [24]. Electron donating groups in the case of stilbene derivatives facilitate the addition of oxygen radicals to the *ortho* ring position [26].

Recently the concept of hybrid antihyperglycemic agents has attracted much attention for treating complications associated with T2DM [27]. Molecular hybrids often act on multiple therapeutic targets because of the presence of two or more different, covalently fused pharmacophores. Despite the wide distribution of stilbenes and chalcones in plant species, molecular hybrids merging these two pharmacophores seem not to have occurred in nature. One of the goals of medicinal chemistry research and drug discovery is to design and develop compounds that both show desired biological activities and are easily accessible. The glucose lowering potentials of stilbene derivatives and aminochalcones inspired us to link these two scaffolds based on 2-amino-5-iodoacetophenone as a template for initial olefination at C-5 with phenylboronic acid derivatives followed by Claisen–Schmidt aldol condensation of the intermediate 2-amino-5-styrylacetophenones with 3/4-fluorobenzaldehyde. This rational design was inspired by the literature precedents that the electron-withdrawing inductive effect of fluorine atom could help the drug molecules in forming hydrogen and/or halogen bonding interactions with the protein targets, and thus enhance biological activity [28]. Moreover, we envisaged that the presence of a fluorophenyl ring on the conjugated scaffold would result in increased π-electron distribution to lead to increased noncovalent or hydrophobic (e.g., π–π stacking, π–π T-shaped, π–alkyl, alkyl) interactions of compounds with the receptors. In our view, increased conjugative effects due to the presence styryl and potential hydrogen bonding 2-aminochalcone components as well as the presence of various substituents on the phenyl groups could increase their inhibitory effect against α-glucosidase and/or α-amylase activities. Since an ideal anti-diabetic drug should exhibit hypoglycemic properties and inhibit oxidative stress, we also evaluated the test compounds for antioxidant potential through the 2,2-diphenyl-1-picrylhydrazyl (DPPH) and nitric oxide (NO) free radical scavenging assays. Kinetic assays (in vitro) and molecular docking (in silico) were performed on the most active compounds from each series against these carbohydrate hydrolyzing enzymes to determine plausible protein-drug interactions on a molecular level. Moreover, the ADME (absorption, distribution, metabolism, and excretion) properties of the most active derivatives were also predicted using in silico methods.

## 2. Results and Discussion

### 2.1. Chemical Synthesis and Structural Analysis

**Scheme 1:**
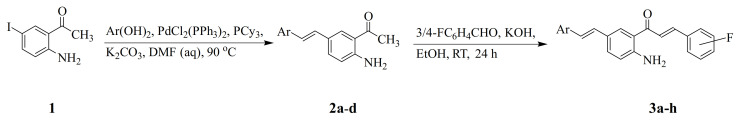
Synthesis of the 5-styryl-2-aminochalcone hybrids **3a**–**h**.

**Figure 2:**
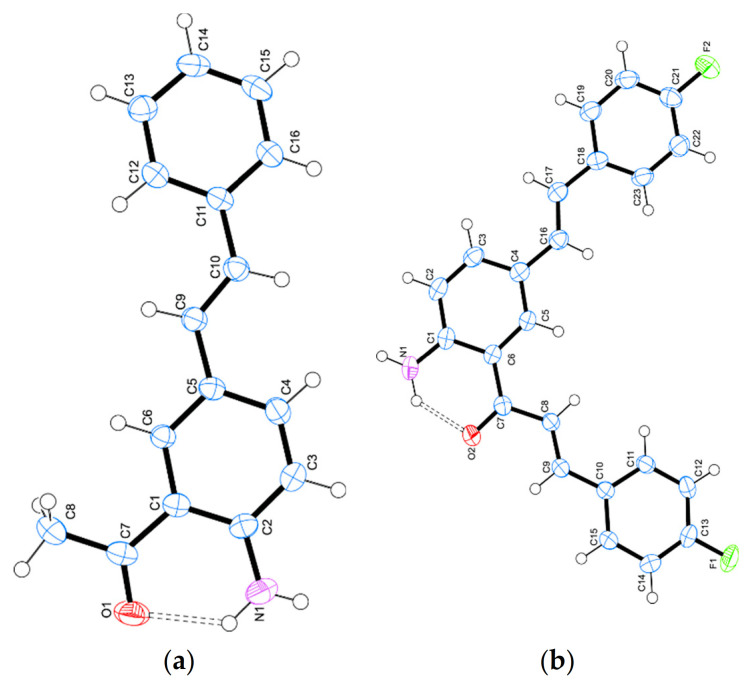
Molecular structure of compound **2a** (**a**) and **3f** (**b**) determined from single-crystal X-ray data, showing the atom-labelling scheme, which differs from systematic numbering.

The synthesis of the title compounds was achieved as outlined in Scheme 1 below via initial Suzuki–Miyaura cross-coupling of 2-amino-5-iodoacetophenone (**1**) with phenylboronic acid derivatives as coupling partners to afford the corresponding 2-amino-5-styrylacetophenones **2a**–**d** (Table 1 shows the designation of substituents). These compounds and their derivatives were characterized using a combination of NMR, FT-IR and mass spectroscopic techniques complemented with single crystal X-ray diffraction method. Copies of the NMR (^1^H- and ^13^C-) and FT-IR spectra have been included as Figures S1 and S2 of the Supplementary Information (SI), respectively. The ^1^H NMR spectra of **2a**–**d** revealed a set of doublets in the aromatic region for the olefinic protons with vicinal coupling constant (*J*) values in the range 15.0–16.5 ppm, which confirmed the *trans* geometry of the styryl wing. The protons of the amino group resonate as an intense singlet in the aromatic region around δ 6.41 ppm. The *trans* geometry of the styryl arm of compounds **2a**–**d** was distinctly confirmed by XRD structure of compound **2a** (CCDC 2053923) as a representative model (Figure 2a). XRD method also revealed the presence of an intramolecular hydrogen bond between hydrogen atom of the amino group and carbonyl oxygen with N(1)-H(1A)^...^O(1) bond distance of 1.98(3) Å and bond angle of 130(2)°. Further transformation of compounds **2a**–**d** via Claisen-Schmidt aldol condensation with 3-fluorobenzaldehyde or 4-fluorobenzaldehyde under basic conditions afforded the 5-styryl-2-aminochalcone derivatives **3a**–**d** or **3e**–**h**, respectively. The ^1^H- and ^13^C NMR spectra of compounds **3** (refer to Figure S1 in SI) revealed the presence of increased number of signals in the aromatic region compared to the corresponding substrates **2**. A singlet for NH_2_ of compounds **3** still resonated in the aromatic region around δ = 7.61 ppm. The presence of a singlet in the ^13^C-NMR spectra of these compounds around δ = 191.0 ppm for the carbonyl carbon further confirmed their α,β-unsaturated carbonyl nature. The olefinic protons of the styryl and chalcone wings resonate as two sets of doublets among the aromatic proton signals with vicinal coupling constant (*J*) values of 15.0–16.5 ppm consistent with their *trans* geometry. The *trans* geometry of the styryl and chalcone arms of compounds **3a**–**h** were distinctly confirmed by the XRD structure of compound **3f** (CCDC 2035809) as a representative model within these series (Figure 2b). XRD method revealed the existence of a six-membered intramolecular hydrogen bonded ring (graph set descriptor, S(6)) involving hydrogen atom of the amino group and carbonyl oxygen with N(1)-H(1A)^...^O(2) bond distance of 1.88(2) Å, which helps to stabilize the planarity of these molecular constructs. Integrating intramolecular hydrogen bonding formation in drug design has become an exciting challenge in medicinal or bioorganic chemistry especially in the context of drug-receptor interactions. Intramolecularly hydrogen bonded drug-like molecules have been found to exhibit favorable alignment with the protein pocket resulting in increased ligand-receptor interactions [29]. Moreover, conformational restriction of small drug molecules due to intramolecular hydrogen bonds results in increased lipophilicity, membrane permeability and pharmacological activity.

Compounds **3a**–**h** and their corresponding precursors **2a**–**d** were, in turn, evaluated for inhibitory properties against α-glucosidase and α-amylase, and also for DPPH and NO free radical scavenging potential.

### 2.2. Biological Evaluation

The test compounds were evaluated for inhibitory activity in vitro against yeast α-glucosidase from *Saccharomyces cervisiae* using acarbose as a reference standard. Acarbose is a competitive α-glucosidase inhibitor [30] which has been found to delay the absorption of carbohydrate from the small intestine and to reduce postprandial hyperglycemia in patients with T2DM than metformin and sulfonylureas [31]. The half maximal inhibitory concentration (IC_50_) values were calculated from the dose-dependent curves as the concentrations of inhibitors required to decrease 50% of the enzyme activity (Table 2). Only compound **2a** within the series **2a**–**d** was found to exhibit significant inhibitory effect against α-glucosidase compared to acarbose (IC_50_ = 0.95 ± 0.28 µM) with an IC_50_ value of 5.4 ± 0.10 µM. The trend in activity of these compounds decreases with the increasing size of the substituent at the *para* position of the styryl arm as follows: C_6_H_5_- (**2a**) > 4-FC_6_H_4_- (**2b**) > 4-ClC_6_H_4_- (**2c**) > 4-CH_3_OC_6_H_4_- (**2d**). A combination of the 5′-styryl group and 2′-amino-3-fluorochalcone scaffold resulted in significantly reduced inhibitory effect for **3a** (IC_50_ = 17.8 ± 0.32 µM). An improved inhibitory activity against α-glucosidase, on the other hand, was observed for **3b** with a combination of 5′-(4-fluorostyryl) group and 2′-amino-3-fluorochalcone scaffold, and its IC_50_ value is 6.1 ± 0.24 µM. The analogous 5′-(4-chlorostyryl) substituted derivative **3c** exhibited slightly improved activity (IC_50_ = 12.6 ± 0.31 µM) compared to the corresponding substrate **2c**. A combination of strongly lipophilic 5′-(4-methoxystyryl) group and the 2′-amino-3-fluorochalcone moiety on the framework of **3d** resulted in improved activity against α-glucosidase (IC_50_ = 9.4 ± 0.50 µM). Compound **3e** comprises the 5′-styryl and 2′-amino-4-fluorochalcone moieties and this derivative exhibited the highest activity amongst the 5-styryl-2-aminochalcone hybrids with an IC_50_ value of 5.1 ± 0.61 µM. Its activity is comparable to that of the corresponding substrate **2a**. A combination of the 5′-(4-fluorostyryl) and 2′-amino-4-fluorochalcone moieties resulted in significant inhibitory activity for **3f** (IC_50_ = 6.9 ± 0.37 µM). The presence of the electron withdrawing 5′-(4-chlorostyryl) group on the 2′-amino-4-fluorochalcone scaffold, on the other hand, resulted in significantly reduced activity for **3g** (IC_50_ = 19.2 ± 0.47 µM). An improved activity against α-glucosidase was also observed for hybrid **3h** substituted with a strongly π-electron delocalizing 4-methoxystyryl group with an IC_50_ value of 10.5 ± 0.18 µM. However, this compound is slightly less active than the isomeric **3d**.

α-Amylase assay of compounds **2** and **3** was carried out following protocol enclosed in α-amylase inhibitor screening kit using acarbose (IC_50_ = 1.03 ± 0.05 µM) and specific α-amylase inhibitor from *Triticum aestivum* (IC_50_ = 0.31 ± 0.05 µM) as reference standards for the assay. 2-Amino-5-styrylacetophenone **2a** was found to be the most active within the category **2a**–**d** and to exhibit significant inhibitory activity against α-amylase compared to the reference standards with an IC_50_ value of 2.3 ± 0.20 µM. Significantly reduced inhibitory effect against α-amylase activity was, however, observed for **2b** substituted with an electron withdrawing fluorine atom at the *para* position of the phenyl group with an IC_50_ value of 19.3 ± 0.42 µM. The 4-chlorostyryl derivative **2c**, on the other hand, exhibited significant activity than **2b** with an IC_50_ value of 5.8 ± 0.51 µM. The presence of a bulky 4-methoxystyryl arm on the scaffold of **2d** resulted in moderate inhibitory effect against α-amylase with an IC_50_ value of 9.0 ± 0.31 µM. The 5-styryl-2-aminochalcones **3a**–**h** exhibited different trend in activity depending on the position of fluorine on the B-ring of the chalcone framework. A combination of the 5′-styryl group and 2′-amino-3-fluorochalcone scaffold resulted in significantly reduced activity for the styryl-chalcone hybrid **3a** with an IC_50_ value of 10.7 ± 0.21 µM. The isomeric 2′-amino-5′-styryl-4-fluorochalcone **3e**, on the other hand, exhibited significant inhibitory effect against α-amylase with an IC_50_ value of 1.6 ± 0.52 µM. A similar trend in activity was observed for the 2′-amino-5′-(4-fluorostyryl)-3-fluorochalcone **3b** and the isomeric 2′-amino-5′-(4-fluorostyryl)-4-fluorochalcone **3f** with IC_50_ values of 15.6 ± 0.60 µM and 9.5 ± 0.41 µM, respectively. 2′-Amino-5′-(4-chlorostyryl)-3-fluorochalcone **3c** and its isomer 2′-amino-5′-(4-chlorostyryl)-4-fluorochalcone **3g**, on the other hand, exhibited improved activity against this enzyme with IC_50_ values of 2.5 µM and 1.7 ± 0.25 µM, respectively. Both the 2′-amino-5′-(4-methoxystyryl)-3-fluorochalcone **3d** and the isomer **3h** were found to be moderately active against α-amylase with IC_50_ values of 7.0 ± 0.45 µM and 7.6 ± 0.20 µM, respectively.

Hitherto, the 2-aminochalcones with different substitution pattern on the B-ring [32] and the 4-aminochalcones and the 4-(sulfonamido)-substituted chalcones [33] exhibited no antioxidant activities by the DPPH radical scavenging method. Reduced or lack of antioxidant activity of these compounds was attributed to the presence of intramolecular N-H···O hydrogen bond between amino and kenone group of the 2-aminochalcones and their sulfanamido derivatives which is envisaged to deactivate the unsaturated moiety [32,34]. The antioxidant properties of (*E*)-stilbenes, on the other hand, has been found to be dependent on the electron-donating or electron-withdrawing nature of the functional groups at the 4 and 4′ positions, respectively [35]. A combination of the styryl and the electrophilic α,β-unsaturated carbonyl arms on the same molecular framework encouraged us to evaluate the test compounds for antioxidant properties through the DPPH and NO radical scavenging assays. Moderate free radical scavenging activity was observed for the 2-amino-5-styrylacetophenones **2a**–**d** against ascorbic acid (IC_50_ = 4.2 ± 0.27 µM) with the IC_50_ values in the range of 7.1 ± 0.32 µM to 15.8 ± 0.21 µM in the DPPH assay and IC_50_ values of 7.2 ± 0.40 µM to 25.9 ± 0.61 µM in the NO assay. Compound **2a** has potential to inhibit both α-glucosidase and α-amylase activities, and to reduce oxidative stress. Significantly reduced antioxidant activity in the DPPH and NO radical scavenging assays was observed for **3a** with IC_50_ values of 21.6 ± 0.32 µM and 18.9 ± 0.54 µM, respectively. The presence of a moderately π-electron delocalizing fluorine atom at the *para* position of the styryl arm of **3b** with significant α-glucosidase inhibitory effect, on the other hand, resulted in significantly increased free radical scavenging activity with IC_50_ values of 6.6 ± 0.33 µM and 5.3 ± 0.34 µM in the DPPH and NO assays, respectively. Reduced DPPH and NO free radical scavenging activities were observed for **3c** with a combination of the 5′-(4-chlorostyryl) arm and 2′-amino-3-fluorochalcone framework with IC_50_ values of 15.3 ± 0.10 µM and 10.6 ± 0.30 µM, respectively. A strongly π-delocalization 4-methoxystyryl wing linked to position-5 of the 2′-amino-3-fluorochalcone framework of **3d** resulted in significantly increased free radical scavenging activity for this compound with an IC_50_ value of 3.9 ± 0.21 µM. The most active 2′-amino-5′-styryl-4-fluorochalcone **3e** against both carbohydrate hydrolyzing enzymes was found to exhibit comparable free radical scavenging activity to ascorbic acid in the DPPH assay with an IC_50_ value of 4.2 ± 0.43 µM. However, **3e** exhibited significantly reduced NO radical scavenging activity with an IC_50_ value of 20.5 ± 0.46 µM. Reduced antioxidant activity was observed for 2′-amino-5′-(4-fluorostyryl)-4-fluorochalcone **3f** in the DPPH assay with an IC_50_ value of 18.5 ± 0.37 µM. However, this compound exhibited significant NO radical scavenging activity with an IC_50_ value of 8.8 ± 0.33 µM. A combination of 5′-(4-chlorostyryl) group and 2′-amino-4-fluorochalcone scaffold resulted in significant DPPH scavenging activity for **3g** with an IC_50_ value of 5.2 ± 0.53 µM. This compound was, however, found to exhibit reduced NO radical scavenging activity with an IC_50_ value of 15.1 ± 0.19 µM. The presence of a strongly electron donating 5′-(4-methoxystyryl) arm on the scaffold of the 2′-amino-4-fluorochalcone **3h** resulted in significantly reduced activity for this compound in both the DPPH (IC_50_ = 25.8 ± 0.43 µM) and NO (IC_50_ = 20.2 ± 0.23 µM) radical scavenging assays. The observed free radical scavenging activities of these molecular hybrids is presumably due to the presence of styryl arm on the intramolecularly hydrogen bonded 2-aminochalcone scaffold.

Compounds **2a** and **3e** which exhibited relatively higher inhibitory effect against α-glucosidase and α-amylase activities and free radical scavenging properties, were in turn, subjected to kinetic studies on both enzymes to elucidate the plausible molecular mechanism of inhibition.

### 2.3. Kinetic Studies on **2a** and **3e**

**Figure 3:**
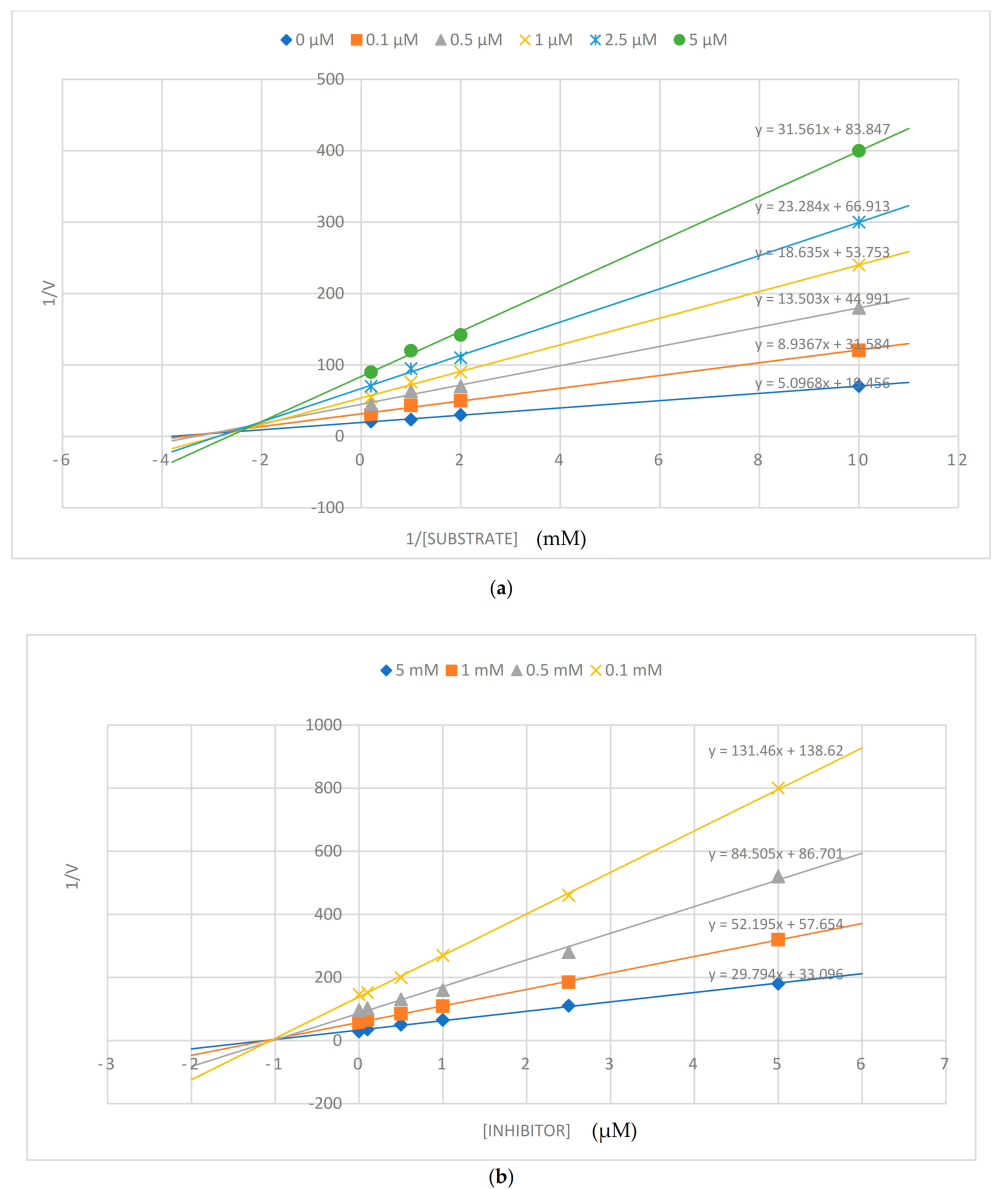
Lineweaver-Burk (**a**) and Dixon (**b**) plots for **2a** against α-glucosidase.

**Figure 4:**
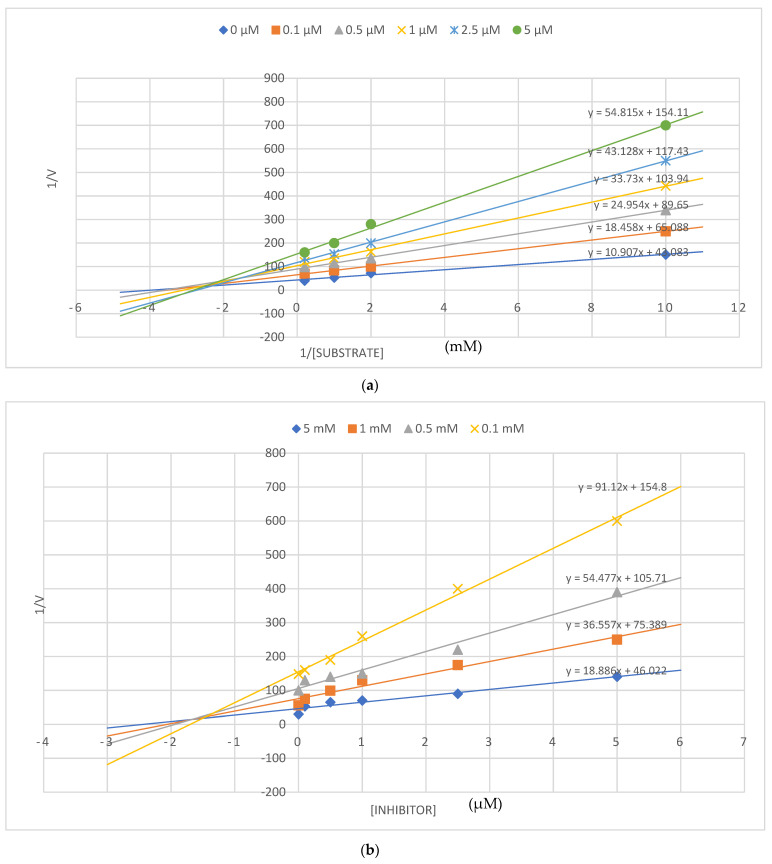
Lineweaver-Burk (**a**) and Dixon (**b**) plots for **3e** against α-glucosidase.

The enzyme mode of inhibition of compounds **2a** and **3e** against α-glucosidase and α-amylase were evaluated by constructing the Lineweaver-Burk and the Dixon plots at increasing substrate and inhibitor concentrations. The Lineweaver-Burk plot of 1/V versus 1/[S] in the presence of different concentrations of compound **2a** in the case of α-glucosidase gave a series of straight lines that intersect on the x-axis (Figure 3a). The plot shows an unchanged Michaelis constant (K_m_) value of 0.31 ± 0.02 and a decrease in the velocity of the reaction (V_max_) values (0.051–0.012 µM/min). The Dixon plot (Figure 3b) has straight lines that intersect on the x-axis with the calculated K*_i_* value of 0.30 ± 0.04 μM, and this observation is associated with non-competitive mode of inhibition confirming the Lineweaver-Burk data. Compound **3e**, on the other hand, showed increasing K_m_ values 0.25–0.35) with relatively unchanged V_max_ value of 0.01 ± 0.002 µM/min (Figure 4a). The Dixon plot of **3e** (Figure 4b) showed set of straight lines intersecting above the x-axis with K*i* value of 1.48 ± 0.05 μM. Both the Lineweaver-Burk and the Dixon plots for compound **3e** suggested a mixed mode of inhibition for this compound. The compound probably binds to the active site and other allosteric sites on the enzyme.

#### 2.3.1. Molecular Docking Studies

Molecular docking (in silico) has been employed to obtain a theoretical/hypothetical model for potential binding modes of the test compounds against α-glucosidase and α-amylase binding sites. The estimated binding free energy of these compounds (refer to Table 3) show that **2b**, **3a**, **3b**–**d** and **3h** were the least favorable ligands against α-glucosidase. On the other hand, derivatives **2b**, **2d** and **3h** represent the least favored for binding into α-amylase. Compounds **3e** and **3g** are predicted to have the strongest and comparable binding affinities against α-amylase and these values correlate with the above-mentioned inhibition assay results.

**Figure 5:**
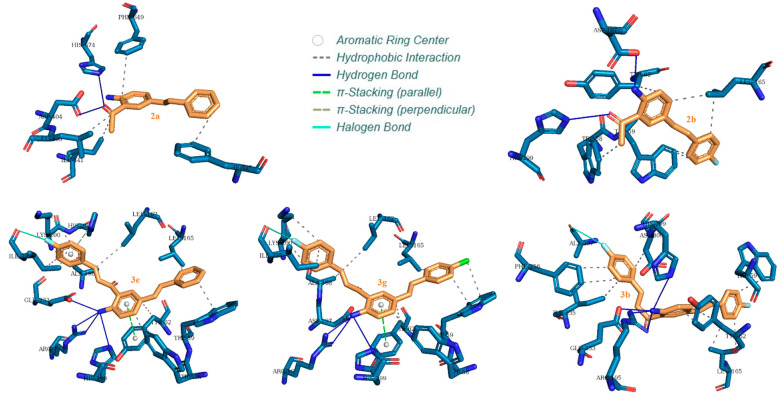
The interaction between the most (**2a**, **3e** and **3g**) and the least (**2b** and **3b**) active compounds against α-glucosidase.

**Figure 6:**
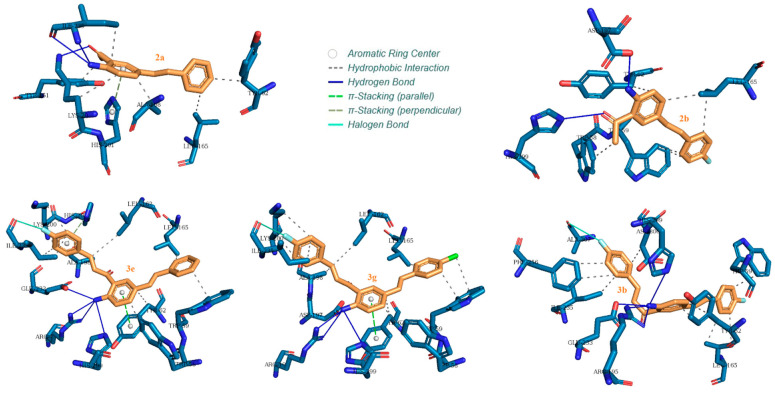
The interaction between the most (**2a**, **3e** and **3g**) and the least (**2b** and **3b**) active compounds against α-amylase.

The interactions between the test compounds and the enzymes were further analysed to compare their conformation in the binding site of α-glucosidase and α-amylase. The smaller size and volume of compounds **2a** and **2b** resulted in lower number of hydrophobic interactions with α-glucosidase compared to **3b**, **3e** and **3g** (Figure 5). A similar trend is observed for **2c** and **2d** compared to derivatives **3a**, **3c**, **3d**, **3f** and **3h** (Figure S3). It is also interesting to note that the most favorable compound in the first series, i.e., **2a** and series **3** (namely, **3e** and **3g**) form π–π stacking interaction with the side chain of α-amylase (Figure 6). Such π–π stacking interaction was not observed in the least favorable compounds (**2b** and **3b**). Relatively reduced hydrophobic interactions were observed for the smaller **2c** and **2d** compared to hybrids **3a**, **3c**, **3d**, **3f** and **3h** in α-amylase (Figure S4). The hydrogen bond was mainly contributed by the carbonyl group of the test compounds. 

#### 2.3.2. Prediction of Pharmacokinetic Properties of Compounds **2a**, **3e** and **3g**

In the last part of this investigation, we predicted the drug-likeness of the most active compounds using an in silico method. Drug-likeness is a complex balance of various molecular properties such as hydrophobicity, electronic distribution, hydrogen bonding characteristics, molecule size and flexibility and the presence of various pharmacophoric features [36]. In silico prediction of properties such as oral absorption, blood-brain barrier penetration, toxicity, metabolism, aqueous solubility, logP, pKa, half-life, and plasma protein binding contribute significantly to drug’s success in the drug discovery process, and minimizes the pharmacokinetic failures at various clinical phase. Bioavailability of the most active compound from each series, namely, **2a**, **3e** and **3g** is predicted at theoretical level through the Lipinski rule of five (molecular weight (< 500), hydrogen-bond donor (< 5), hydrogen-bond acceptor (< 10) and cLogP (< 5), which describes the relationship between physicochemical and pharmacokinetic properties of drugs (Table 4). Compound **2a** did not violate any Lipinski rule of five, but compounds **3e** and **3g** violate one rule.

Metabolic diseases such as diabetes require a stable long-term therapy accompanied by well tolerated and low toxicity, and this encouraged us to evaluate the safety profile of **2a** and **3e** as potential antidiabetic agents at least in vitro. The compounds were evaluated for cytotoxicity against the normal monkey kidney cells (Vero cells) and the adenocarcinomic human epithelial (A549) cell line using the 3-(4,5-dimethylthiazol-2-yl)-2, 5-diphenyltetrazolium (MTT) assay. Preliminary results of this study (Table 5) revealed no effect of these compounds on the viability of the Vero and A549 cells compared doxorubicin used as a reference standard at the same concentrations.

## 3. Materials and Methods

### 3.1. Instrumentation

The melting point values of the test compounds were recorded on a Thermocouple digital melting point apparatus (Mettler Toledo LLC, Columbus, OH, USA). The infrared (IR) spectra were recorded using the thin-film method on a Bruker VERTEX 70 FT-IR Spectrometer (Bruker Optics, Billerica, MA, USA) equipped with an ATR (diamond attenuated total reflectance) accessory. Merck kieselgel 60 (0.063–0.200 mm) (Merck KGaA, Frankfurt, Germany) was used as a stationary phase for column chromatography. The ^1^H NMR and ^13^C NMR spectra were recorded as deuterated chloroform (CDCl_3_) or dimethyl sulfoxide ((CD_3_)_2_SO) solutions using Agilent 500 MHz NMR spectrometer (Agilent Technologies, Oxford, UK) operating at 500 MHz and 125 MHz for ^1^H and ^13^C, respectively. The chemical shifts are quoted relative to tetramethylsilane (TMS) used as an internal reference standard (δ = 0.00 ppm) or to residual protonated solvent. Data are presented as follows: chemical shift (ppm), multiplicity (s = singlet, d = doublet, t = triplet, q = quartet, sept = septet, m = multiplet, br = broad), coupling constant *J* (Hz), and integration. The high-resolution mass spectra were recorded at an ionization potential of 70 eV using Micromass Autospec-TOF (double focusing high resolution) instrument (Waters Corp., Milford, MA, USA).

### 3.2. Synthesis of 2-Amino-5-Iodoacetophenone (**1**)

A stirred solution of 2-aminoacetophenone (5.00 g, 37.04 mmol, purity = 98%) in acetonitrile (40 mL) was treated slowly with *N*-iodosuccinimide (8.32 g, 37.04 mmol, purity = 95%) at room temperature. The mixture was stirred at room temperature for 1 h, and then poured into an ice-cold saturated aqueous solution of sodium thiosulphate. The resultant precipitate was filtered, washed with an ice cold water, and recrystallized from ethanol to afford 2-amino-5-iodoacetophenone as a brown solid (8.70 g, 90%), mp 97−98 °C; FTIR (ATR) ν_max_ = 517, 623, 671, 738, 821, 887, 956, 1292, 1310, 1419, 1460, 1530, 1572, 1602, 1627 (C=O), 3310, 3424 (NH_2_) cm^−1^; ^1^H NMR (CDCl_3_) δ = 2.73 (3H, s, -CH_3_), 5.73 (1H, br s, NH_2_), 6.64 (1H, d, *J* = 8.7 Hz, H-3), 7.65 (1H, d, *J* = 8.7 Hz, H-4), 8.14 (1H, s, H-6); ^13^C NMR (CDCl_3_) δ = 28.1, 75.4, 119.6, 120.5, 140.5, 142.6, 149.7, 199.8 (C=O).

### 3.3. Typical Procedure for the Synthesis of 2-Amino-5-Styrylacetophenones **2a**–**d**

A mixture of **1** (0.50 g, 1.92 mmol), PdCl_2_(PPh_3_)_2_ (0.07 g, 0.11 mmol, purity ≥ 99%), PCy_3_ (0.27 g, 0.20 mmol, purity = 95%) and K_2_CO_3_ (0.27 g, 2.30 mmol, purity ≥ 99%) and phenylboronic acid (0.40 g, 2.30 mmol, purity = 95%) in 3:1 DMF–water (*v*/*v*; 20 mL) in a two-necked round bottom flask equipped with a condenser and rubber septum was purged with nitrogen gas for 30 min. A balloon filled with nitrogen gas was connected to the top of the condenser, and the reaction mixture was stirred at 70 °C for 3 h. The mixture was quenched with an ice-cold water, and the product was extracted into chloroform. The combined organic layers were washed with water and dried over anhydrous MgSO_4_. The salt was filtered off and the solvent was evaporated under reduced pressure on a rotary evaporator. The residue was purified by column chromatography on silica gel using 2:1 toluene-ethyl acetate (*v*/*v*) mixture as an eluent. Compounds **2a**–**d** were prepared in this fashion.

(*E*)-1-(2-Amino-5-styrylphenyl)ethan-1-one (**2a**)

Yellow solid (0.33 g, 73%), mp. 119–120 °C; FTIR (ATR) ν_max_ = 418, 537, 688, 818, 947, 1169, 1194, 1213, 1422, 1494, 1545, 1572, 1618, 1649, 3343, 3473 cm^−1^; ^1^H NMR (DMSO-*d*_6_) δ = 2.86 (3H, s, -CH_3_), 7.01 (1H, d, *J* = 8.5 Hz, H-4′), 7.29 (1H, d, *J*_trans_ = 16.3 Hz, H-α), 7.43 (1H, d, *J*_trans_ = 17.0 Hz, H-β), 7.63 (3H, t, *J* = 7.5 Hz, H-3′,5′), 7.68 (2H, s, -NH_2_), 7.81 (2H, d, *J* = 7.0 Hz, H-2′,6′), 7.90 (1H, d, *J* = 8.0 Hz), 8.20 (1H, s, H-6); ^13^C NMR (DMSO-*d*_6_) δ = 28.3, 117.9, 124.1, 124.5, 126.3, 127.2, 128.8, 129.1, 129.2, 131.0, 132.0, 138.2, 151.2, 200.6; HRMS (ES): m/z [M + H]^+^ calc for C_16_H_16_NO: 238.1232; found 238.1233.

(*E*)-1-(2-Amino-5-(4-fluorostyryl)phenyl)ethan-1-one (**2b**)

Yellow solid (0.34 g, 70%), mp. 149–150 °C; FTIR (ATR) ν_max_ = 534, 598, 830, 926, 1209, 1226, 1496, 1506, 1544, 1570, 1614, 1650, 3347, 3482 cm^−1^; ^1^H NMR (DMSO-*d*_6_) δ = 2.63 (3H, s, -CH_3_), 6.40 (2H, s, -NH_2_), 6.66 (1H, d, *J* = 8.6 Hz, H-3), 6.85 (1H, d, *J*_trans_ = 16.3 Hz, H-α), 6.93 (1H, d, *J*_trans_ = 16.3 Hz, H-β), 7.03 (2H, t, *J* = 8.7 Hz, H-3′,5′), 7.44 (2H, dd, *J* = 5.4 Hz and 8.7 Hz, H-2′,6′), 7.52 (1H, dd, *J* = 2.0 Hz and 8.6 Hz, H-4), 7.76 (1H, d, *J* = 2.0 Hz, H-6); ^13^C NMR (DMSO-*d*_6_) δ = 27.9, 115.6 (d, ^2^*J*_CF_ = 21.3 Hz), 117.8, 118.0, 124.2, 125.2, 127.5 (d, ^3^*J*_CF_ = 7.9 Hz), 127.7, 127.8, 130.9, 131.7, 133.8, (d, ^4^*J*_CF_ = 2.2 Hz), 149.9, 162.0 (d, ^1^*J*_CF_ = 245.0 Hz), 200.7; HRMS (ES): m/z [M + H]^+^ calc for C_16_H_15_FNO: 256.1138; found 256.1141.

(*E*)-1-(2-Amino-5-(4-chlorostyryl)phenyl)ethan-1-one (**2c**)

Yellow solid (0.41 g, 78%), mp. 137–138 °C; FTIR (ATR) ν_max_ = 567, 625, 817, 851, 964, 1191, 1215, 1426, 1507, 1546, 1616, 1642, 3348, 3482 cm^−1^; ^1^H NMR (DMSO-*d*_6_) δ = 2.62 (3H, s, -CH_3_), 6.41 (2H, s, -NH_2_), 6.65 (1H, d, *J* = 8.6 Hz, H-3), 6.83 (1H, d, *J*_trans_ = 16.3 Hz, H-α), 7.00 (1H, d, *J*_trans_ = 16.3 Hz, H-β), 7.30 (2H, d, *J* = 8.5 Hz, H-3′,5′), 7.39 (2H, d, *J* = 8.5 Hz, H-2′,6′), 7.51 (1H, dd, *J* = 2.0 Hz and 8.6 Hz, H-4), 7.75 (1H, d, *J* = 2.0 Hz, H-6); ^13^C NMR (DMSO-*d*_6_) δ = 27.9, 117.8, 118.0, 124.0, 125.0, 127.6, 128.6, 128.9, 131.1, 131.8, 132.6, 136.1, 150.1, 200.7; HRMS (ES): m/z [M + H]^+^ calc for C_16_H_15_ClNO: 272.0848; found 272.0842.

(*E*)-1-(2-Amino-5-(4-methoxystyryl)phenyl)ethan-1-one (**2d**)

Yellow solid (0.35 g, 69%), mp. 139–140 °C; FTIR (ATR) ν_max_ = 520, 806, 851, 956, 1029, 1243, 1511, 1619, 1651, 3357, 3490 cm^−1^; ^1^H NMR (DMSO-*d*_6_) δ = 2.55 (3H, s, -CH_3_), 3.75 (3H, s, -OCH_3_), 6.77 (1H, d, *J* = 8.5 Hz, H-3), 6.90 (2H, d, *J* = 8.5 Hz, H-3′,5′), 6.93 (1H, d, *J*_trans_ = 16.5 Hz, H-α), 7.00 (1H, d, *J*_trans_ = 16.5 Hz, H-β), 7.33 (2H, s, -NH_2_), 7.45 (2H, d, *J* = 8.5 Hz, H-2′,6′), 7.57 (1H, dd, *J* = 2.0 Hz and 9.0 Hz, H-4), 7.84 (1H, d, *J* = 1.5 Hz, H-6); ^13^C NMR (DMSO-*d*_6_) δ = 28.4, 55.4, 114.6, 117.1, 117.9, 124.2, 124.5, 126.5, 127.5, 130.8, 131.3, 131.8, 150.9, 158.8, 200.6; HRMS (ES): m/z [M + H]^+^ calc for C_17_H_18_NO_2_: 268.1338; found 268.1337.

### 3.4. Typical Procedure for the Claisen-Schmidt Aldol Condensation of **2a**–**d** to Afford **3a–h**

A mixture of **2** (1 equiv.), 4-fluorobenzaldehyde (1.2 equiv., purity = 98%) and KOH (3 pellets, purity ≥ 85%) in absolute ethanol (2.5 mL/mmol of **2**) was stirred for 24 h at room temperature, and then quenched with an ice-cold water. The precipitate was filtered and purified by column chromatography on silica gel using toluene as an eluent. The following products were prepared in this fashion.

(*E*)-1-(2-Amino-5-((E)-styryl)phenyl)-3-(3-fluorophenyl)prop-2-en-1-one (**3a**)

Orange solid (0.09 g, 66%), mp. 129–130 °C; FTIR (ATR) ν_max_ = 534, 622, 701, 826, 954, 1189, 1210, 1545, 1567, 1614, 1649, 2922, 3347, 3465 cm^−1^; ^1^H NMR (DMSO-*d*_6_) δ = 6.91 (1H, d, *J* = 9.0 Hz, H-3), 7.06 (1H, d, *J*_trans_ = 16.0 Hz, H-α), 7.28 (1H, d, *J* = 8.5 Hz, H-6′), 7.29 (1H, d, *J*_trans_ = 16.5 Hz, H-β), 7.34 (1H, t, *J* = 9.0, H-4”), 7.42 (2H, t, *J* = 7.5, H-4”), 7.56 (1H, d, *J* = 8.0 Hz, H-2′), 7.60 (2H, d, *J* = 8.0 Hz, H-2′), 7.71 (2H, s, NH_2_), 7.73 (1H, d, *J*_trans_ = 15.5 Hz, H-α′), 7.75 (2H, t, *J* = 7.5, H-4”), 7.91 (1H, dd, *J* = 6.0 Hz and 9.0 Hz, H-4), 8.15 (1H, d, *J*_trans_ = 15.0 Hz, H-β′), 8.30 (1H, s, H-6); ^13^C NMR (DMSO-*d*_6_) δ = 115.3 (d, ^2^*J*_CF_ = 21.75 Hz), 117.5 (d, ^2^*J*_CF_ = 20.75 Hz), 117.7, 118.3, 124.6, 124.8, 125.4, 126.1 (d, ^4^*J*_CF_ = 2.0 Hz), 126.5, 127.5, 129.1, 129.4, 131.5, 131.6 (d, ^4^*J*_CF_ = 1.9 Hz), 132.3, 138.3 (d, ^3^*J*_CF_ = 7.6 Hz), 138.5, 141.4 (d, ^4^*J*_CF_ = 2.75 Hz), 152.7, 136.2 (d, ^1^*J*_CF_ = 242.5 Hz), 190.9; HRMS (ES): m/z [M + H]^+^ calc for C_23_H_19_FNO: 344.1451; found 344.1451.

(*E*)-1-(2-Amino-5-((E)-4-fluorostyryl)phenyl)-3-(3-fluorophenyl)prop-2-en-1-one (**3b**)

Orange solid (0.10 g, 73%), mp. 175–177 °C; FTIR (ATR) ν_max_ = 468, 544, 790, 827, 999, 1168, 1186, 1241, 1484, 1507, 1567, 1616, 1646, 3391, 3480 cm^−1^; ^1^HNMR (DMSO-*d*_6_) δ = 6.84 (1H, d, *J* = 8.6 Hz, H-3), 7.03 (1H, d, *J*_trans_ = 16.3 Hz, H-α), 7.14 (1H, d, *J*_trans_ = 17.0 Hz, H-β), 7.18 (2H, t, *J* = 8.5 Hz, H-3′,5′), 7.26 (1H, t, *J* = 9.5 Hz, H-6”), 7.48 (1H, dd, *J* = 2.0 Hz and 7.5 Hz, H-2”), 7.44 (2H, dd, *J* = 5.5 Hz and 8.5 Hz, H-2′,6′), 7.63 (2H, s, -NH_2_), 7.66 (1H, d, *J*_trans_ = 17.0 Hz, H-α′), 7.65–7.68 (2H, m, Ph), 7.83 (1H, dd, *J* = 2.0 Hz and 9.0 Hz, H-4), 8.04 (1H, d, *J*_trans_ = 15.5 Hz, H-β′), 8.20 (1H, s, H-6); ^13^C NMR (DMSO-*d*_6_) δ = 114.9 (d, ^2^*J*_CF_ = 21.8 Hz), 115.9 (d, ^2^*J*_CF_ = 21.8 Hz), 117.2 (d, ^2^*J*_CF_ = 21.8 Hz), 117.5, 118.1, 123.5, 124.3, 125.2, 125.8, 128.0 (d, ^3^*J*_CF_ = 7.6 Hz), 128.8, 131.2 (d, ^3^*J*_CF_ = 9.5 Hz), 132.0, 134.8 (d, ^4^*J*_CF_ = 2.9 Hz), 138.1 (d, ^3^*J*_CF_ = 7.5 Hz), 141.2 (d, ^4^*J*_CF_ = 2.9 Hz), 152.4, 161.3 (d, ^1^*J*_CF_ = 242.8 Hz), 163.0 (d, ^1^*J*_CF_ = 241.75 Hz), 190.6; HRMS (ES): m/z [M + H]^+^ calc for C_23_H_18_F_2_NO: 362.1356; found 362.1348. 

(*E*)-1-(2-Amino-5-((E)-4-Chlorostyryl)phenyl)-3-(3-fluorophenyl)prop-2-en-1-one (**3c**)

Orange solid (0.10 g, 74%), mp. 187–188 °C; FTIR (ATR) ν_max_ = 433, 542, 775, 847, 954, 1170, 1210, 1245, 1260, 1544, 1570, 1615, 1645, 2852, 2930. 3325, 3478 cm^−1^; ^1^H NMR (DMSO-*d*_6_) δ = 6.85 (1H, d, *J* = 7.5 Hz, H-3), 7.03 (1H, d, *J*_trans_ = 16.5 Hz, H-α), 7.22 (1H, d, *J*_trans_ = 17.0 Hz, H-β), 7.26 (2H, t, *J* = 2.0 Hz, H-2”,6”), 7.39 (2H, d, *J* = 8.5 Hz, H-3′,5′), 7.50 (1H, dd, *J* = 2.0 Hz and 7.5 Hz, H-4”), 7.54 (2H, d, *J* = 8.5 Hz, H-2′,6′), 7.63 (2H, s, -NH_2_), 7.64 (1H, d, *J*_trans_ = 15.5 Hz, H-α′), 7.66 (1H, t, *J* = 8.5 Hz, H-5”), 7.83 (1H, dd, *J* = 2.0 Hz and 9.0 Hz, H-4), 8.07 (1H, d, *J*_trans_ = 15.5 Hz, H-β′), 8.30 (1H, s, H-6); ^13^C NMR (DMSO-*d*_6_) δ = 114.9 (d, ^2^*J*_CF_ = 20.9 Hz), 117.2 (d, ^2^*J*_CF_ = 20.9 Hz), 117.5, 118.1, 123.2, 124.1, 125.2, 125.8, 125.8, 127.9, 129.1, 129.8, 131.3 (d, ^3^*J*_CF_ = 7.6 Hz), 131.5, 132.1, 137.2, 138.1 (d, ^3^*J*_CF_ = 7.6 Hz), 141.2 (d, ^4^*J*_CF_ = 1.9 Hz), 152.5, 163.1 (d, ^1^*J*_CF_ = 242.8 Hz), 190.6; HRMS (ES): m/z [M + H]^+^ calc for C_23_H_18_ClFNO: 378.1061; found 378.1063.

(*E*)-1-(2-Amino-5-((E)-4-methoxystyryl)phenyl)-3-(3-fluorophenyl)prop-2-en-1-one (**3d**)

Orange solid (0.10 g, 73%), mp. 154–155 °C; FTIR (ATR) ν_max_ = 534, 685, 782, 824, 955, 1165, 1211, 1245, 1508, 1614, 1646, 3328, 3480 cm^−1^; ^1^H NMR (DMSO-*d*_6_) δ = 3.75 (3H, s, -OCH_3_), 6.84 (1H, d, *J* = 8.5 Hz, H-3), 6.91 (2H, d, *J* = 8.5 Hz, H-3′,5′), 7.00 (1H, d, *J*_trans_ = 16.0 Hz, H-α), 7.03 (1H, d, *J*_trans_ = 16.5 Hz, H-β), 7.25 (1H, t, *J* = 8.0 Hz, H-5”), 7.46 (2H, d, *J* = 9.0 Hz, H-2′,6′), 7.50 (1H, d, *J* = 7.5 Hz, H-6”), 7.58 (2H, s, -NH_2_), 7.62–7.65 (2H, m, Ar), 7.66 (1H, d, *J*_trans_ = 15.0 Hz, H-α′), 7.84 (1H, dd, *J* = 2.0 Hz and 9.0 Hz, H-4), 8.07 (1H, d, *J*_trans_ = 15.0 Hz, H-β′), 8.16 (1H, d, *J* = 1.5 Hz, H-6); ^13^C NMR (DMSO-*d*_6_) δ = 55.6, 114.6, 114.9 (d, ^2^*J*_CF_ = 20.7 Hz), 117.3 (d, ^2^*J*_CF_ = 20.87 Hz), 117.5, 118.1, 124.4, 124.7, 125.2, 125.8 (d, ^4^*J*_CF_ = 1.9 Hz), 126.9, 127.5, 130.7, 130.8, 131.6 (d, ^3^*J*_CF_ = 7.6 Hz), 138.1 (d, ^3^*J*_CF_ = 7.6 Hz), 141.1, 141.2, 152.1, 158.9, 163.2 (d, ^1^*J*_CF_ = 241.9 Hz), 190.6; HRMS (ES): m/z [M + H]^+^ calc for C_24_H_21_FNO_2_: 374.1556; found 374.1557.

(*E*)-1-(2-Amino-5-((E)-styryl)phenyl)-3-(4-fluorophenyl)prop-2-en-1-one (**3e**)

Orange solid (0.09 g, 69%), mp. 161–162 °C; FTIR (ATR) ν_max_ = 422, 524, 714, 822, 953, 1169, 1210, 1469, 1545, 1595, 1616, 1649, 3347, 3479 cm^−1^; ^1^H NMR (DMSO-*d*_6_) δ = 6.85 (1H, d, *J* = 8.5 Hz, H-3), 7.04 (1H, d, *J*_trans_ = 16.0 Hz, H-α), 7.07 (1H, d, *J* = 9.0 Hz, H-4), 7.20 (1H, t, *J* = 4.5 Hz, H-3′), 7.22 (1H, d, *J*_trans_ = 16.5 Hz, H-β), 7.28 (2H, t, *J* = 9.0 Hz, H-3″,5″), 7.34 (1H, t, *J* = 8.0 Hz, H-4′), 7.53 (2H, d, *J* = 8.5 Hz, H-3′,5′), 7.61 (2H, s, NH_2_), 7.63 (1H, d, *J*_trans_ = 15.5 Hz, H-α′), 7.83 (1H, d, *J* = 9.0 Hz, H-5′), 7.96 (2H, dd, *J* = 6.0 Hz and 9.0 Hz, H-2″,6″), 8.00 (1H, d, *J*_trans_ = 15.5 Hz, H-β′), 8.21 (1H, s, H-6); ^13^C NMR (DMSO-*d*_6_) δ = 116.2 (d, ^2^*J*_CF_ = 20.8 Hz), 117.6, 118.1, 124.3, 124.5, 126.3, 127.2, 128.9, 129.1, 131.2, 131.4 (d, ^3^*J*_CF_ = 8.5 Hz), 131.9, 132.2 (d, ^4^*J*_CF_ = 2.9 Hz), 132.3, 138.2, 141.4, 152.3, 163.4 (d, ^1^*J*_CF_ = 246.5 Hz), 190.7; HRMS (ES): m/z [M + H]^+^ calc for C_23_H_19_FNO: 344.1451; found 344.1457.

(*E*)-1-(2-Amino-5-((E)-4-fluorostyryl)phenyl)-3-(4-fluorophenyl)prop-2-en-1-one (**3f**)

Orange solid (0.10 g, 70%), mp. 181–182 °C; FTIR (ATR) ν_max_ = 471, 534, 827, 954, 1158, 1179, 1217, 1506, 1566, 1615, 1648, 3309, 3493 cm^−1^; ^1^H NMR (DMSO-*d*_6_) δ = 6.46 (2H, s, NH_2_), 6.72 (1H, d, *J* = 8.6 Hz, H-3), 6.90 (1H, d, *J*_trans_ = 16.3 Hz, H-α), 7.00 (1H, d, *J*_trans_ = 16.3 Hz, H-β), 7.04 (2H, t, *J* = 8.7 Hz, H-3′,5′), 7.13 (2H, t, *J* = 8.7 Hz, H-3″,5″), 7.45 (2H, dd, *J* = 5.4 Hz and 8.7 Hz, H-2′,6′), 7.57 (1H, dd, *J* = 2.0 Hz and 8.7 Hz, H-4), 7.62 (1H, d, *J*_trans_ = 16.3 Hz, H-α′), 7.66 (2H, dd, *J* = 5.4 Hz and 8.7 Hz, H-2″,6″), 7.73 (1H, d, *J*_trans_ = 16.3 Hz, H-β′), 7.86 (1H, d, *J* = 2.0 Hz, H-6); ^13^C NMR (DMSO-*d*_6_) δ = 115.6 (d, ^2^*J*_CF_ = 21.0 Hz), 116.1 (d, ^2^*J*_CF_ = 21.9 Hz), 117.9, 118.7, 122.5 (d, ^4^*J*_CF_ = 2.8), 124.3, 125.4, 127.6 (d, ^3^*J*_CF_ = 8.0 Hz), 127.8 (d, ^4^*J*_CF_ = 2.1), 129.8, 130.1 (d, ^3^*J*_CF_ = 8.3 Hz), 131.4, 131.6, 133.8, 142.0, 150.7, 162.1 (d, ^1^*J*_CF_ = 245.1 Hz), 163.9 (d, ^1^*J*_CF_ = 249.8 Hz), 191.3; HRMS (ES): m/z [M + H]^+^ calc for C_23_H_18_F_2_NO: 362.1356; found 362.1357.

(*E*)-1-(2-Amino-5-((E)-4-chlorostyryl)phenyl)-3-(4-fluorophenyl)prop-2-en-1-one (**3g**)

Orange solid (0.09 g, 74%), mp. 197–198 °C; FTIR (ATR) ν_max_ = 471, 549, 829, 1180, 1223, 1279, 1530, 1650, 3369, 3494 cm^−1^; ^1^H NMR (DMSO-*d*_6_) δ = 6.48 (2H, s, NH_2_), 6.72 (1H, d, *J* = 8.6 Hz, H-3), 6.87 (1H, d, *J*_trans_ = 16.3 Hz, H-α), 7.00 (1H, d, *J*_trans_ = 16.3 Hz, H-β), 7.12 (2H, t, *J* = 8.6 Hz, H-3″,5″), 7.30 (2H, d, *J* = 8.7 Hz, H-3′,5′), 7.41 (2H, d, *J* = 8.5 Hz, H-2′,6′), 7.56 (1H, dd, *J* = 1.9 Hz and 8.0 Hz, H-4), 7.57 (1H, d, *J*_trans_ = 16.3 Hz, H-α′), 7.66 (2H, dd, *J* = 5.4 Hz and 8.7 Hz, H-2″,6″), 7.73 (1H, d, *J*_trans_ = 15.5 Hz, H-β′), 7.87 (1H, d, *J* = 1.8 Hz, H-6); ^13^C NMR (DMSO-*d*_6_) δ = 116.1 (d, ^2^*J*_CF_ = 22.6 Hz), 118.0, 118.7, 122.5 (d, ^4^*J*_CF_ = 3.0), 124.2, 125.2, 127.3, 128.6, 128.8, 130.1, 130.3 (d, ^3^*J*_CF_ = 8.5 Hz), 131.4, 131.6, 132.6, 136.1, 142.1, 150.8, 163.9 (d, ^1^*J*_CF_ = 249.7 Hz), 191.3; HRMS (ES): m/z [M + H]^+^ calc for C_23_H_18_ClFNO: 378.1061; found 378.1062.

(*E*)-1-(2-Amino-5-((E)-4-methoxystyryl)phenyl)-3-(4-fluorophenyl)prop-2-en-1-one (**3h**)

Orange solid (0.11 g, 73%), mp. 153–154 °C; FTIR (ATR) ν_max_ = 446, 472, 548, 827, 959, 1008, 1179, 1209, 1505, 1543, 1615, 1647, 3309, 3510 cm^−1^; ^1^H NMR (DMSO-*d*_6_) δ = 3.75 (3H, s, -OCH_3_), 6.84 (1H, d, *J* = 8.5 Hz, H-3), 6.91 (2H, d, *J* = 8.5 Hz, H-3′,5′), 7.00 (1H, d, *J*_trans_ = 16.5 Hz, H-α), 7.01 (1H, d, *J*_trans_ = 15.5 Hz, H-β), 7.28 (2H, t, *J* = 9.0 Hz, H-3″,5″), 7.46 (2H, d, *J* = 8.5 Hz, H-2′,6′), 7.57 (2H, s, NH_2_), 7.62 (1H, d, *J* = 9.0 Hz, H-4), 7.67 (1H, d, *J*_trans_ = 15.5 Hz, H-α′), 7.96 (2H, dd, *J* = 6.0 Hz and 9.0 Hz, H-2″,6″), 8.00 (1H, d, *J*_trans_ = 15.5 Hz, H-β′), 8.17 (1H, s, H-6); ^13^CNMR (DMSO-*d*_6_) δ = 55.5, 114.6, 116.2 (d, ^2^*J*_CF_ = 21.9 Hz), 117.6, 118.0, 123.6, 124.3, 124.7, 126.7, 127.5, 130.6, 130.9, 131.4 (d, ^3^*J*_CF_ = 8.5 Hz), 131.8, 132.2 (d, ^4^*J*_CF_ = 2.87), 141.3, 152.0, 158.8, 163.4 (d, ^1^*J*_CF_ = 246.5 Hz), 190.8; HRMS (ES): m/z [M + H]^+^ calc for C_24_H_21_FNO_2_: 374.1556; found 374.1556.

### 3.5. α-Glucosidase Inhibition Assays of **2a–d** and **3a–h**

All the tests and analyses were performed in triplicates following a modified published protocol described in our previous study [37]. The stock solution (200 µM) of the test compounds and the reference standard, acarbose, were prepared in DMSO and further diluted with phosphate buffer (100 mM, pH 6.8) to obtain final concentrations of 1. 2.5, 5, 10, 25 and 50 µM. α-Glucosidase solution (0.48 u/mL, 20 µL), phosphate buffer (100 mM, pH 6.8; 30 µL) and the test sample (20 µL) as well as the reference standard were added in the designated wells, and the reaction mixtures were incubated at 37 °C for 10 min. 2 mM *p*-nitrophenyl-α-D-glucopyranoside (20 µL) was added to each of the wells containing reaction mixture to initiate the reaction. The reaction was pre-incubated for 30 min at 37 °C. The reaction was stopped by the addition of 20 μL of a solution of sodium carbonate (200 mM) to each well. Five absorbance readings were recorded for each triplicate run at a wavelength of 400 nm using Varioskan flash microplate spectrophotometer (Thermo Scientific, Waltham, MA, USA). The average values obtained from the readings were used to determine the IC_50_ and standard deviation values. The values were calculated by the nonlinear regression analysis and expressed as the mean SD of three distinct experiments using Graph Pad Prism software.

### 3.6. α-Amylase Inhibition Assays of **2a–d** and **3a–h**

The α-amylase assay was performed in triplicate using a 96-well plate following the procedure by the manufacturer as outlined in the α-Amylase Inhibitor Screening Kit (Catalog No. K482; Bio Vision). The stock solution (100 µM) of the test compounds, specific α-amylase inhibitor from *Triticum aestivum* and acarbose were prepared in DMSO, and further diluted with α-amylase assay buffer to obtain final concentrations of 1, 2.5, 5, 10, 25 and 50 µM. For inhibitor control (10 µL of α-amylase inhibitor and 40 µL of assay buffer were added to 3 wells), enzyme control (50 µL of assay buffer was added to three wells) and 50 µL of the test compounds were added into the remaining wells. A solution of α-amylase enzyme (50 µL) prepared by adding 490 µL of assay buffer to 10 µL of α-amylase enzyme was added to each of the wells containing the reaction mixture to initiate the reaction. The plate was incubated for 10 min at room temperature in the dark. Five different absorbance readings were recorded for each triplicate run at a wavelength of 405 nm using Varioskan flash microplate spectrophotometer (Thermo Scientific, Waltham, MA, USA). The IC_50_ and SD values were calculated using graph pad prism.

### 3.7. Free Radical Scavenging Assays

#### 3.7.1. Determination of Reducing Activity of the Stable DPPH Radical by **2a**–**d** and **3a**–**h**

The antioxidant activities of the test compounds against ascorbic acid (Sigma Aldrich, Saint Louis, Missouri, USA) as a positive control were evaluated using 2,2-diphenyl-1-picrylhydrazyl (DPPH) radical scavenging assay developed by Zhu *et al*. as described in our previous study [37]. Triplicate solutions (20 µL) of the test compounds and ascorbic acid in DMSO (final concentrations: 5, 10, 25, 50, and 100 µM) were added into each designated well of a 96-well plate. A solution of 0.20 mM DPPH (20 µL) in methanol was added to each well, and the 96-well plate was wrapped with aluminum foil and incubated in the dark for 45 min. Five absorbance readings were recorded at 512 nm using Varioskan flash microplate spectrophotometer reader. The average values obtained from the absorbance readings were used to determine the IC_50_ and standard deviation values.

#### 3.7.2. NO Free Radical Scavenging Assay

Nitric oxide was generated from sodium nitroprusside and measured by Griess’ reaction following the literature method [38]. The experiment was done in triplicate with ascorbic acid (vitamin C) used as a positive control for the assay. In a 96 well plate, 5 μL of the test compounds (0.1, 0.5, 1.0, 5.0 and 10.0 μM in methanol) or the positive control were mixed with 5 μL of 10 mM sodium nitroprusside prepared in phosphate buffered saline (pH = 7.4) and allowed to incubate at room temperature for 2.5 h. Afterwards, 30 μL of the Griess reagent (a mixture of 0.2% *N*-(1-naphthyl)ethylenediamine dihydrochloride and 2% sulfanilamide in 5%) was added to each well and the mixtures were and allowed to stand at room temperature for 30 min. Five absorbance readings were recorded at 546 nm using Varioskan flash microplate spectrophotometer reader.

### 3.8. Kinetic Studies 

Enzyme kinetics studies were performed on compounds **2a** and **3e** with increasing inhibitor and substrate concentrations. In the experimental procedure, inhibitors concentrations of 0.1, 0.5, 1, 2.5 µM, and final substrate concentration were 0.1, 0.5, 1 and 5 mM were used. 5 μL of the inhibitor and 2 μL of the enzyme (α-glucosidase) were added to each well of the 96-well plate. The reaction was incubated for 20 min at room temperature. 18 μL of the substrate, PNPG (4-nitrophenyl α-D-glucopyranoside) was then added and absorbance readings recorded after every 2 min at wavelength of 400 nm using a Varioskan flash microplate spectrophotometer reader. Lineweaver-Burk plot (the inverse of velocity (1/v) against the inverse of the substrate concentration (1/[S]) were used to determining the type of inhibition, while the inhibitor constant was obtained from the Dixon plot (the inverse of velocity (1/v) against concentration of inhibitor at each substrate concentration)

### 3.9. Molecular Docking of Test Compounds against α-Glucosidase and α-Amylase

We performed docking simulation of the test compounds against both α-glucosidase and α-amylase. The initial coordinates for the test compounds were generated using Avogadro program [39]. The polar hydrogen atoms of the test compounds were retained and Gasteiger charges and torsional angles were assigned by AutoDockTools [40]. The crystal structures of α-glucosidase (PDB code: 5NN8) and α-amylase (PDB code: 5E0F) were used as the acceptors for the test compounds. The heteroatoms and water molecules were first removed prior to the addition of polar hydrogen atoms. Kollman–Amber united atom partial charges and solvation parameters were then assigned using AutoDockTools. Docking simulation by AutoDock4.2.6 [40] was performed within 50 × 50 × 50 grid points centered at ligand active site. AutoDock4.2.6 was used to apply 100 Lamarckian genetic algorithm docking runs with the following parameters: energy evaluation of 2,500,000, maximum of 27,000 generation, population of 150, mutation rate of 0.02 and crossover rate of 0.8. Further interaction analysis using a Protein–Ligand Interaction Profiler was performed on the ligand in the most populated cluster with most favourable binding free energy.

### 3.10. Physicochemical Parameters of **2a**, **3e** and **3g**

The bioactivity score and the molecular properties of the test compounds were calculated by Molinspiration (www.molinspiration.com, accessed on 4 April 2021). The Lipinski’s rule of five was used to evaluate the drug-likeliness of the test compounds.

### 3.11. Evaluation of Cytotoxicity (MTT assay) of **2a** and **3e** on Vero and A549 Cells

The cytotoxicity activity of compounds **2a** and **3e** was evaluated against doxorubicin (positive control) using the 3-(4,5-dimethylthiazol-2-yl)-2,5-diphenyltetrazolium bromide (MTT) assay following a modification of a literature method by Mosmann [41]. The Vero and A549 cells were maintained in Dulbecco′s modified Eagle′s medium (DMEM; Gibco) supplemented with 10% Fetal bovine serum (FBS) and 1% penicillin/streptomycin in culture flasks and incubated at 37 °C in 5% CO_2_. When the cells reached 85% confluency, they were detached using 2% trypsin. Cell count was performed using a handheld automated cell counter (Scapter 3.0™, Merck). The cells were seeded at 1 × 10^5^ cells/well and incubated overnight to allow cell attachment. After 24 h, the cells were treated with different concentrations (0, 2.5, 5, 10, 25, 50, 100 µM) of the test compounds and the reference stand. The cells were incubated for 24 h followed by treatment with 20 µL of MTT solution (5 mg/mL). After 4 h incubation period, DMSO (100 µL) was added to each well to dissolve the formazan crystals. Five absorbance readings were recorded at 517 nm using Varioskan flash microplate spectrophotometer reader.

## 4. Conclusions

A new set of 5-styryl-2-aminochalcone hybrids has been synthesized and their molecular constructs confirmed using a combination of spectroscopic techniques complemented with single crystal X-ray diffraction (XRD) method. The co-planarity of the molecular frameworks of intermediates **2a**–**d** and their styryl-chalcone derivatives **3a**–**h** has been found to be further stabilized by the presence of a thermodynamically favorable six-membered intramolecular hydrogen bonding interactions between the carbonyl oxygen and the aryl-NH. Two compounds, namely, 1-(2-amino-5-styrylphenyl)ethan-1-one (**2a**) and 1-(2-amino-5-styryl)phenyl)-3-(4-fluorophenyl)prop-2-en-1-one (**3e**) exhibited significant inhibitory effect against both carbohydrate hydrolyzing enzymes (α-glucosidase and α-amylase) and antioxidant activity. These compounds have potential to suppress carbohydrate digestion, in turn, delay glucose uptake to lead to reduced blood sugar levels. Their antioxidant activity, on the other hand, make them suitable candidates to delay, inhibit, or prevent the oxidative damage by scavenging free radicals. Their safety profile was evaluated in vitro against the Vero and A549 cells, and the preliminary results showed no effect on the viability of both normal and cancerous cells after 24 h. These compounds will probably inhibit the activities of α-glucosidase and α-amylase, and also suppress some of the inflammatory effects mediated by free radicals with minimal or no cytotoxic effects on normal cells. Further cellular-based in vitro and in vivo studies including bioavailability and cell permeability would help to clarify the mechanism of action of these compounds in the body. Structural modifications of these stilbene-chalcone hybrids involving varying substitution pattern on the styryl and/or chalcone scaffolds can further enhance effectiveness against enzyme activity resulting in increased antihyperglycemic activity.

## Figures and Tables

**Table 1:** Designation and substitution of aryl groups for **3a**–**h**.

Ar	2a–d	3a–d	3e–h
C_6_H_5_-	**2a**	3-F (**3a**)	4-F (**3e**)
4-FC_6_H_4_-	**2b**	3-F (**3b**)	4-F (**3f**)
4-ClC_6_H_4_-	**2c**	3-F (**3c**)	4-F (**3g**)
4-MeOC_6_H_4_-	**2d**	3-F (**3d**)	4-F (**3h**)

**Table 2:** α-Glucosidase and α-amylase inhibition, and antioxidant activity of **2a**–**d** and **3a**–**h**.

Compound	(IC_50_ (SD) µM)			
	α-Glucosidase	α-Amylase	DPPH	NO
**2a**	5.4 ± 0.10	2.3 ± 0.20	7.1 ± 0.32	8.3 ± 0.22
**2b**	12.7 ± 0.16	19.3 ± 0.42	15.8 ± 0.21	7.2 ± 0.40
**2c**	15.9 ± 0.34	5.8 ± 0.51	10.4 ± 0.15	11.6 ± 0.43
**2d**	30.7 ± 0.42	9.0 ± 0.31	9.3 ± 0.23	25.9 ± 0.61
**3a**	17.8 ± 0.32	10.7 ± 0.21	21.6 ± 0.32	18.9 ± 0.54
**3b**	6.1 ± 0.24	15.6 ± 0.60	6.6 ± 0.33	5.3 ± 0.34
**3c**	12.6 ± 0.31	2.4 ± 0.10	15.3 ± 0.10	10.6 ± 0.30
**3d**	9.4 ± 0.50	7.0 ± 0.45	3.9 ± 0.21	7.9 ± 0.28
**3e**	5.1 ± 0.61	1.6 ± 0.52	4.2 ± 0.43	20.5 ± 0.46
**3f**	6.9 ± 0.37	9.5 ± 0.41	18.5 ± 0.37	8.8 ± 0.33
**3g**	19.2 ± 0.47	1.7 ± 0.25	5.2 ± 0.53	15.1 ± 0.19
**3h**	10.5 ± 0.18	7.6 ± 0.20	25.8 ± 0.43	20.2 ± 0.23
Acarbose	0.95 ± 0.28	1.03 ± 0.05	-	-
α-Amylase inhibitor	-	0.31 ± 0.05	-	-
Ascorbic acid	-	-	4.2 ± 0.27	6.14 ± 0.21

IC_50_ values (μM) were calculated from log dose inhibition curves and are expressed as means ± standard deviation (SD) of three independent experiments.

**Table 3:** The predicted binding free energy values of **2a**–**d** and **3a**–**h** obtained from docking simulation of the test compounds against α-glucosidase and α-amylase.

Compound	Binding Free Energy (kcal/mol)	
	α-Glucosidase	α-Amylase
**2a**	−7.78	−7.06
**2b**	−6.81	−6.33
**2c**	−7.63	−7.03
**2d**	−7.44	−6.43
**3a**	−6.70	−8.40
**3b**	−6.32	−8.34
**3c**	−6.75	−8.50
**3d**	−6.71	−9.31
**3e**	−7.30	−9.37
**3f**	−6.45	−8.32
**3g**	−7.87	−9.66
**3h**	−6.71	−6.96

**Table 4:** Pharmacokinetics properties predictions of compounds **2a**, **3e** and **3g**.

	Compound		
Property	2a	3e	3g
miLogP	3.81	5.94	6.62
Topological polar surface area; TPSA (Å)	43.09	43.06	43.09
Absorption (%); 109-(0.345 × TPSA)	94.13	94.14	94.13
Number of atom	18	26	27
Molecular weight	237.30	343.40	377.85
Molecular volume	229.70	316.90	330.43
Hydrogen bond acceptor	2	2	2
Hydrogen bond donor	2	2	2
Rotatable bonds	3	5	5
Lipinski’s violation	0	1	1

**Table 5:** The cytotoxicity of compounds **2a** and **3e** on Vero and A549 cell lines.

	Cytotoxicity, IC_50_ (µM)	
Compound	Vero Cells	A549 Cells
**2a**	164.56 ± 0.59	65.70 ± 0.14
**3e**	220.0 ± 0.57	204.00 ± 0.55
Doxorubicin	0.66 ± 0.12	1.14 ± 0.23

## Data Availability

The CIF files containing complete information on **2a** (CCDC 2053923) and **3f** (CCDC 2035809), and are freely available upon request from the following website: www.ccdc.cam.ac.uk/datarequest/cif, accessed on 4 April 2021 or by contacting the Cambridge Crystallographic Data Centre, 12, Union Road, Cambridge CB2 1EZ, UK; fax:+44 1223 336033; email: deposit@ccdc.cam.ac.uk.

## Supplementary Materials

Copies of ^1^H- and ^13^C NMR spectra (Figure S1) and FT-IR spectra (Figure S2) of the text compounds, and interactions compounds **2c, 2d**, **3a**, **3c**, **3d**, **3f** and **3h** with α-glucosidase (Figure S3) and α-amylase (Figure S4).

## Sample Availability

Samples of the compounds are available from the authors.
