# Supplementary material for: Synthesis, Structure, Carbohydrate Enzyme Inhibition, Antioxidant Activity, In Silico Drug-Receptor Interactions and Drug-Like Profiling of the 5-Styryl-2-Aminochalcone Hybrids
Source: Molecules. 2021 May 4;26(9):2692. doi: 10.3390/molecules26092692 (PMC8125089; doi:10.3390/molecules26092692)
Supplement: Supplementary file 1 [file molecules-26-02692-s001.zip › molecules-1181576-SI.pdf]

Supplementary Information

Synthesis, Structure, Carbohydrate Enzyme Inhibition, Antioxidant Activity, In Silico Drug-Receptor Interactions and Drug-Like Profiling of the 5-Styryl-2-Aminochalcone Hybrids

Malose J. Mphahlele [1,*], Emmanuel Ndubuisi Agbo [1] and Yee Siew Choong [2,*]

Figure S1: Copies of ^{1}H- and ^{13}C NMR spectra of the test compounds
Figure S2: Copies of IR spectra of the test compounds
Figure S3. The interactions of compounds **2c, 2d**, **3a**, **3c**, **3d**, **3f** and **3h** against α-glucosidase.
Figure S4. The interactions of compounds **2c, 2d**, **3a**, **3c**, **3d**, **3f** and **3h** against α-amylase.

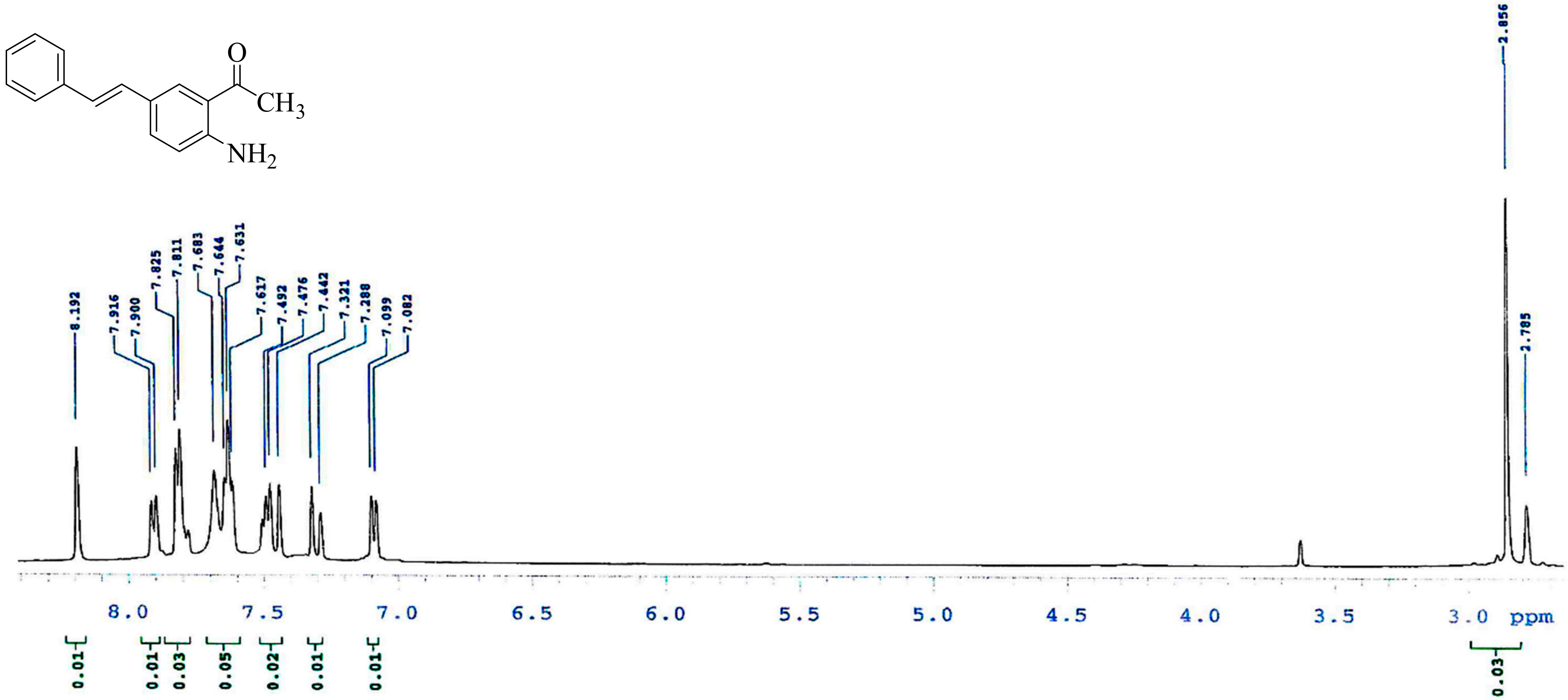

O
CH3
NH2
8.192
7.916
7.900
7.825
7.811
7.683
7.644
7.631
7.617
7.492
7.476
7.442
7.321
7.288
7.099
7.082
2.856
2.785
8.0
7.5
7.0
6.5
6.0
5.5
5.0
4.5
4.0
3.5
3.0
ppm
0.01
0.01
0.03
0.05
0.02
0.01
0.01
0.03

(a)

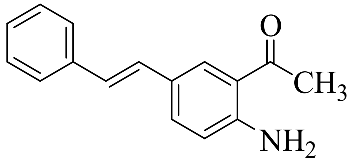

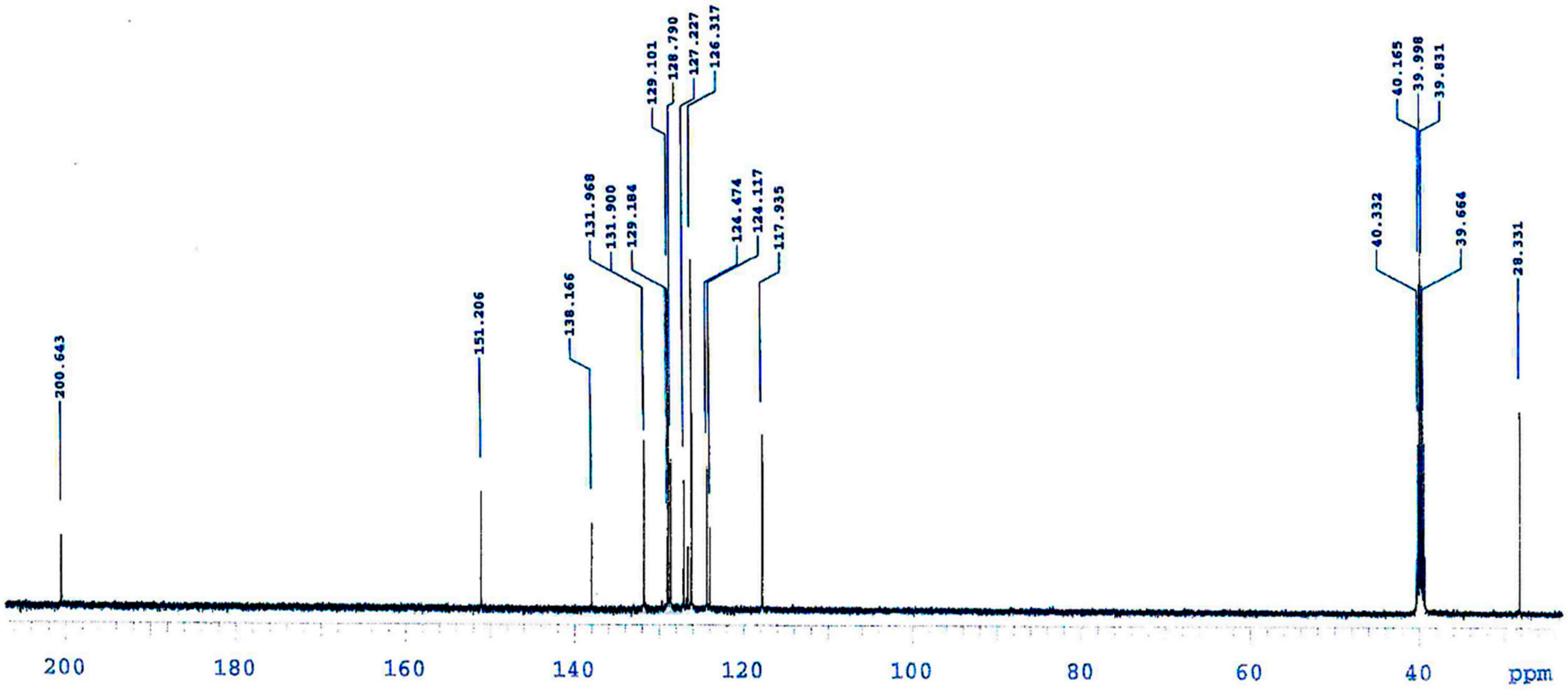

(**b**)

Figure S1.1: ^{1}H- and ^{13}C-NMR spectra of **2a** in DMSO-d_6 at 500 MHz (**a**) and 125 MHz (**b**), respectively.

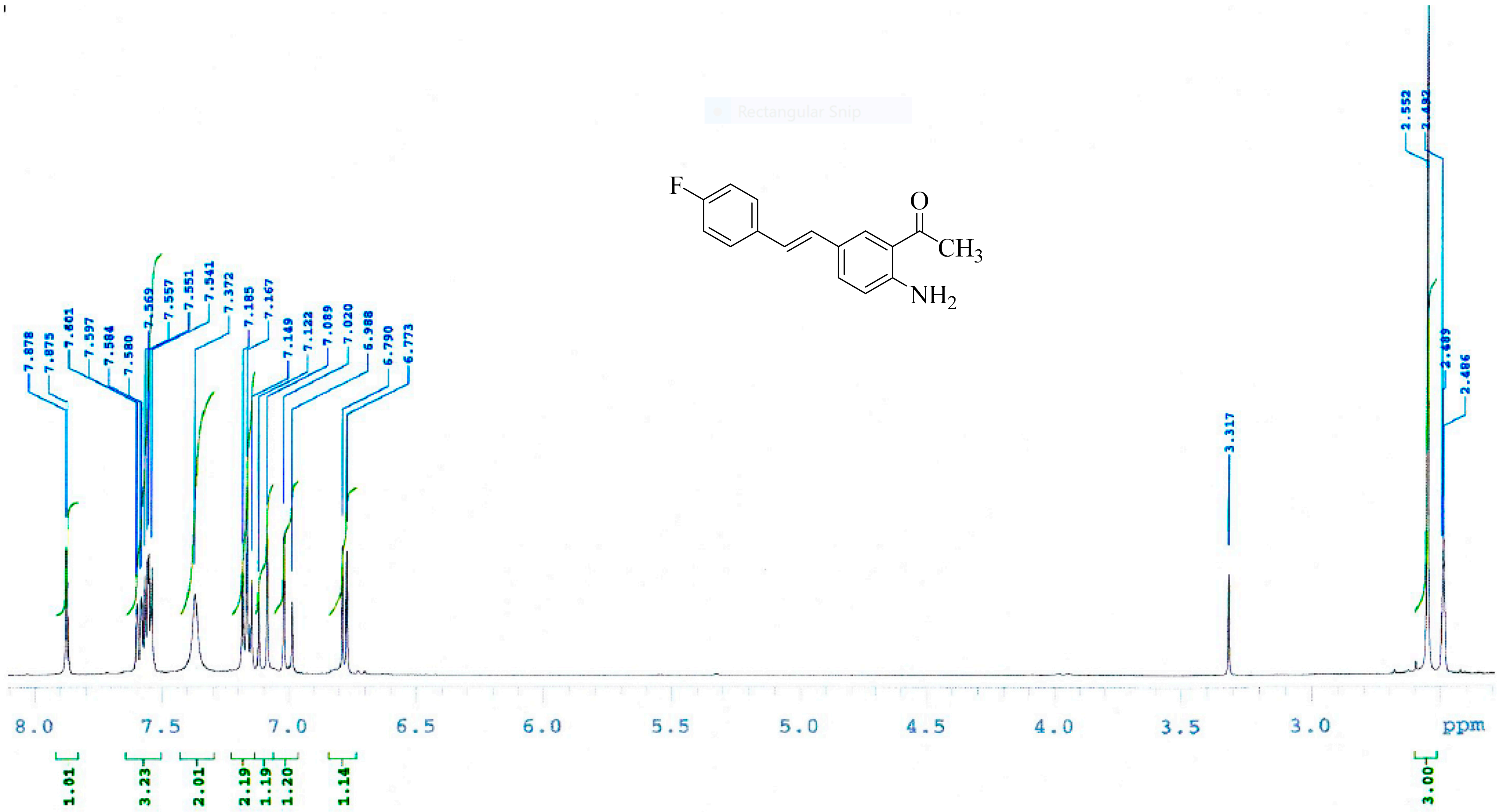
F
O
CH_3
NH_2
7.878
7.875
7.601
7.597
7.584
7.580
7.569
7.557
7.551
7.541
7.372
7.185
7.167
7.149
7.122
7.089
7.020
6.988
6.790
6.773
3.317
2.552
2.489
2.486
8.0
7.5
7.0
6.5
6.0
5.5
5.0
4.5
4.0
3.5
3.0
ppm
1.01
3.23
2.01
2.19
1.19
1.20
1.14
3.00

(**a**)

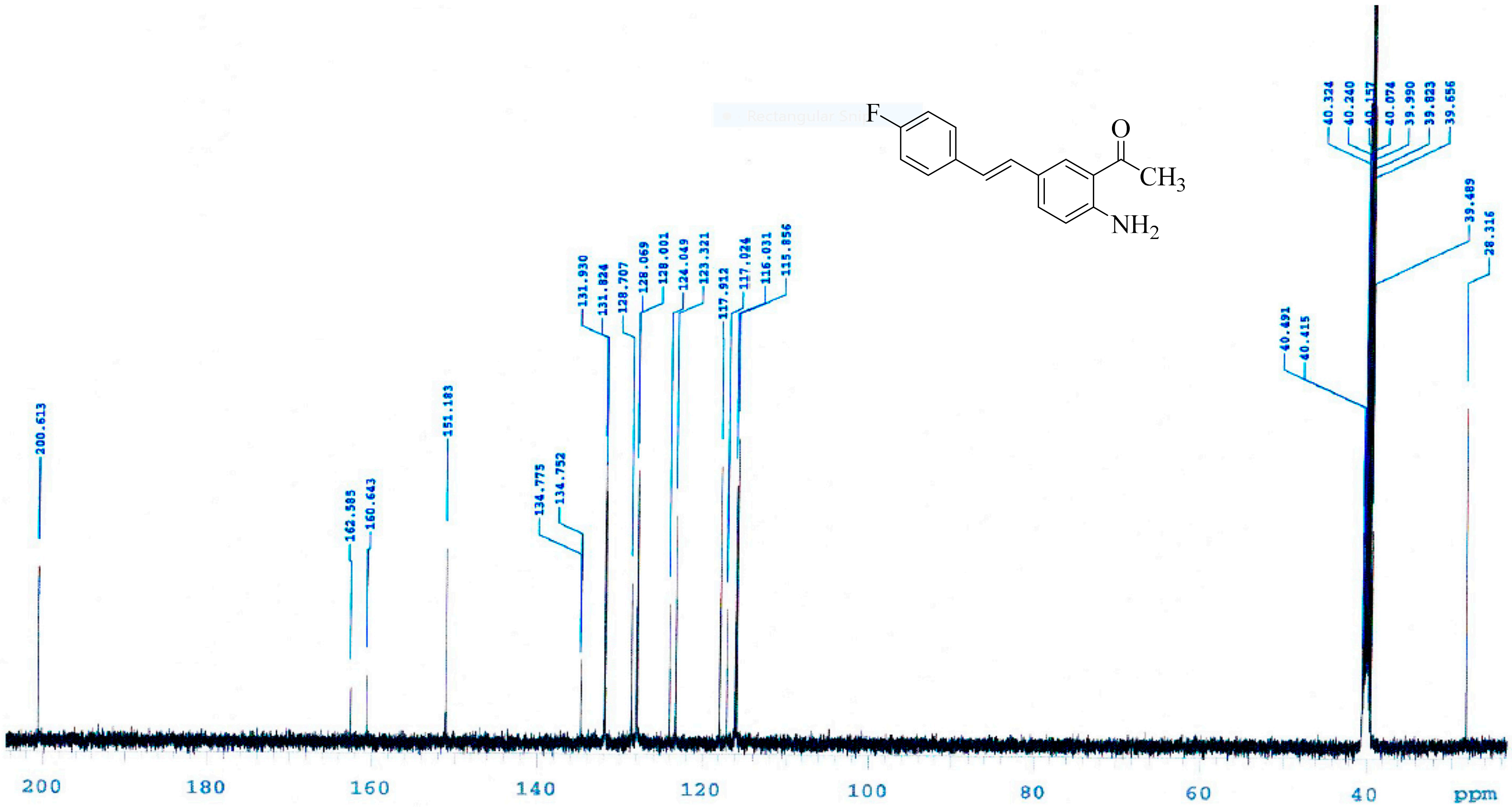

(**b**)

Figure S1.2: ^{1}H- and ^{13}C-NMR spectra of **2b** in DMSO-d_6 at 500 MHz (a) and 125 MHz (b), respectively.

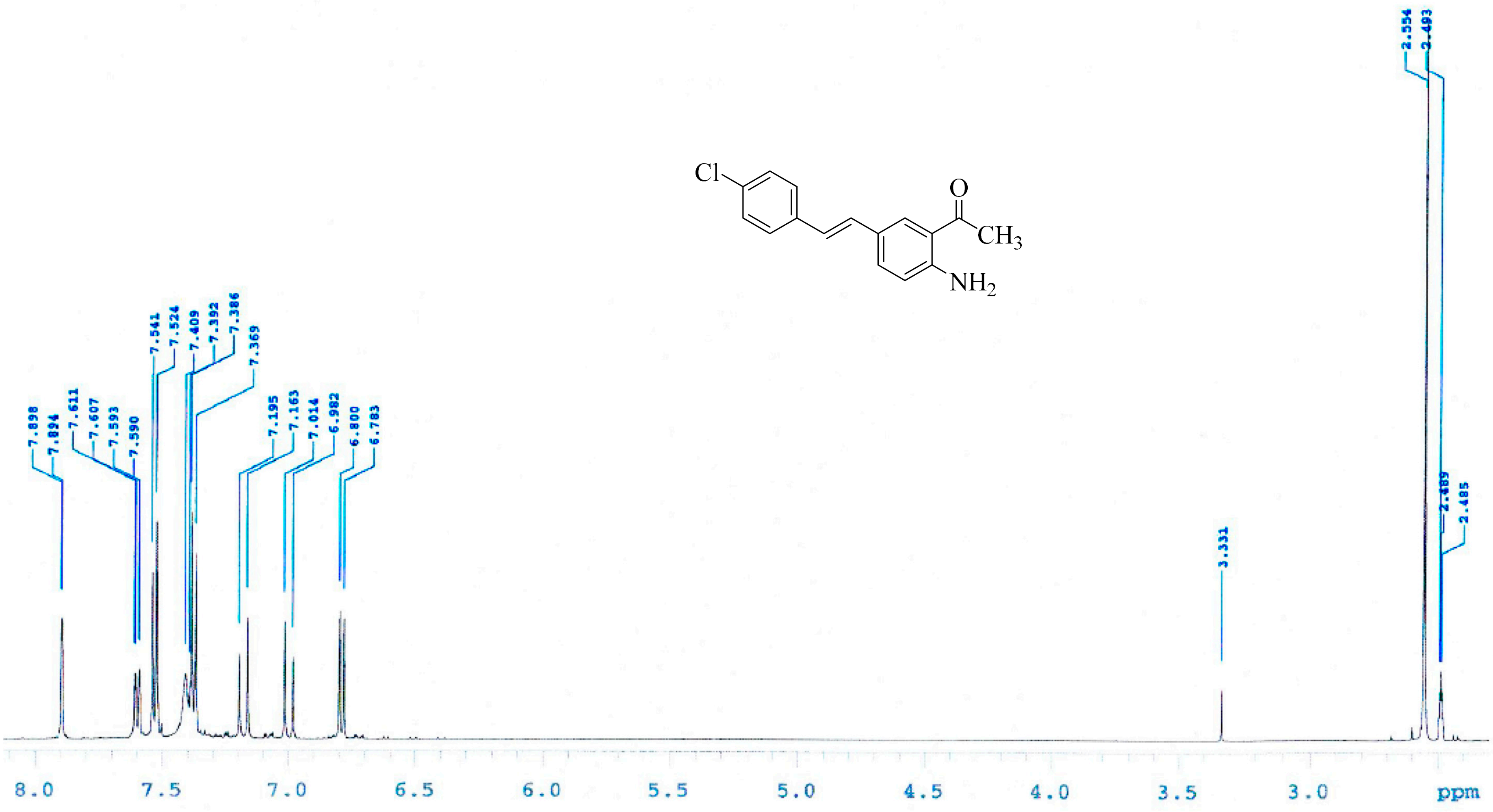
Cl
O
CH3
NH2
7.898
7.894
7.611
7.607
7.593
7.590
7.541
7.524
7.409
7.392
7.386
7.369
7.195
7.163
7.014
6.982
6.800
6.783
3.331
2.554
2.493
2.489
2.485
8.0
7.5
7.0
6.5
6.0
5.5
5.0
4.5
4.0
3.5
3.0
ppm

(a)

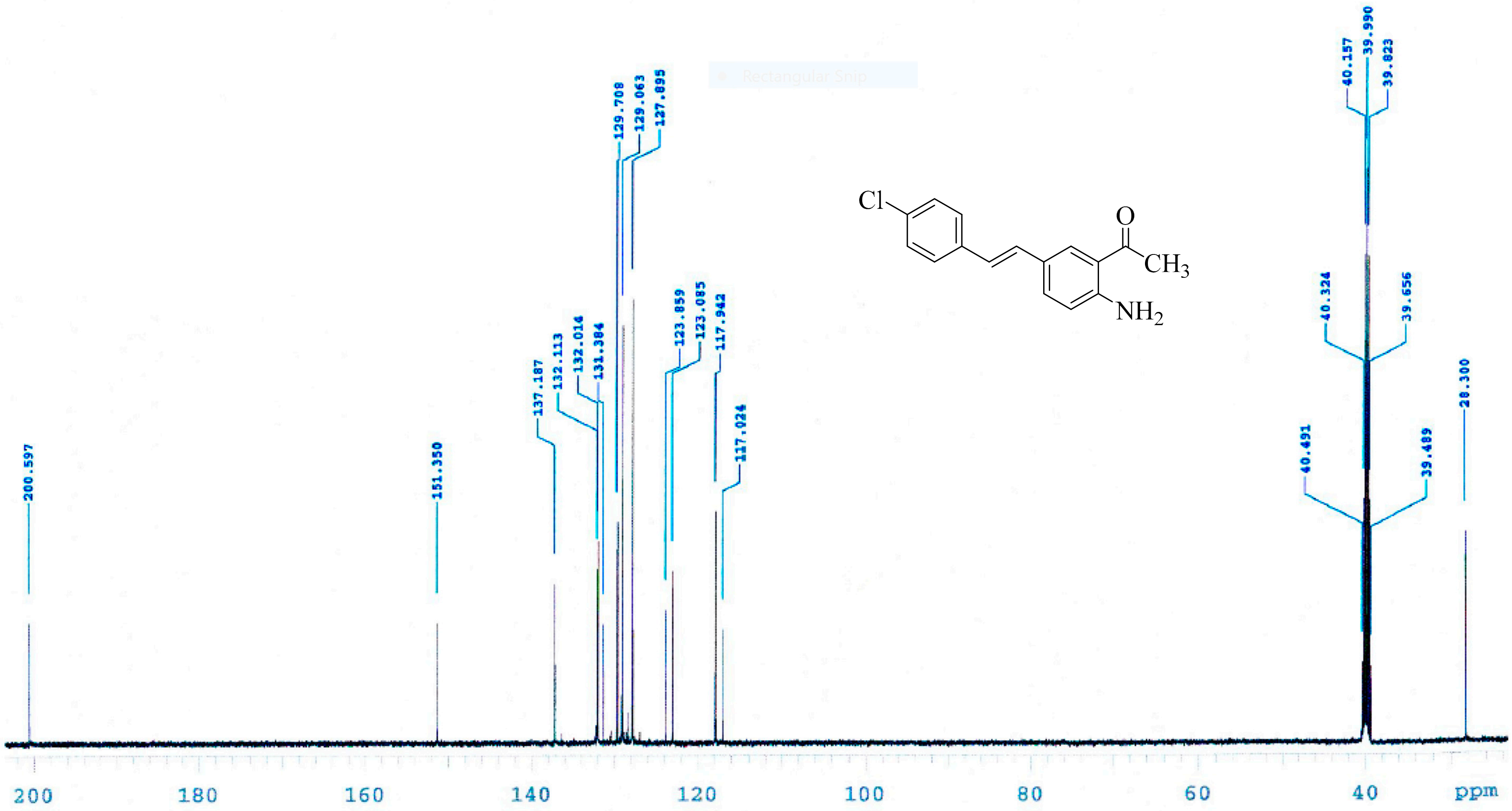

(**b**)

Figure S1.3: ^{1}H- and ^{13}C-NMR spectra of **2c** in DMSO-d_6 at 500 MHz (**a**) and 125 MHz (**b**), respectively.

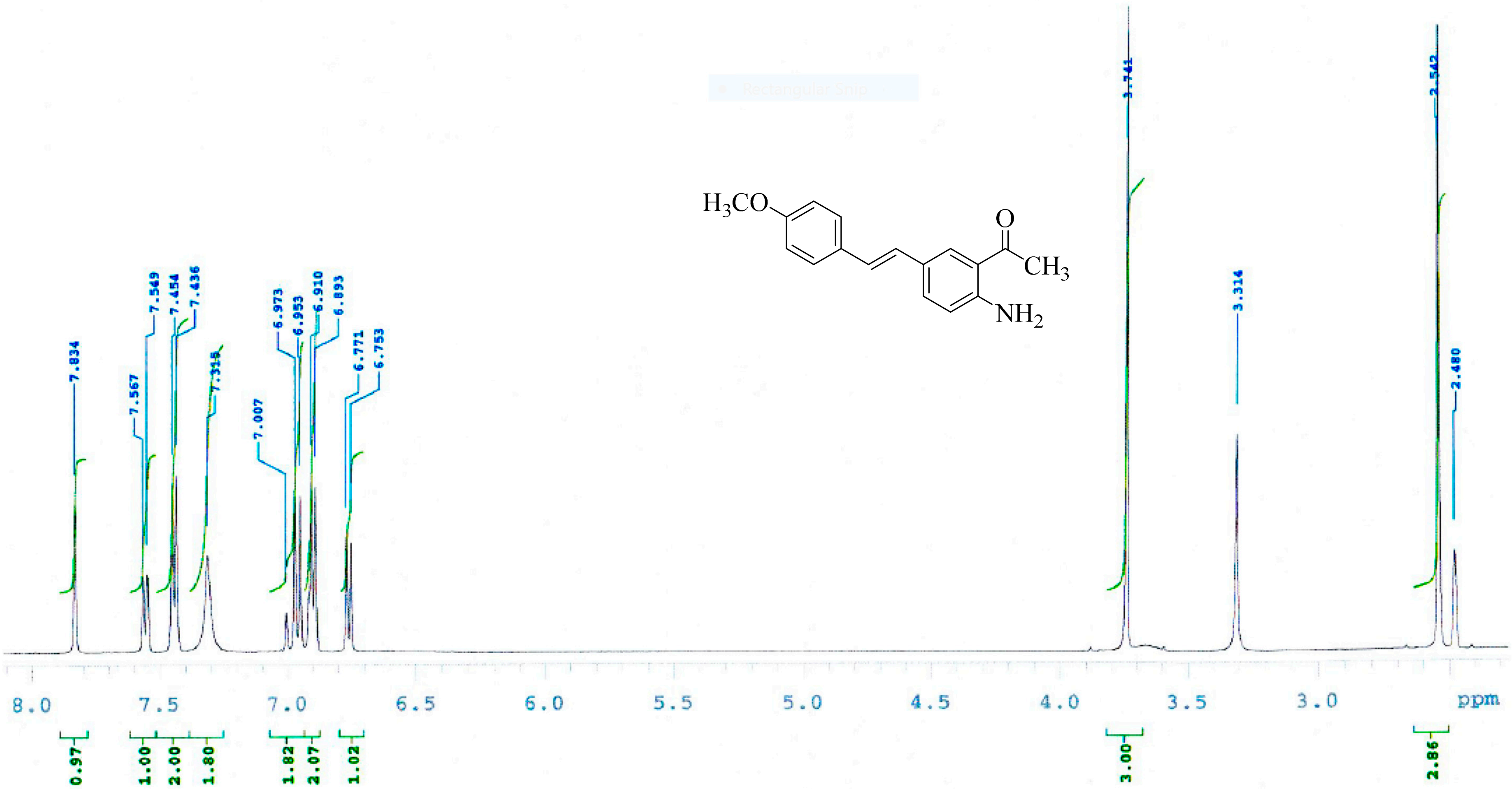
H3CO
O
CH3
NH2
7.834
7.567
7.549
7.454
7.436
7.315
7.007
6.973
6.953
6.910
6.893
6.771
6.753
3.741
3.314
2.542
2.480
8.0
7.5
7.0
6.5
6.0
5.5
5.0
4.5
4.0
3.5
3.0
ppm
0.97
1.00
2.00
1.80
1.82
2.07
1.02
3.00
2.86

(**a**)

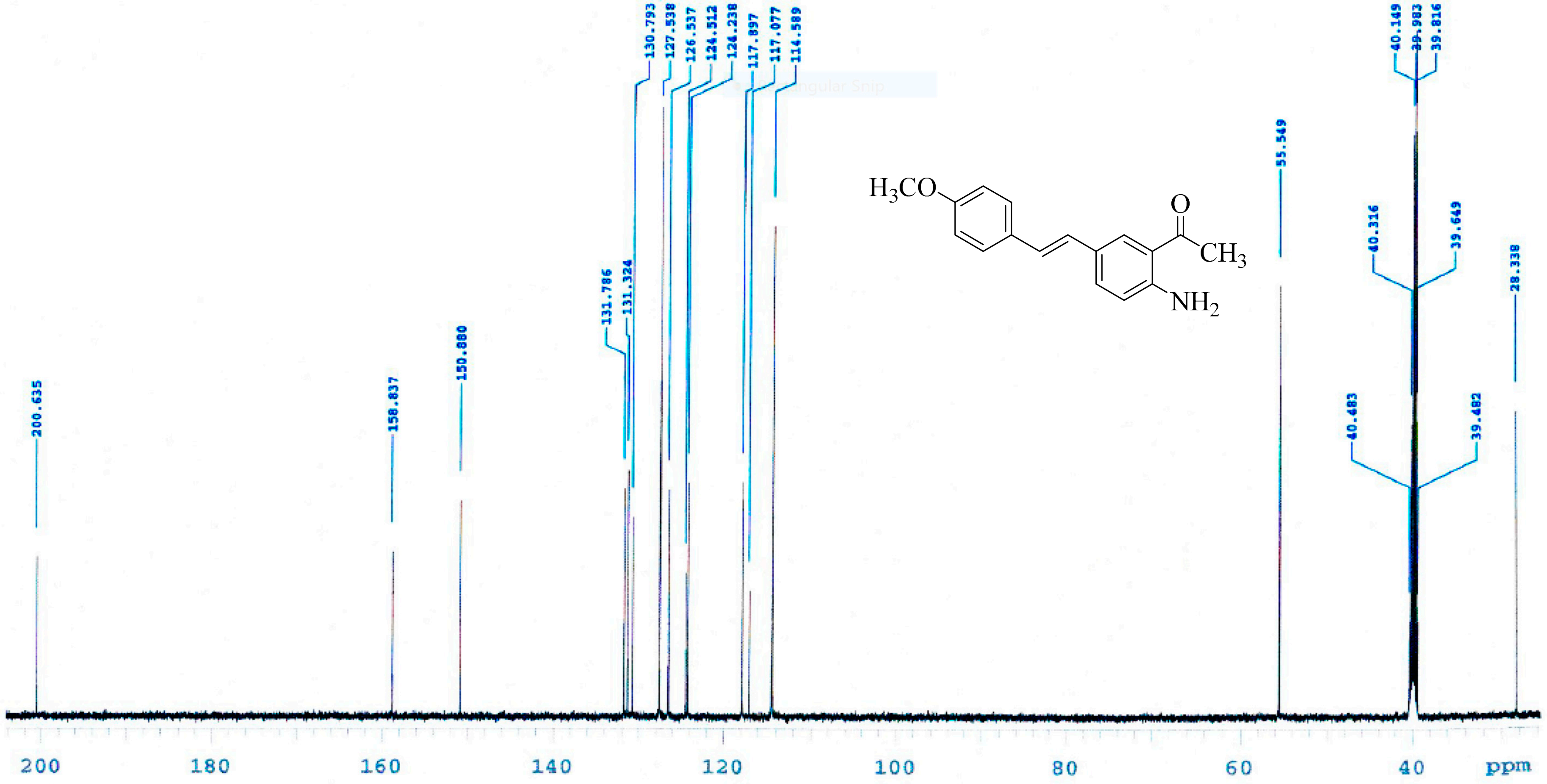

(**b**)

Figure S1.4: ^{1}H- and ^{13}C-NMR spectra of **2d** in DMSO-d_6 at 500 MHz (**a**) and 125 MHz (**b**), respectively.

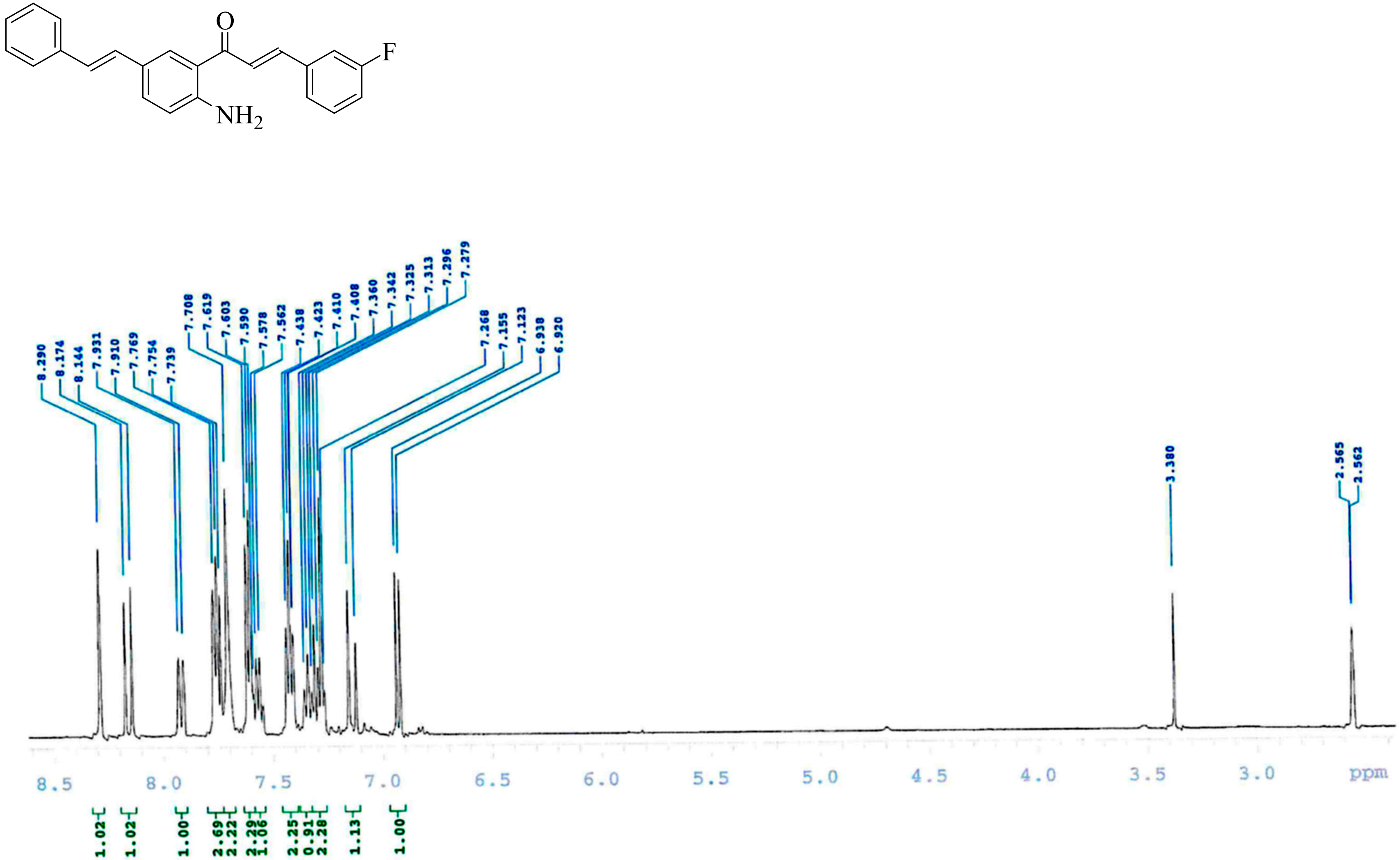
O
F
NH2
8.290
8.174
8.144
7.931
7.910
7.769
7.754
7.739
7.708
7.619
7.603
7.590
7.578
7.562
7.438
7.423
7.410
7.408
7.360
7.342
7.325
7.313
7.296
7.279
7.268
7.155
7.123
6.938
6.920
3.380
2.565
2.562
8.5
8.0
7.5
7.0
6.5
6.0
5.5
5.0
4.5
4.0
3.5
3.0
ppm
1.02
1.02
1.00
2.69
2.22
2.29
1.06
2.25
0.91
2.28
1.13
1.00

(**a**)

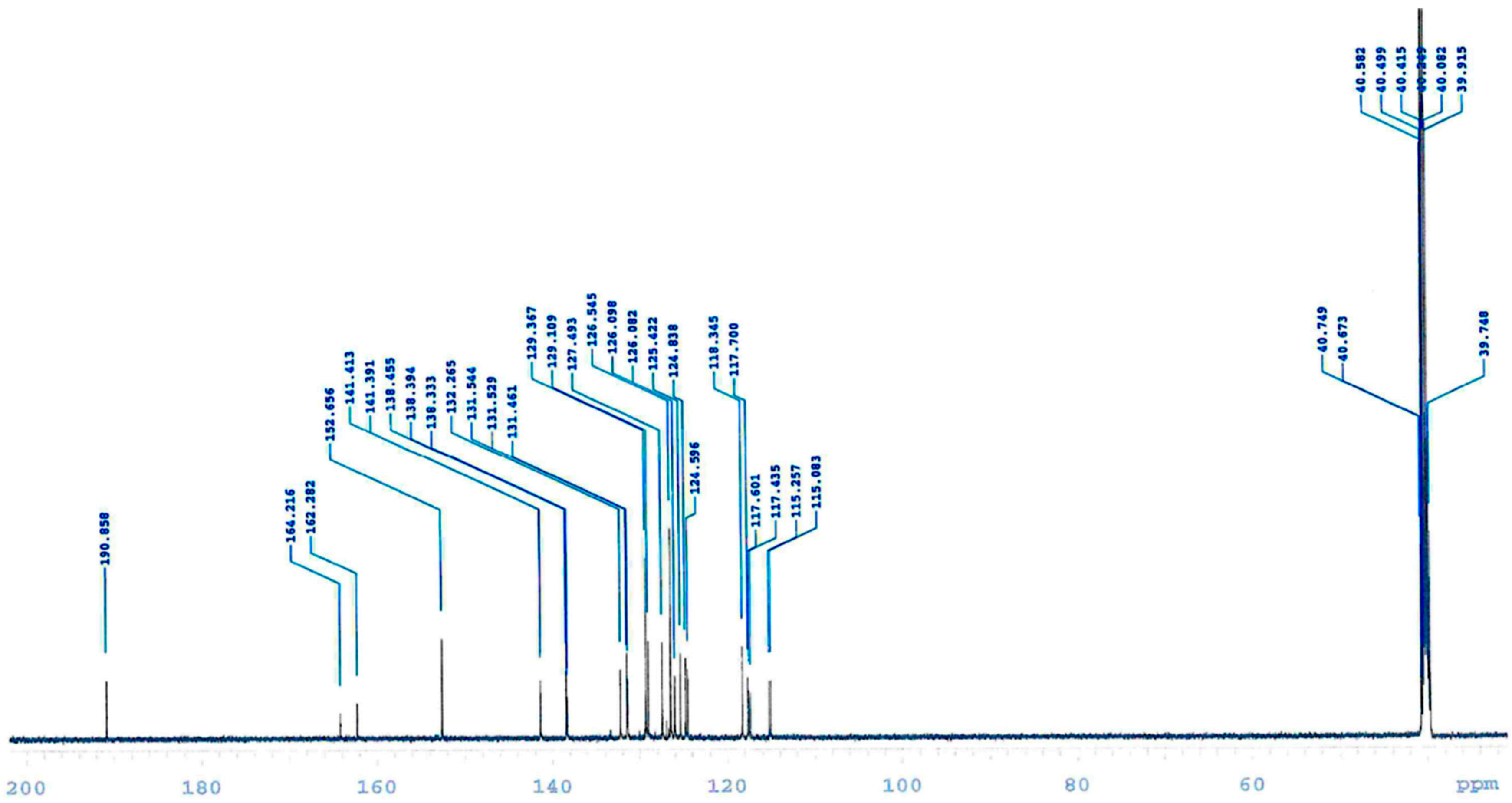

(**b**)

Figure S1.5: ^{1}H- and ^{13}C-NMR spectra of **3a** in DMSO-d_6 at 500 MHz (**a**) and 125 MHz (**b**), respectively.

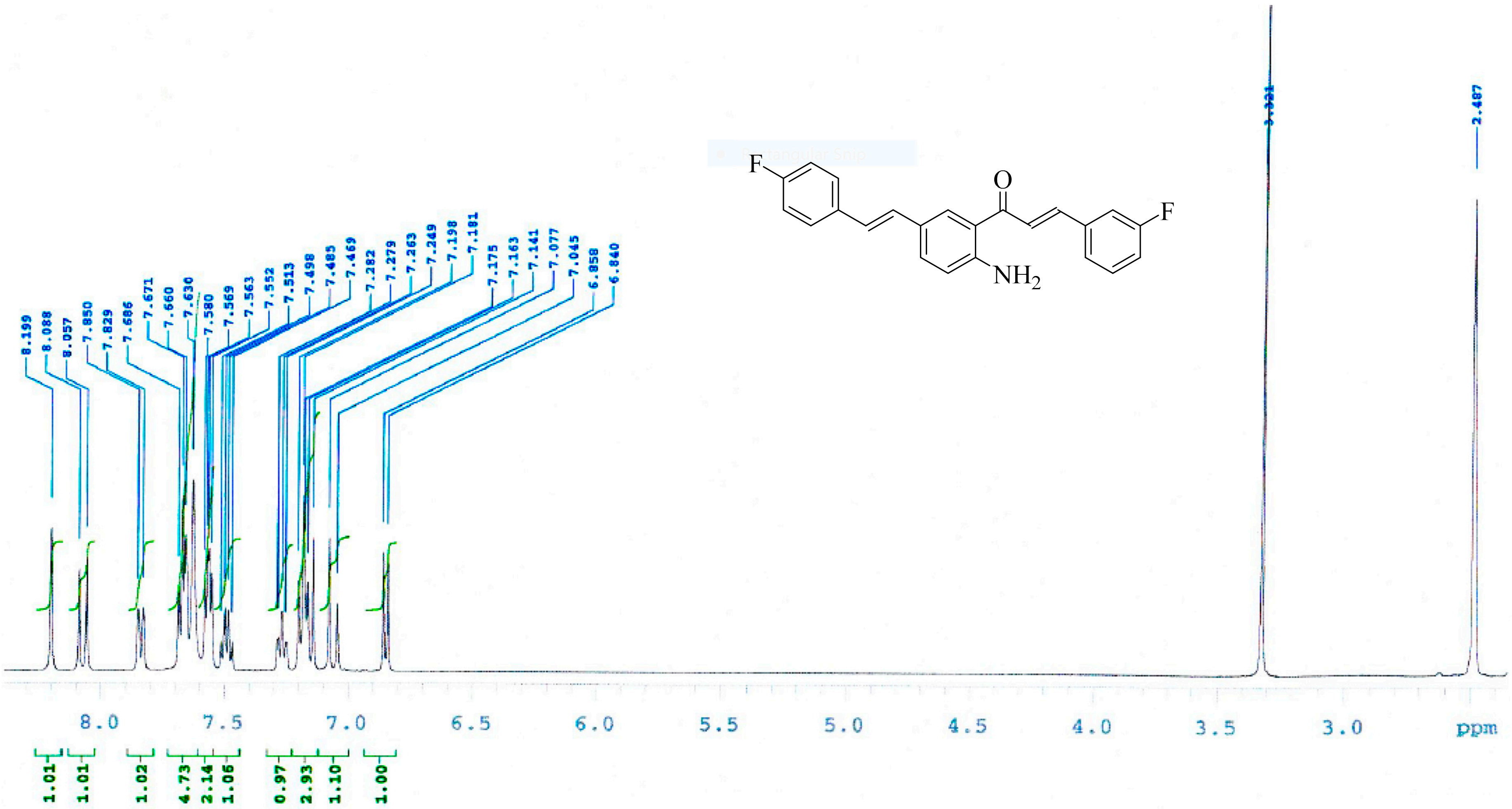
F
O
F
NH_2
8.199
8.088
8.057
7.850
7.829
7.686
7.671
7.660
7.630
7.580
7.569
7.563
7.552
7.513
7.498
7.485
7.469
7.282
7.279
7.263
7.249
7.198
7.181
7.175
7.163
7.141
7.077
7.045
6.858
6.840
2.487
8.0
7.5
7.0
6.5
6.0
5.5
5.0
4.5
4.0
3.5
3.0
ppm
1.01
1.01
1.02
4.73
2.14
1.06
0.97
2.93
1.10
1.00

(**a**)

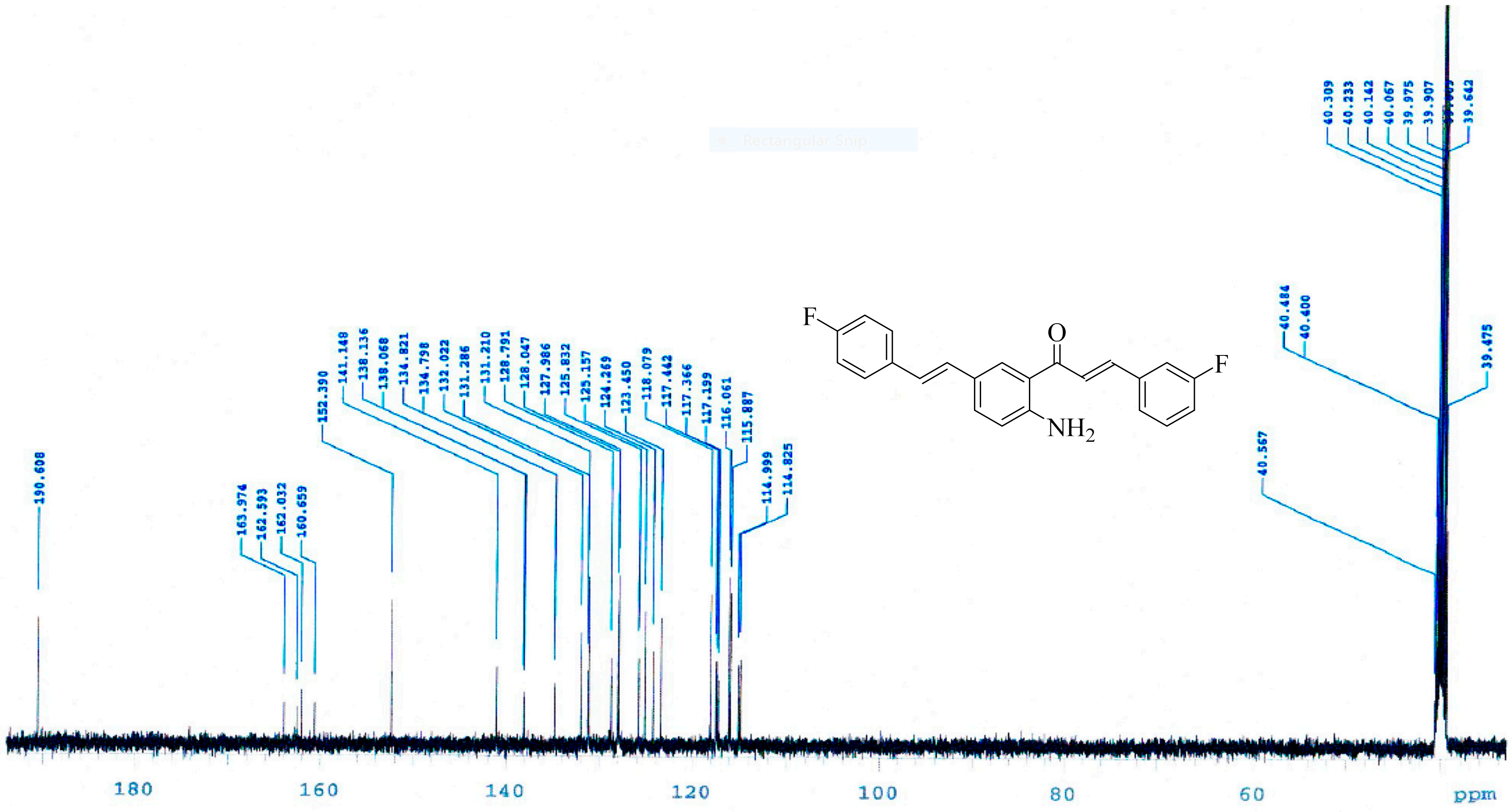

(**b**)

Figure S1.6: ^{1}H- and ^{13}C-NMR spectra of **3b** in DMSO-d_6 at 500 MHz (**a**) and 125 MHz (**b**), respectively.

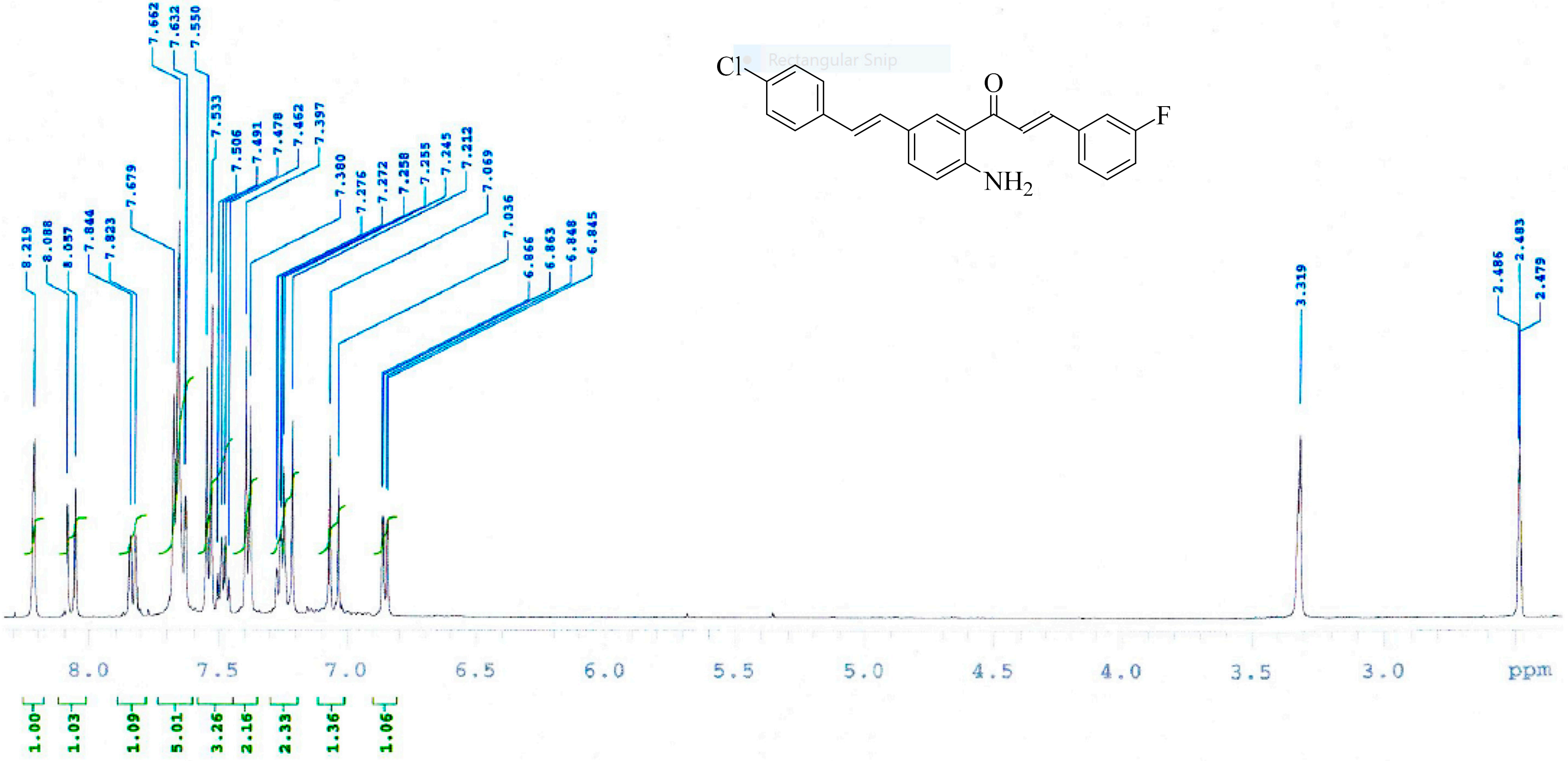
Cl
O
F
NH$_2$
Rectangular Snip
8.219
8.088
8.057
7.844
7.823
7.679
7.662
7.632
7.550
7.533
7.506
7.491
7.478
7.462
7.397
7.380
7.276
7.272
7.258
7.255
7.245
7.212
7.069
7.036
6.866
6.863
6.848
6.845
3.319
2.486
2.483
2.479
8.0
7.5
7.0
6.5
6.0
5.5
5.0
4.5
4.0
3.5
3.0
ppm
1.00
1.03
1.09
5.01
3.26
2.16
2.33
1.36
1.06

(**a**)

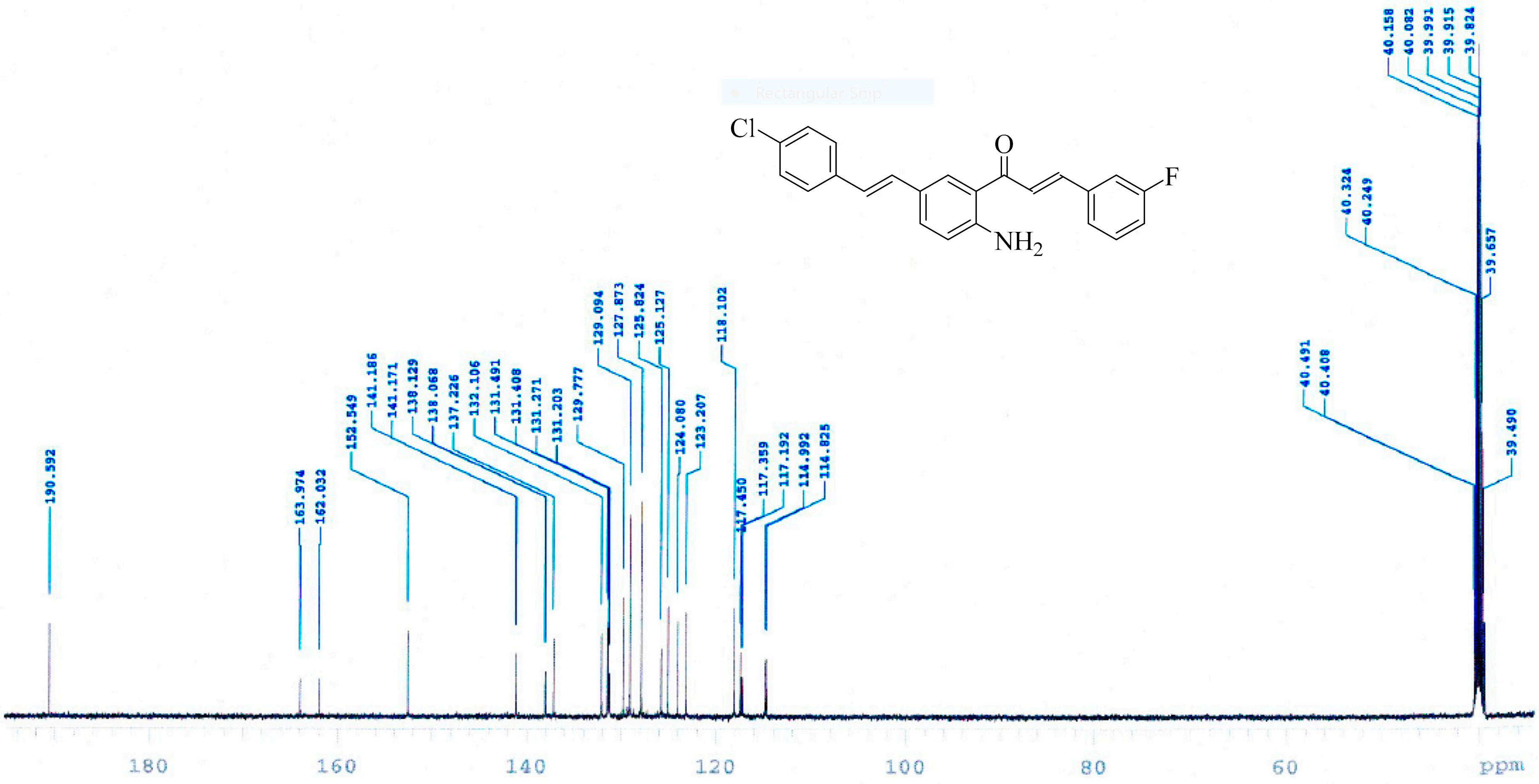

(**b**)

Figure S1.7: ^{1}H- and ^{13}C-NMR spectra of **3c** in DMSO-d_6 at 500 MHz (**a**) and 125 MHz (**b**), respectively.

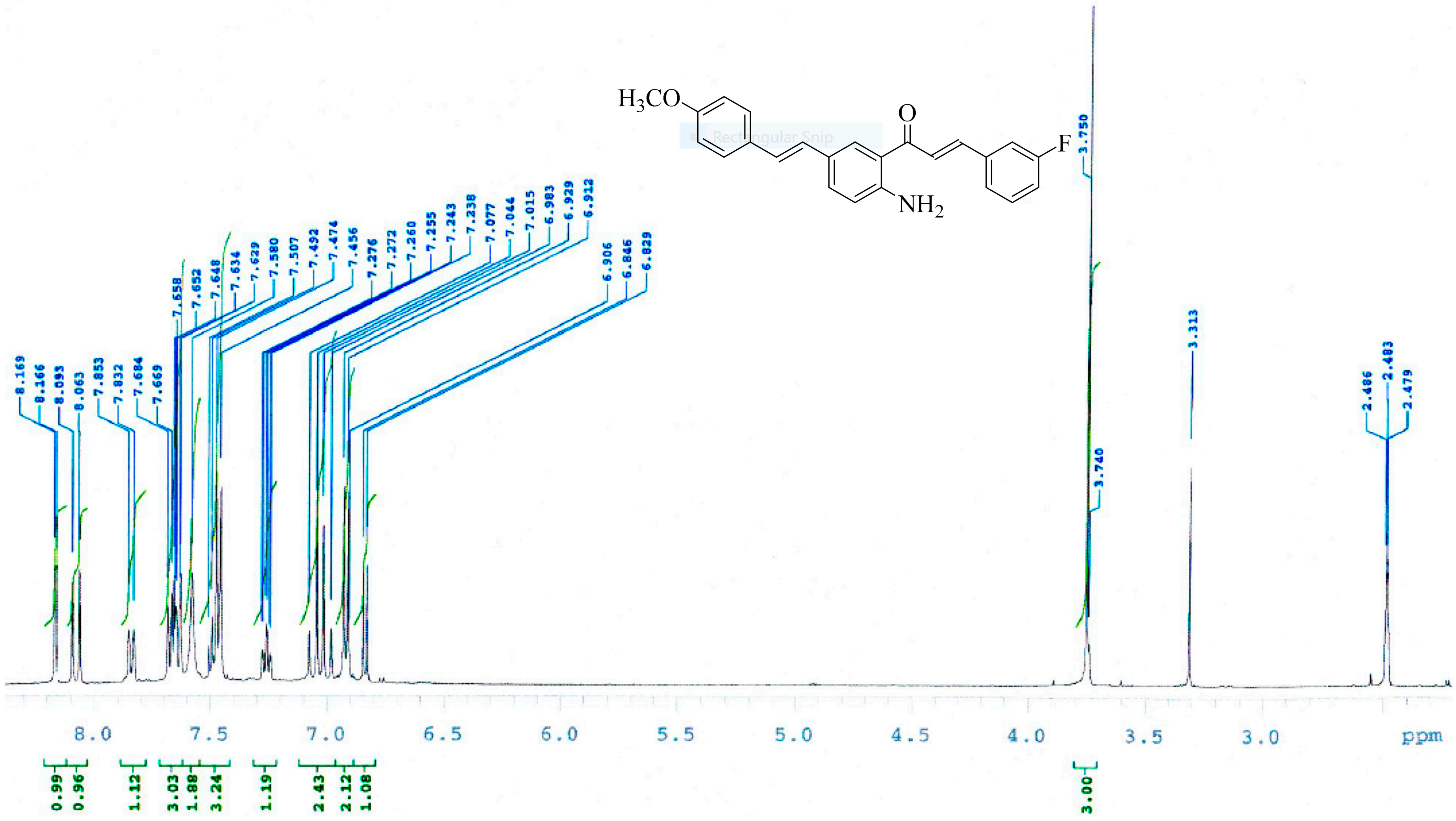

H_3CO
O
F
NH_2
8.169
8.166
8.093
8.063
7.853
7.832
7.684
7.669
7.658
7.652
7.648
7.634
7.629
7.580
7.507
7.492
7.474
7.456
7.276
7.272
7.260
7.255
7.243
7.238
7.077
7.044
7.015
6.983
6.929
6.912
6.906
6.846
6.829
3.750
3.740
3.313
2.486
2.483
2.479
8.0
7.5
7.0
6.5
6.0
5.5
5.0
4.5
4.0
3.5
3.0
ppm
0.99
0.96
1.12
3.03
1.88
3.24
1.19
2.43
2.12
1.08
3.00

(**a**)

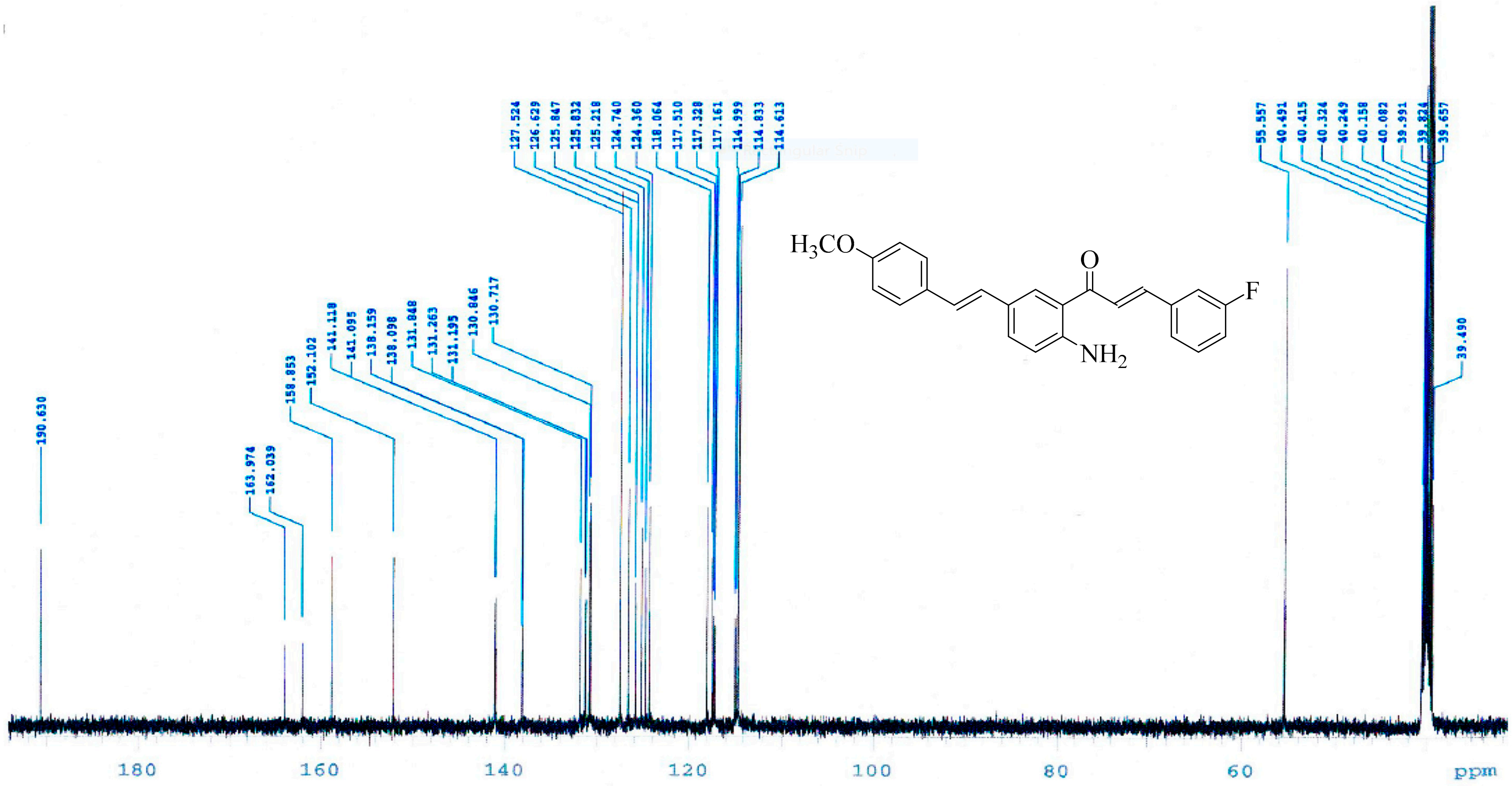

(**b**)

Figure S1.8: ^{1}H- and ^{13}C-NMR spectra of **3d** in DMSO-*d*6 at 500 MHz (**a**) and 125 MHz (**b**), respectively.

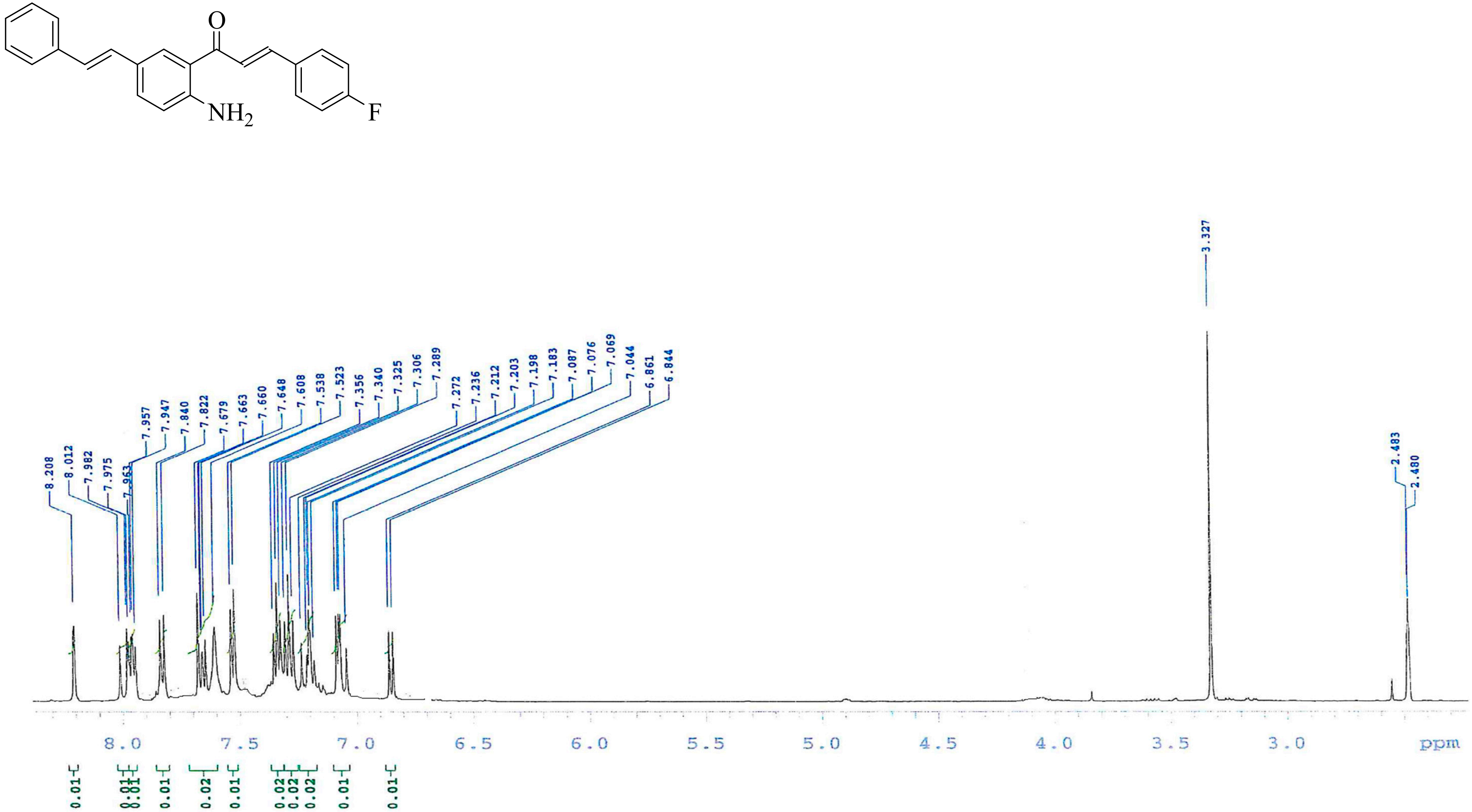
O
NH2
F
8.208
8.012
7.982
7.975
7.963
7.957
7.947
7.840
7.822
7.679
7.663
7.660
7.648
7.608
7.538
7.523
7.356
7.340
7.325
7.306
7.289
7.272
7.236
7.212
7.203
7.198
7.183
7.087
7.076
7.069
7.044
6.861
6.844
3.327
2.483
2.480
8.0
7.5
7.0
6.5
6.0
5.5
5.0
4.5
4.0
3.5
3.0
ppm

(**a**)

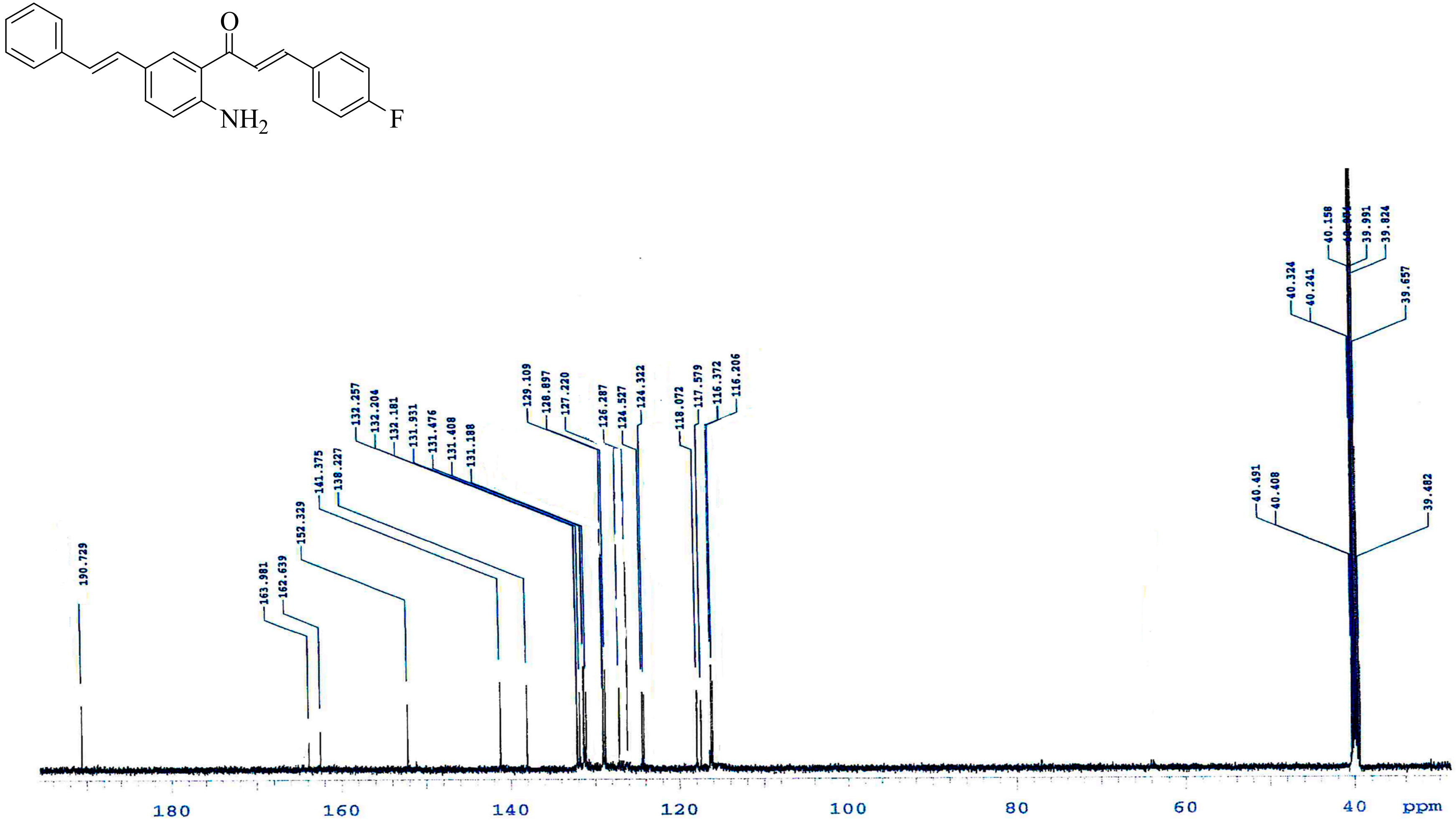

(**b**)

Figure S1.9: ^{1}H- and ^{13}C-NMR spectra of **3e** in DMSO-d_6 at 500 MHz (**a**) and 125 MHz (**b**), respectively.

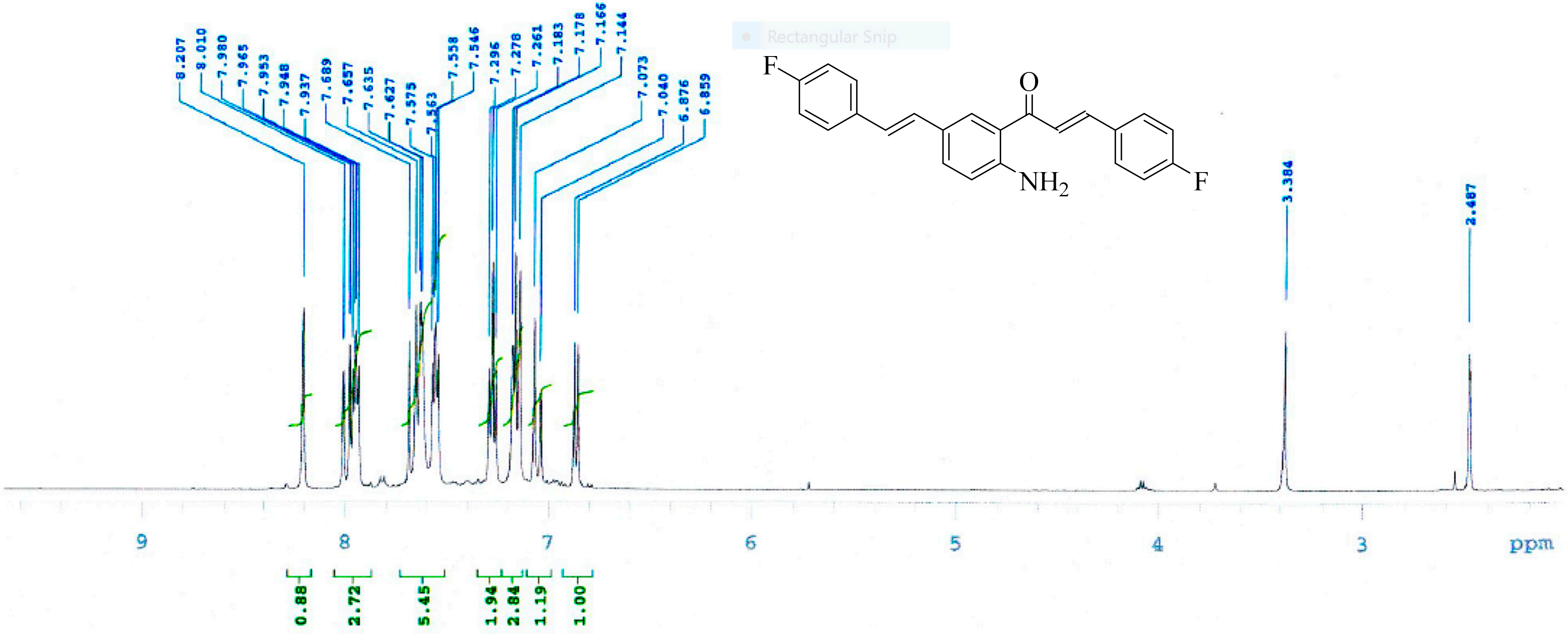
8.207
8.010
7.980
7.965
7.953
7.948
7.937
7.689
7.657
7.635
7.627
7.575
7.563
7.558
7.546
7.296
7.278
7.261
7.183
7.178
7.166
7.144
7.073
7.040
6.876
6.859
Rectangular Snip
F
O
NH2
F
3.384
2.487
9
8
7
6
5
4
3
ppm
0.88
2.72
5.45
1.94
2.84
1.19
1.00

(**a**)

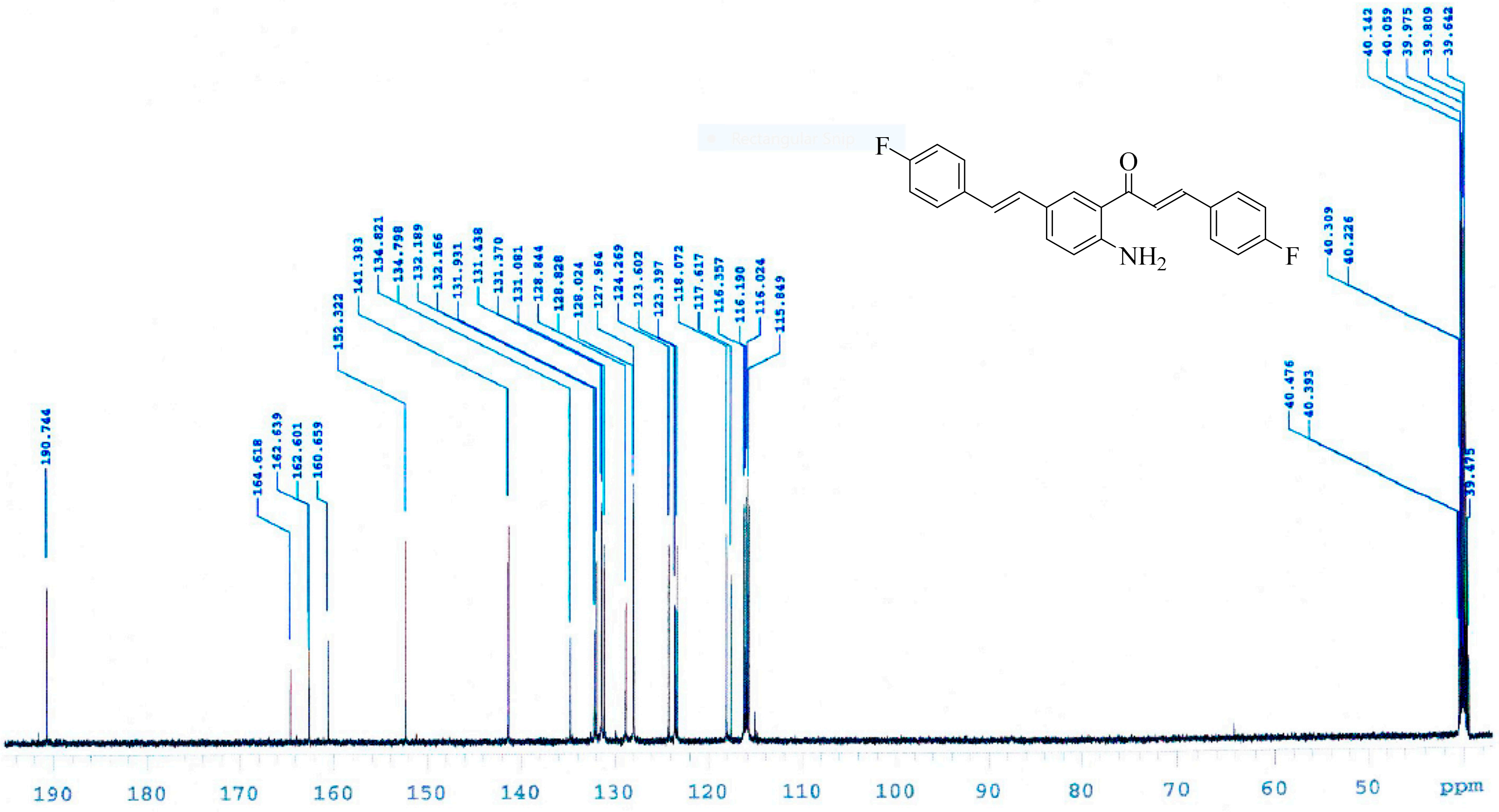

(**b**)

Figure S1.10: ^{1}H- and ^{13}C-NMR spectra of **3f** in DMSO-d_6 at 500 MHz (**a**) and 125 MHz (**b**), respectively.

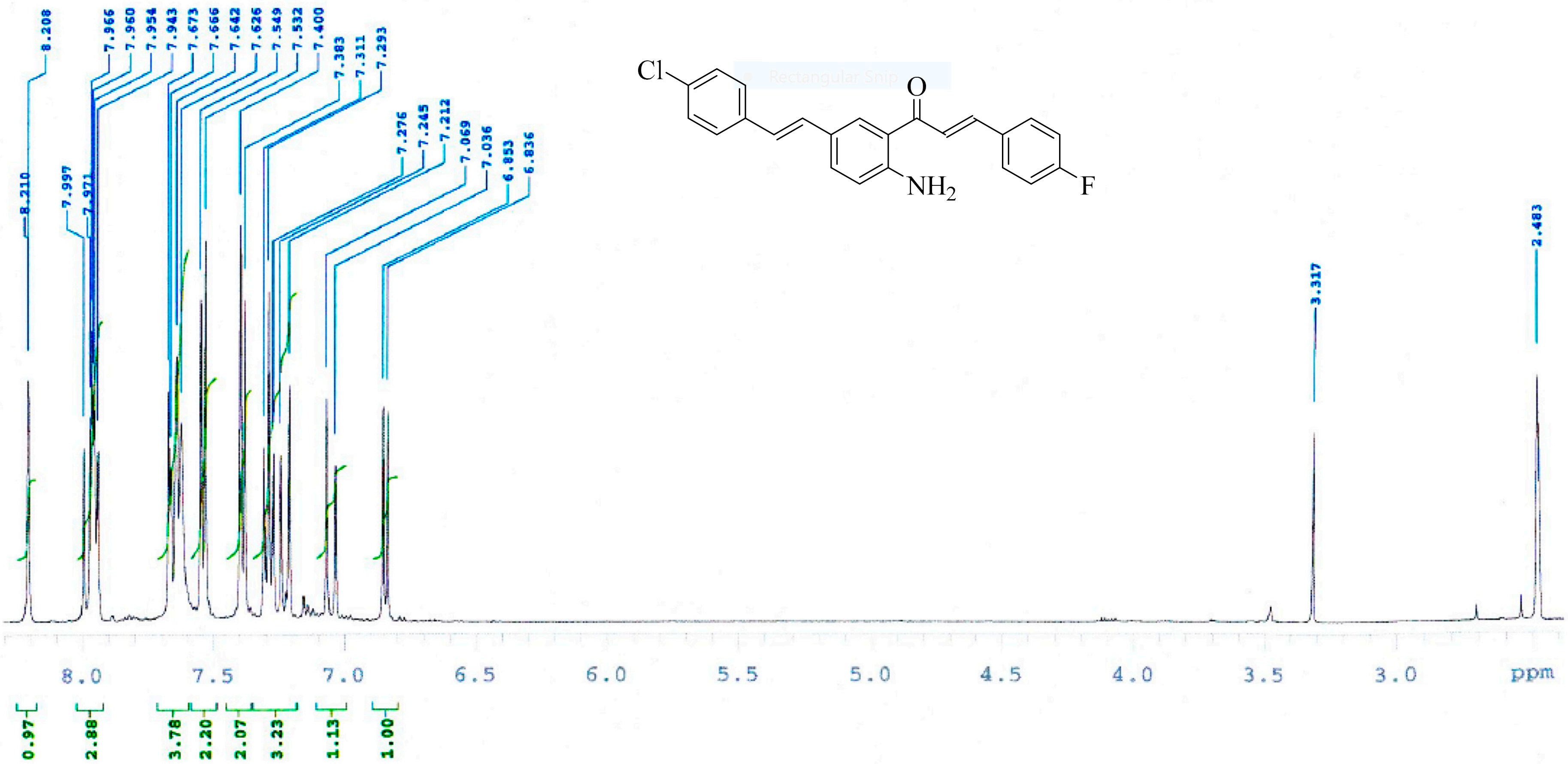
Cl
O
NH2
F
8.208
8.210
7.997
7.971
7.966
7.960
7.954
7.943
7.673
7.666
7.642
7.626
7.549
7.532
7.400
7.383
7.311
7.293
7.276
7.245
7.212
7.069
7.036
6.853
6.836
3.317
2.483
8.0
7.5
7.0
6.5
6.0
5.5
5.0
4.5
4.0
3.5
3.0
ppm
0.97
2.88
3.78
2.20
2.07
3.23
1.13
1.00

(**a**)

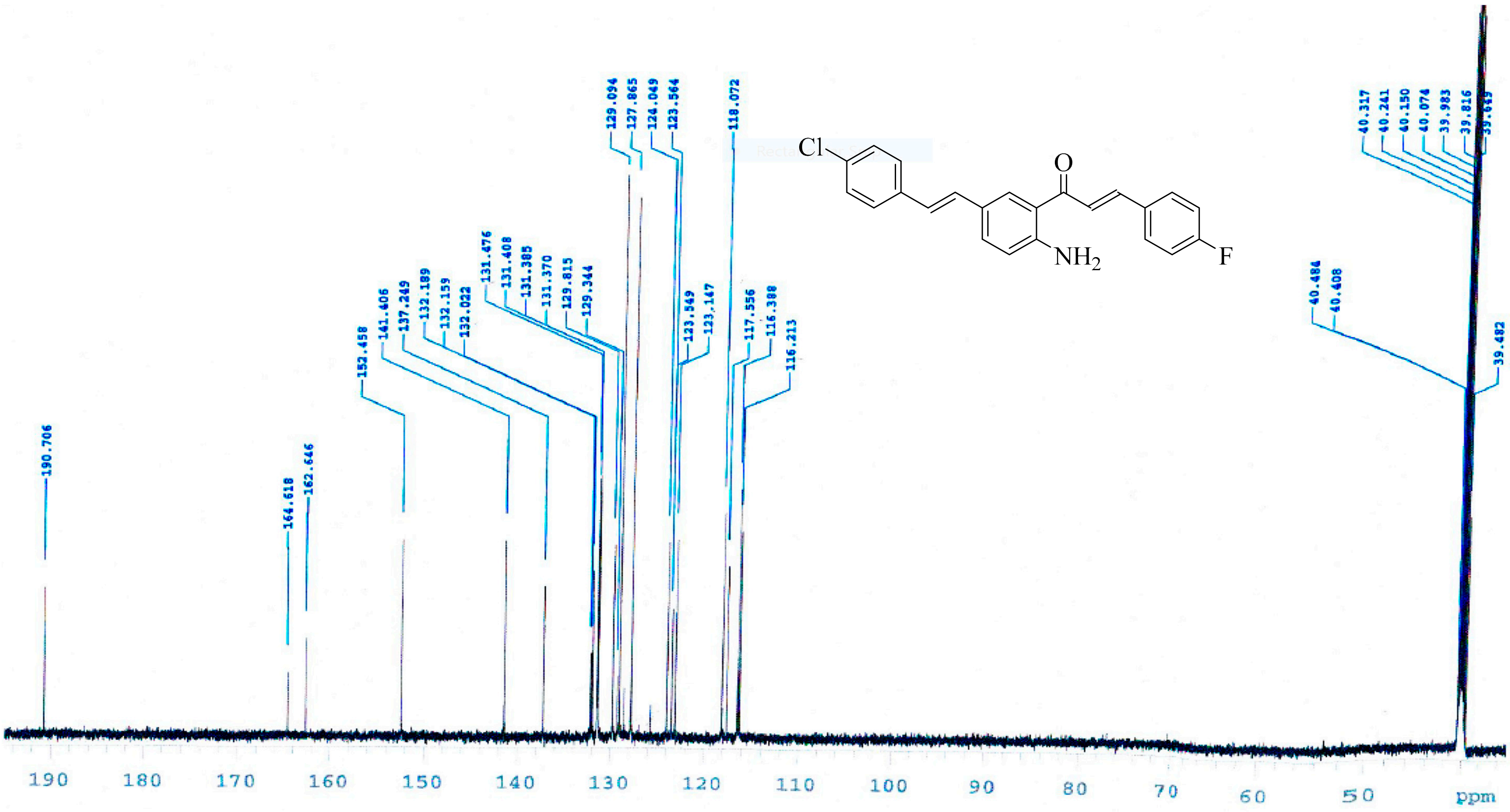

(**b**)

Figure S1.11: ^{1}H- and ^{13}C-NMR spectra of **3g** in DMSO-*d*$_6$ at 500 MHz (**a**) and 125 MHz (**b**), respectively.

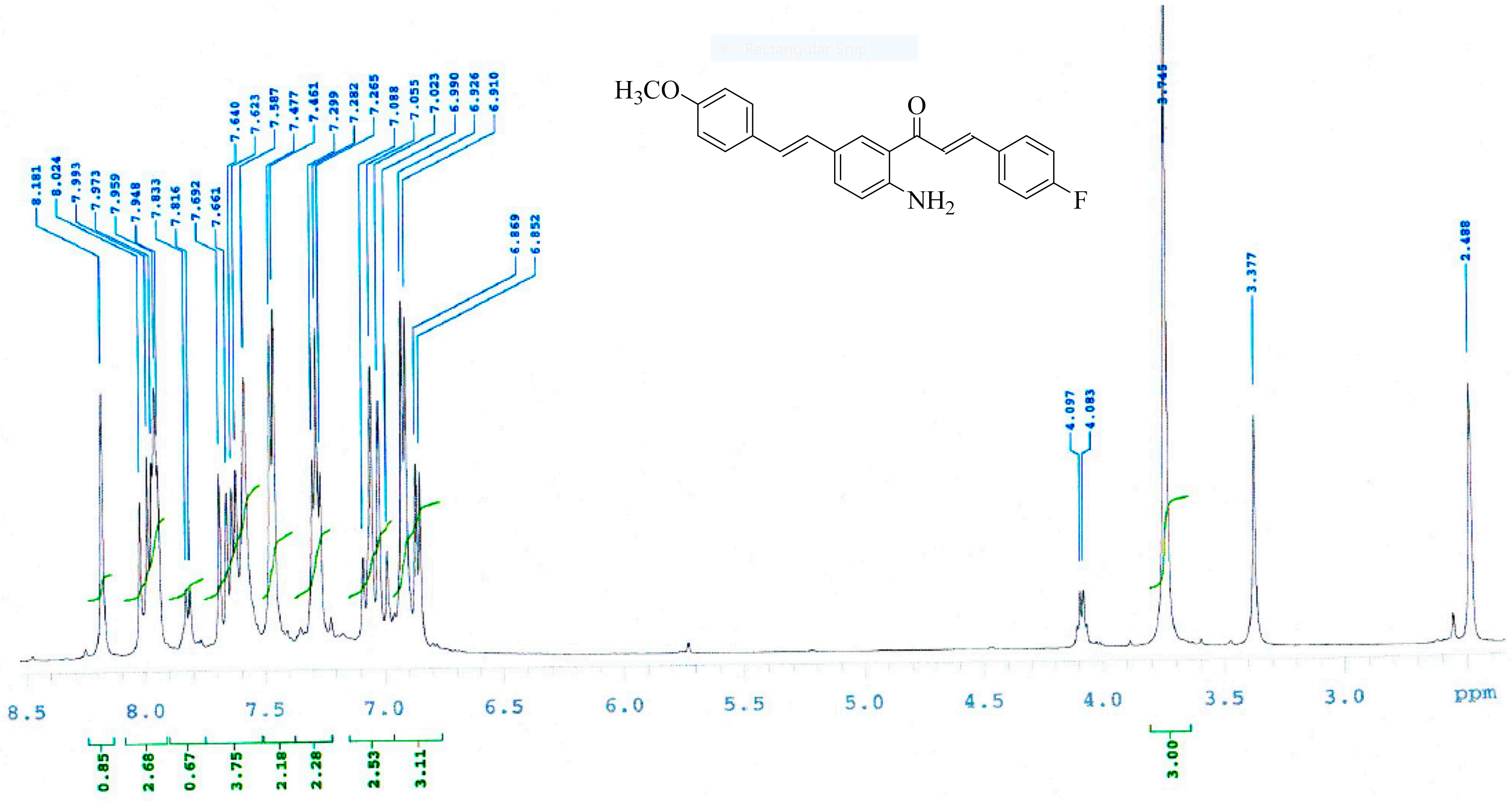
H3CO
O
NH2
F
8.181
8.024
7.993
7.973
7.959
7.948
7.833
7.816
7.692
7.661
7.640
7.623
7.587
7.477
7.461
7.299
7.282
7.265
7.088
7.055
7.023
6.990
6.926
6.910
6.869
6.852
4.097
4.083
3.745
3.377
2.488
8.5
8.0
7.5
7.0
6.5
6.0
5.5
5.0
4.5
4.0
3.5
3.0
ppm
0.85
2.68
0.67
3.75
2.18
2.28
2.53
3.11
3.00

(**a**)

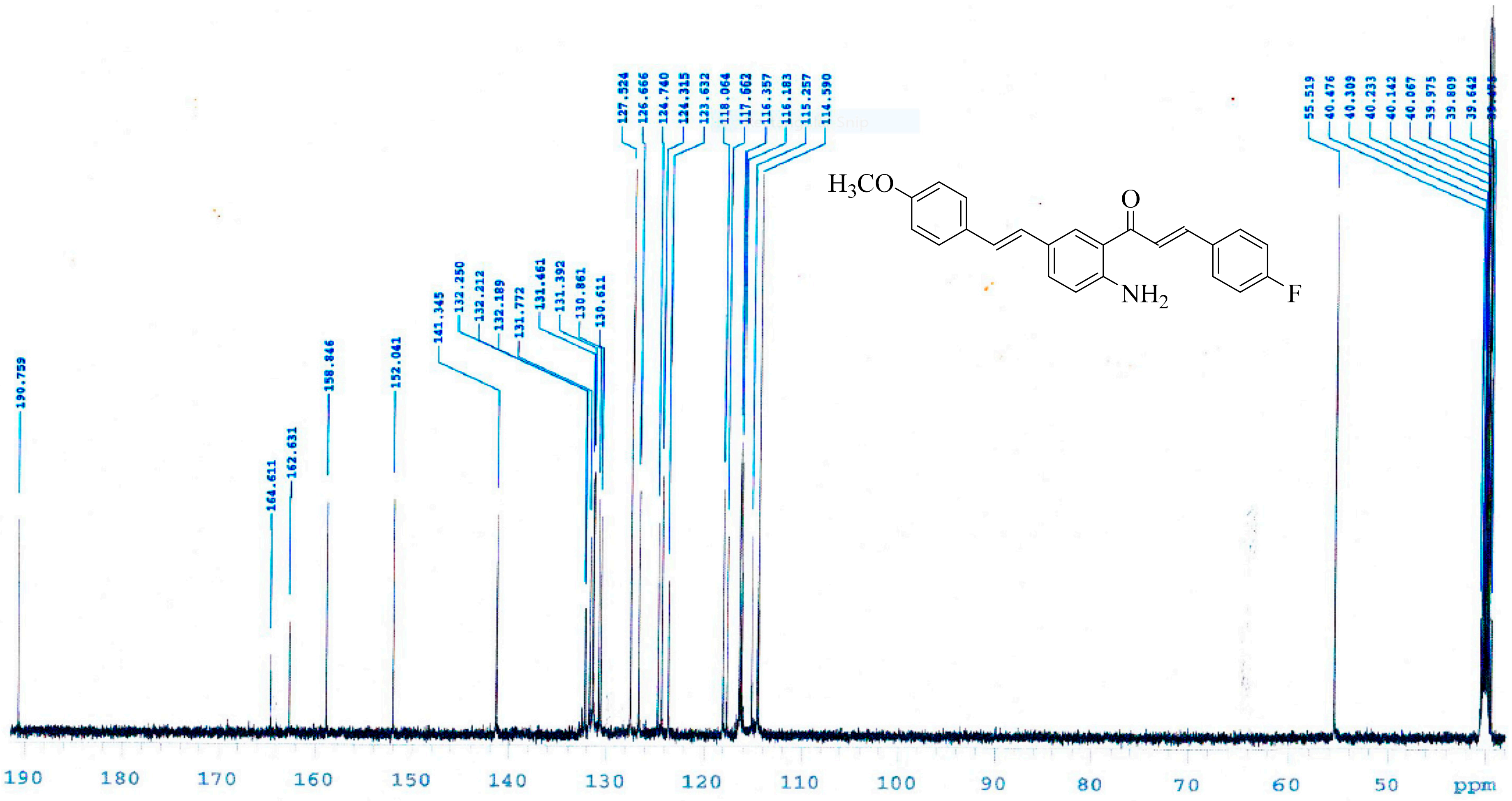

(**b**)

Figure S1.12: ^{1}H- and ^{13}C-NMR spectra of **3h** in DMSO-d_6 at 500 MHz (**a**) and 125 MHz (**b**), respectively.

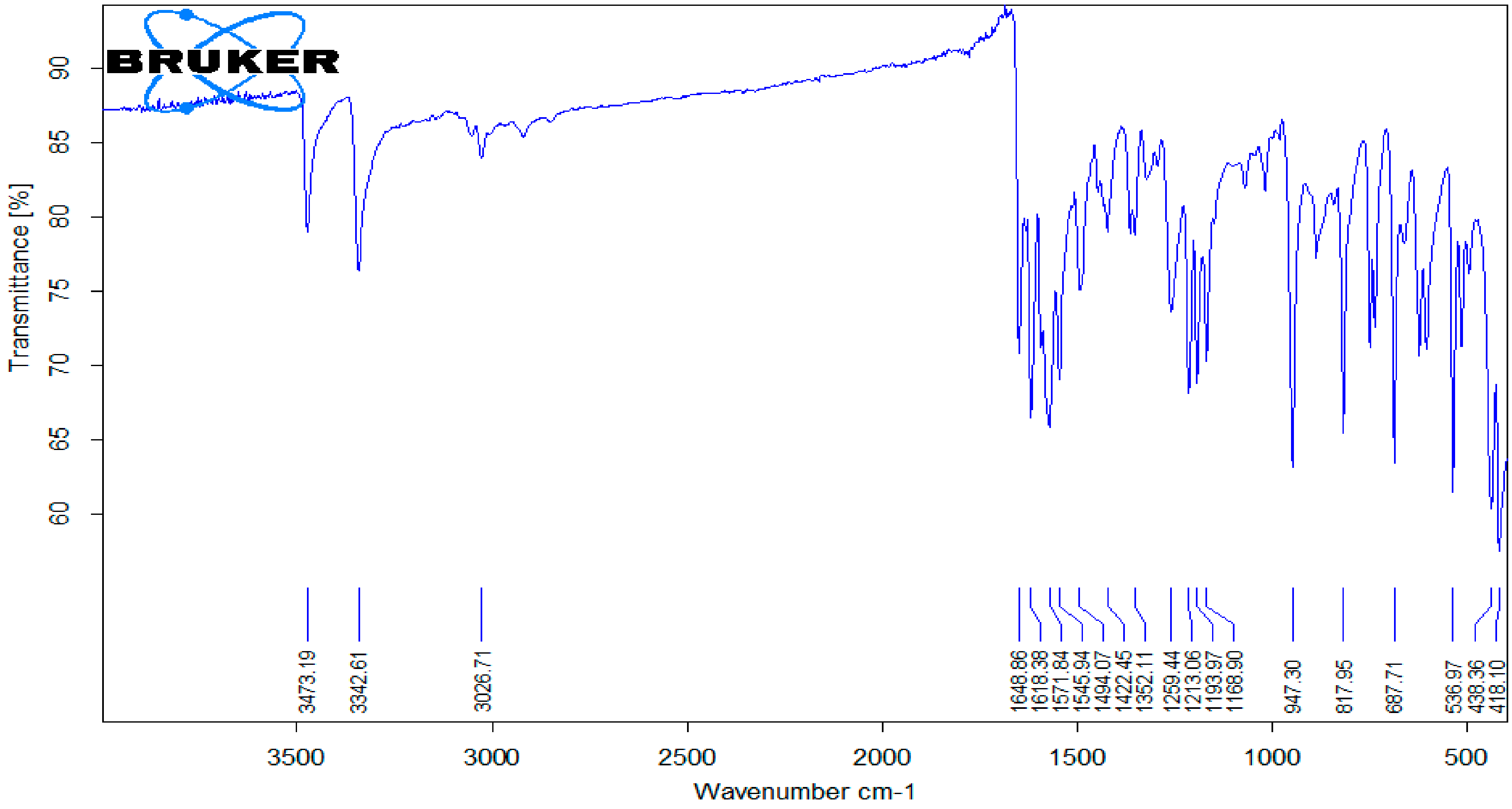

Figure S2.1: FT-IR spectrum of **2a**.

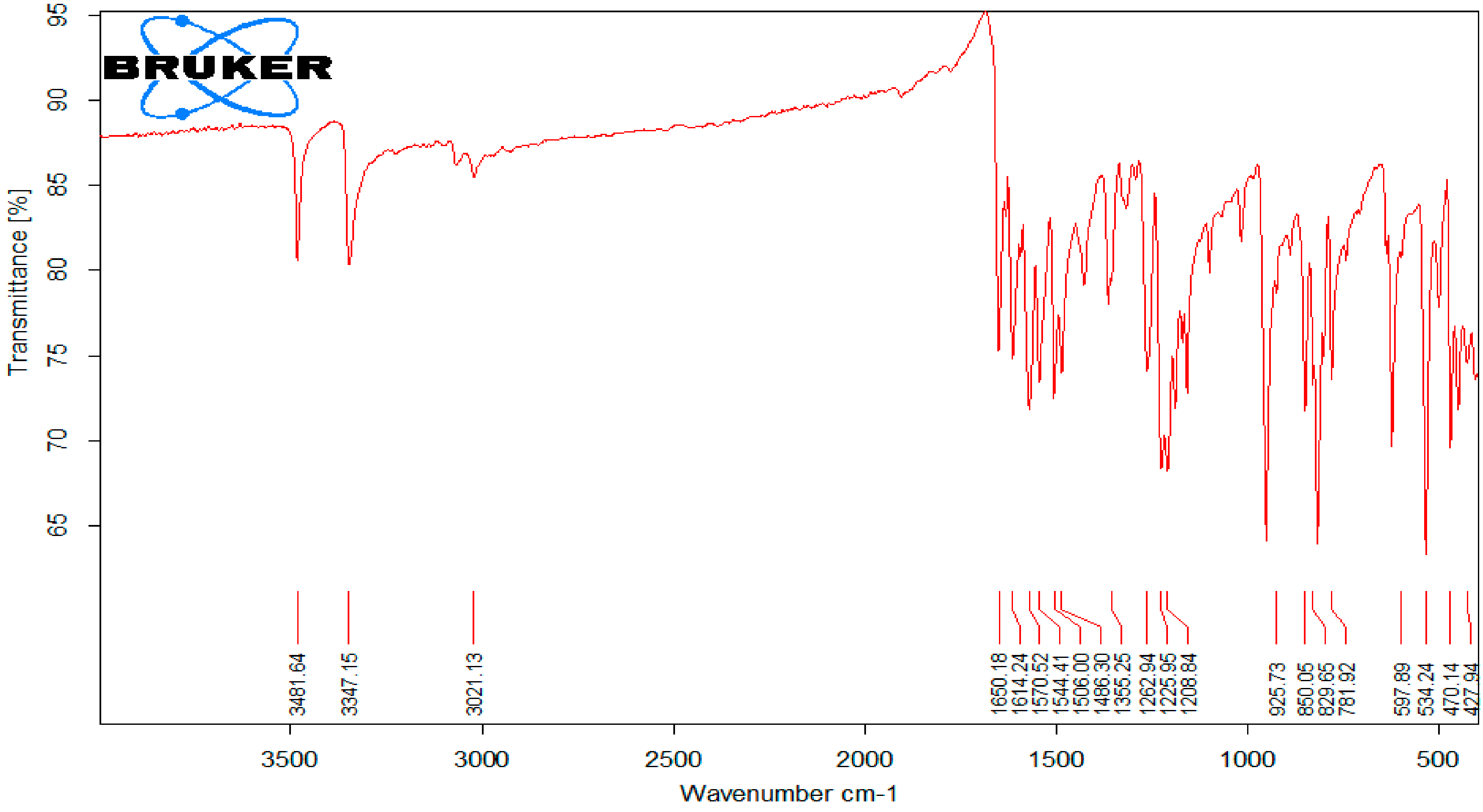

Figure S2.2: FT-IR spectrum of **2b**.

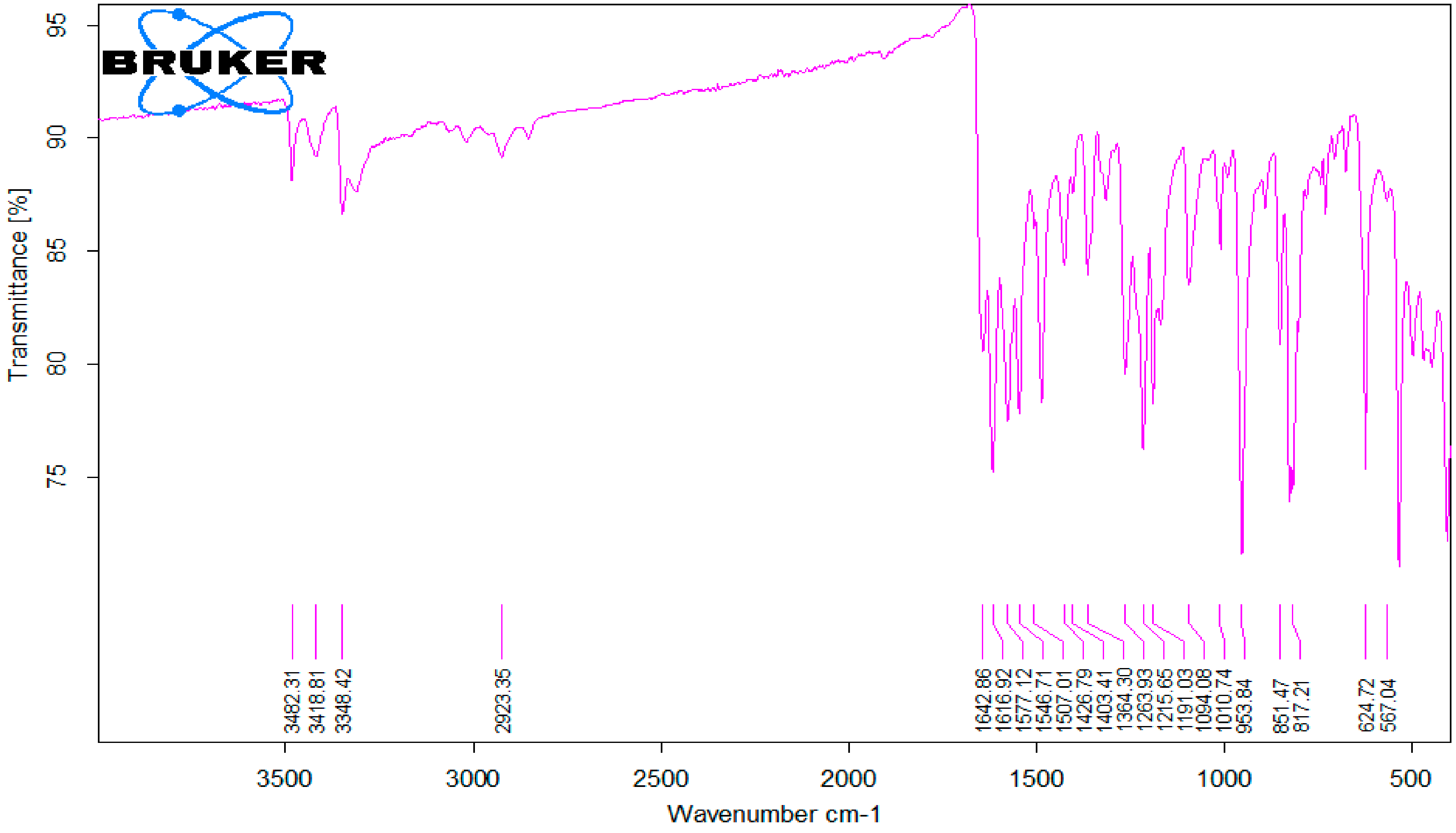

Figure S2.3: FT-IR spectrum of **2c**.

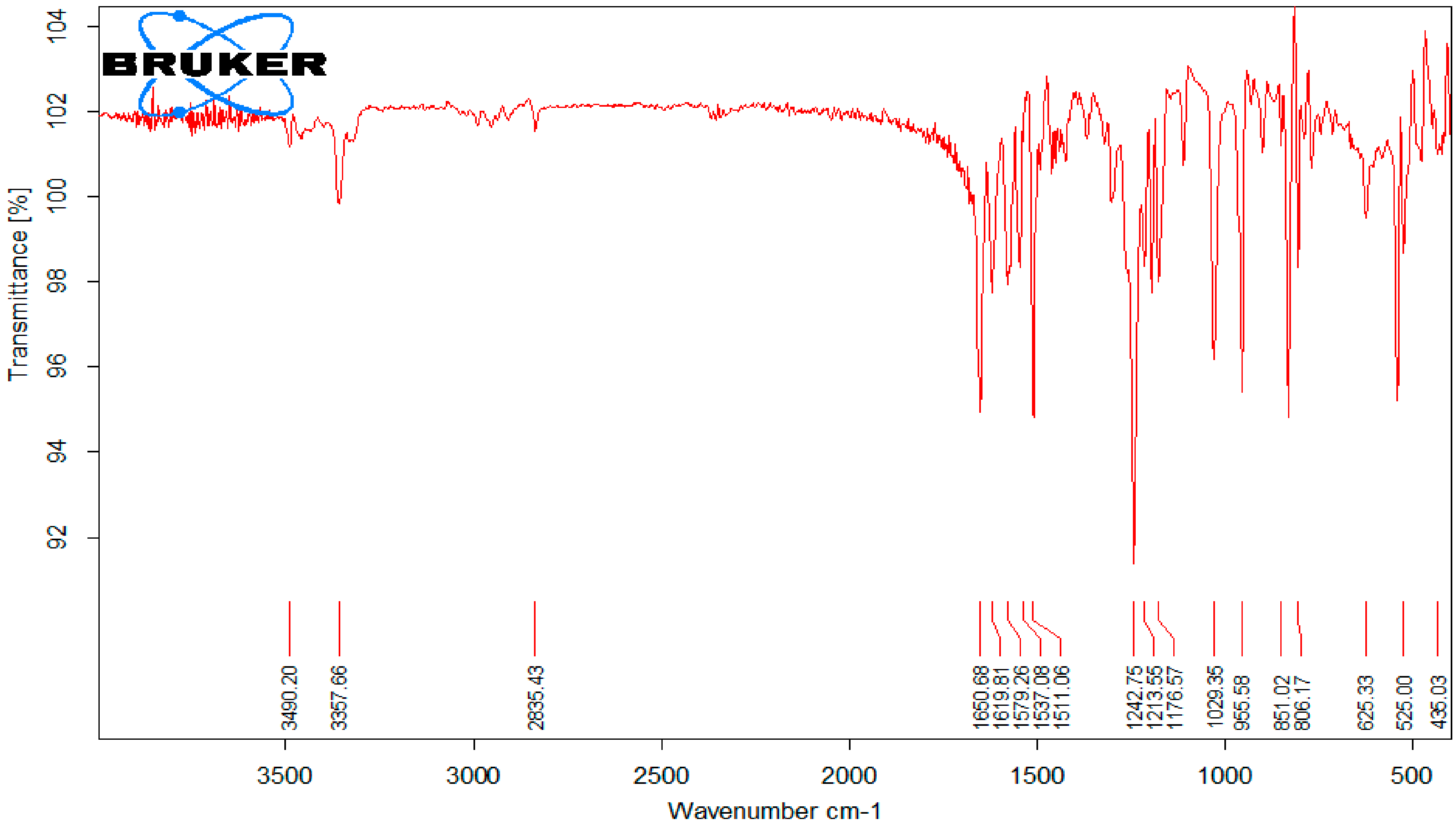

Figure S2.4: FT-IR spectrum of **2d**.

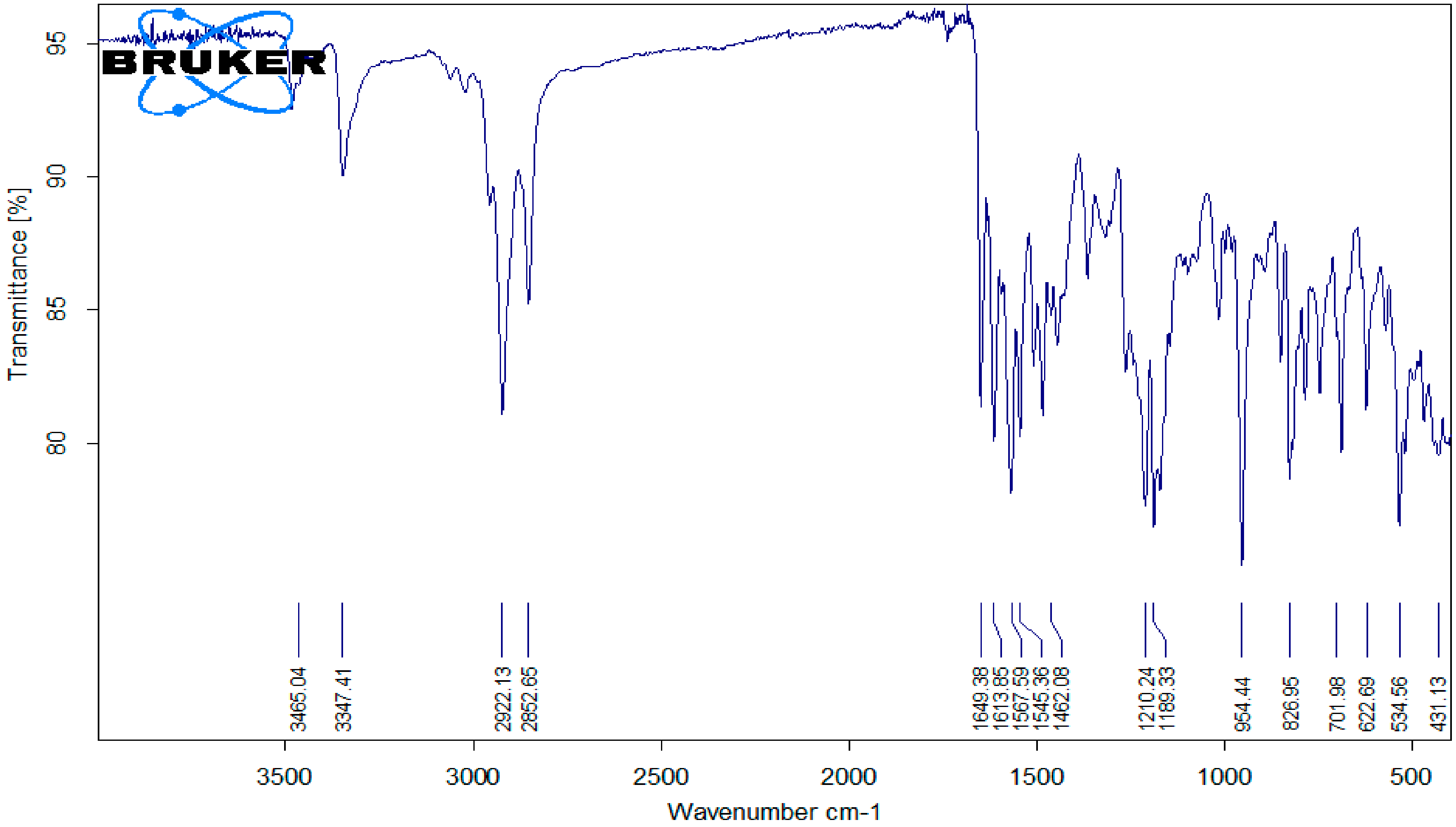

Figure S2.5: FT-IR spectrum of **3a**.

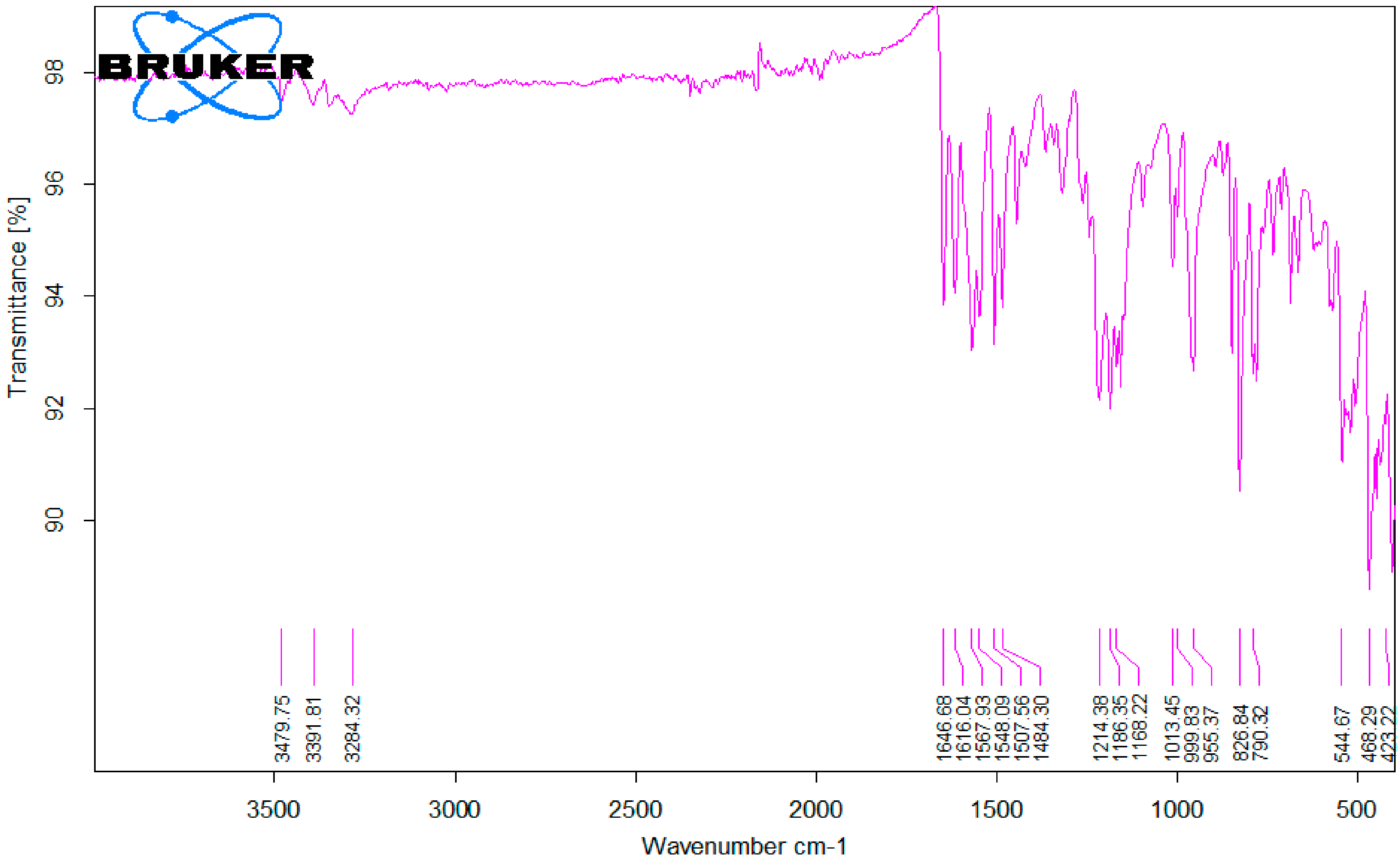

Figure S2.6: FT-IR spectrum of **3b**.

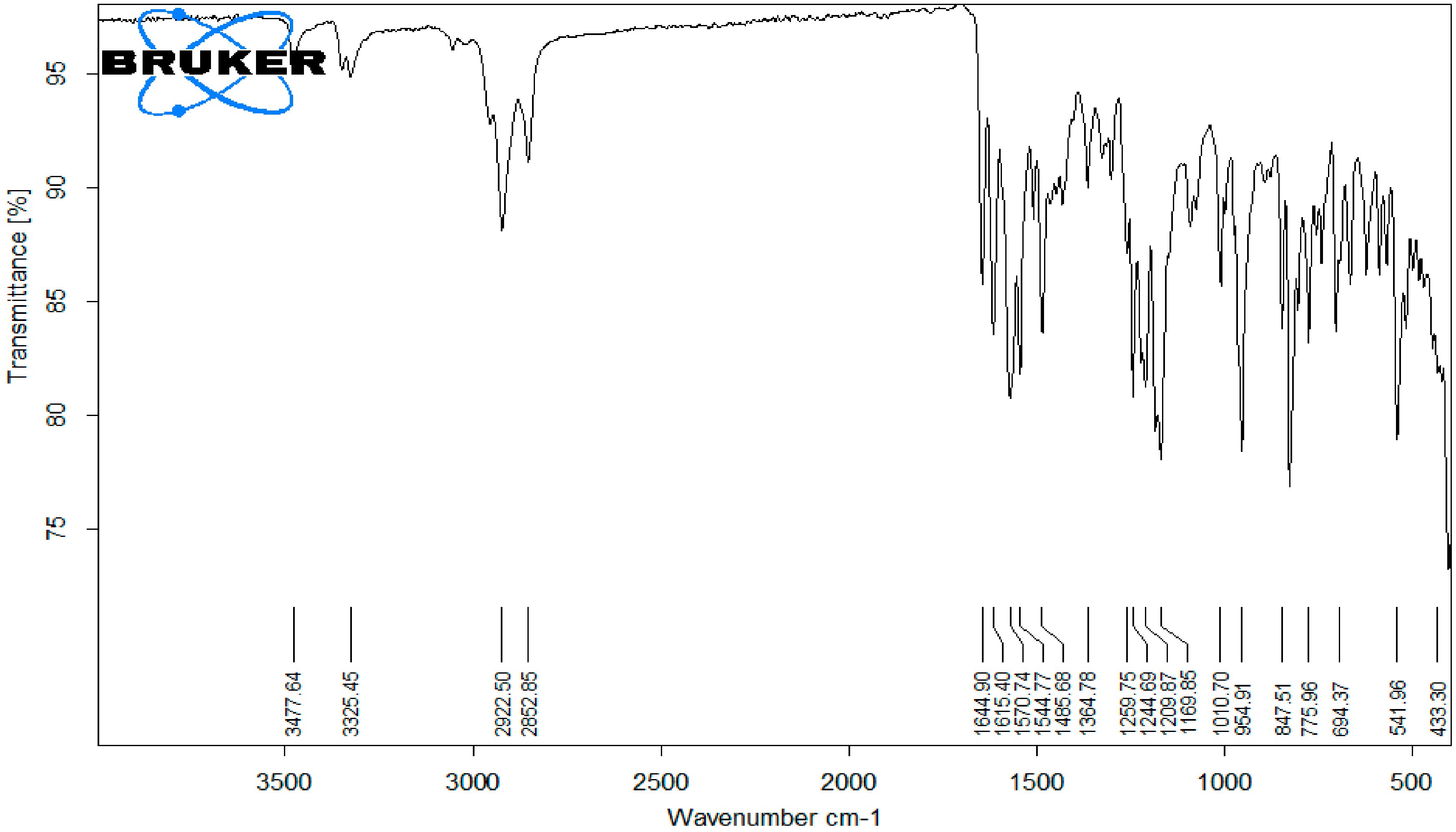

Figure S2.7: FT-IR spectrum of **3c**.

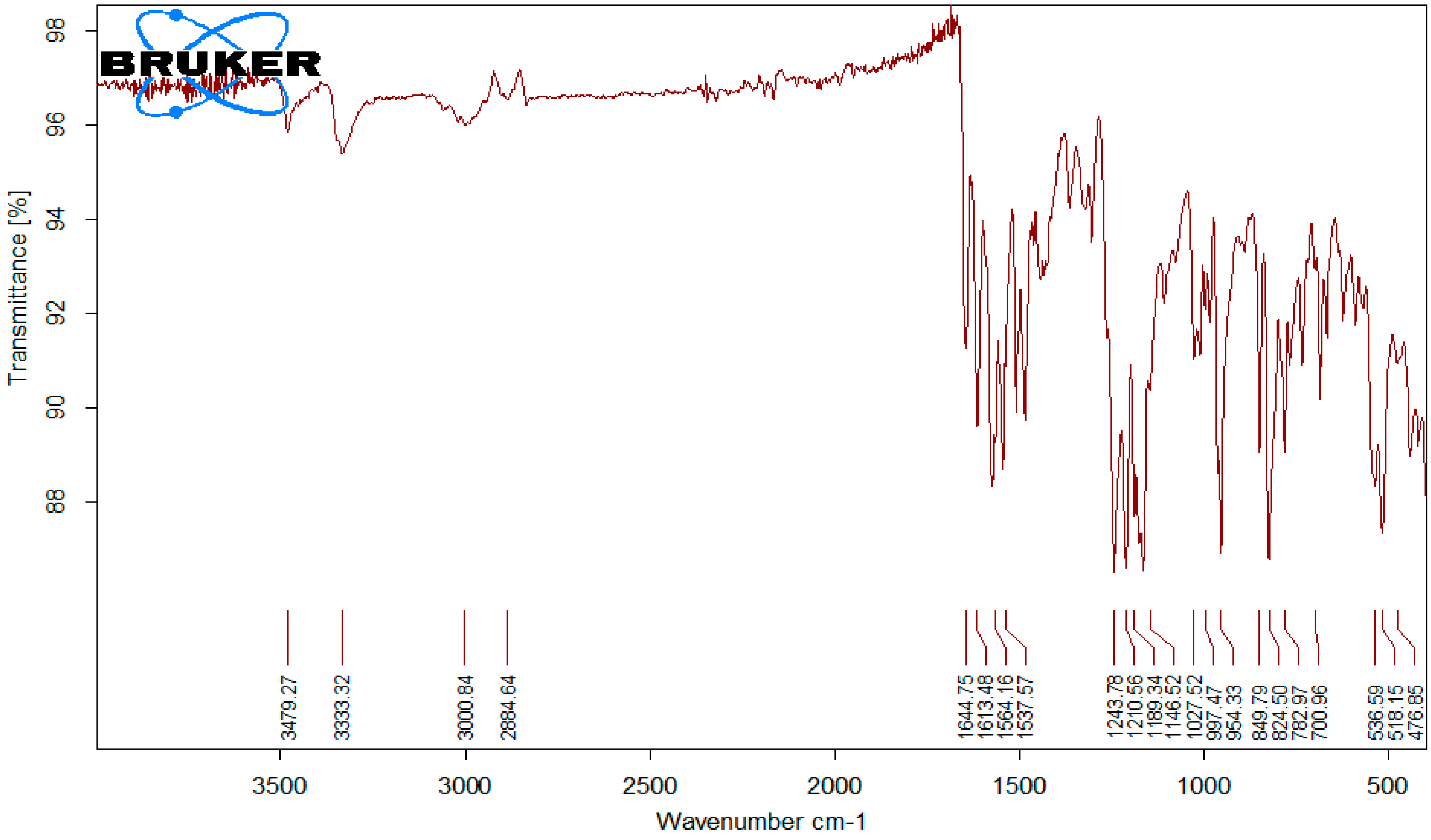

Figure S2.8: FT-IR spectrum of **3d**.

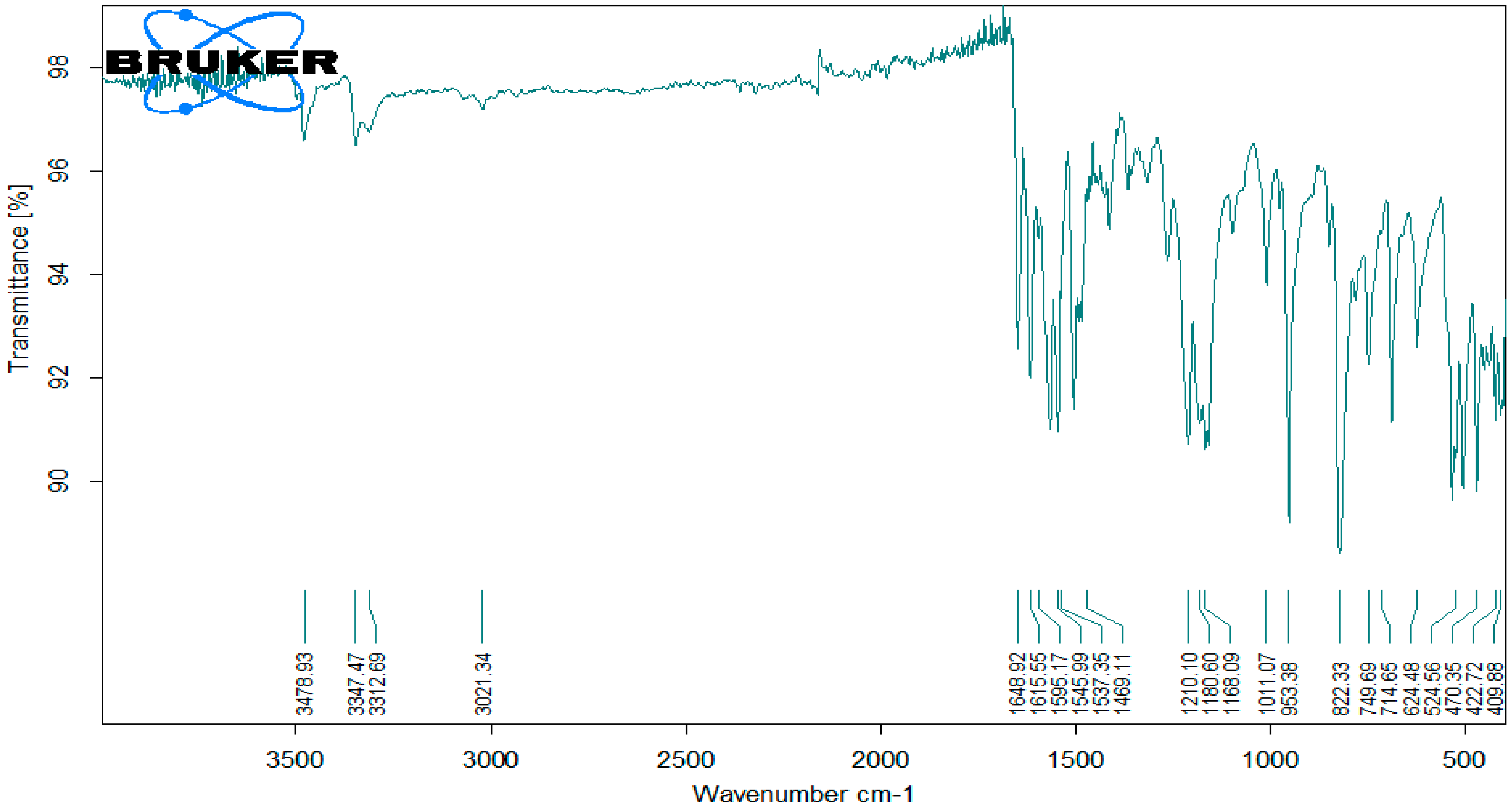

Figure S2.9: FT-IR spectrum of **3e**.

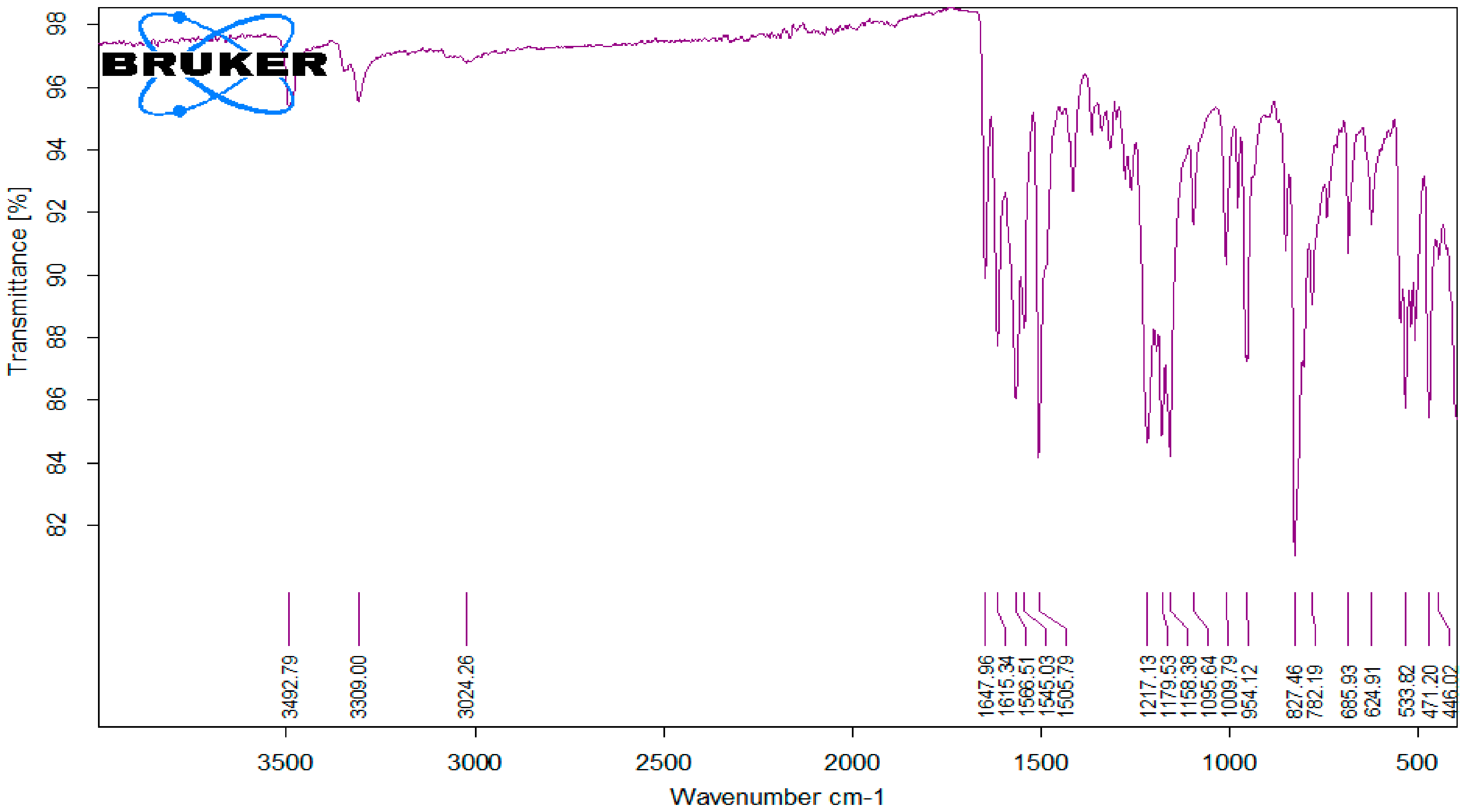

Figure S2.10: FT-IR spectrum of **3f**.

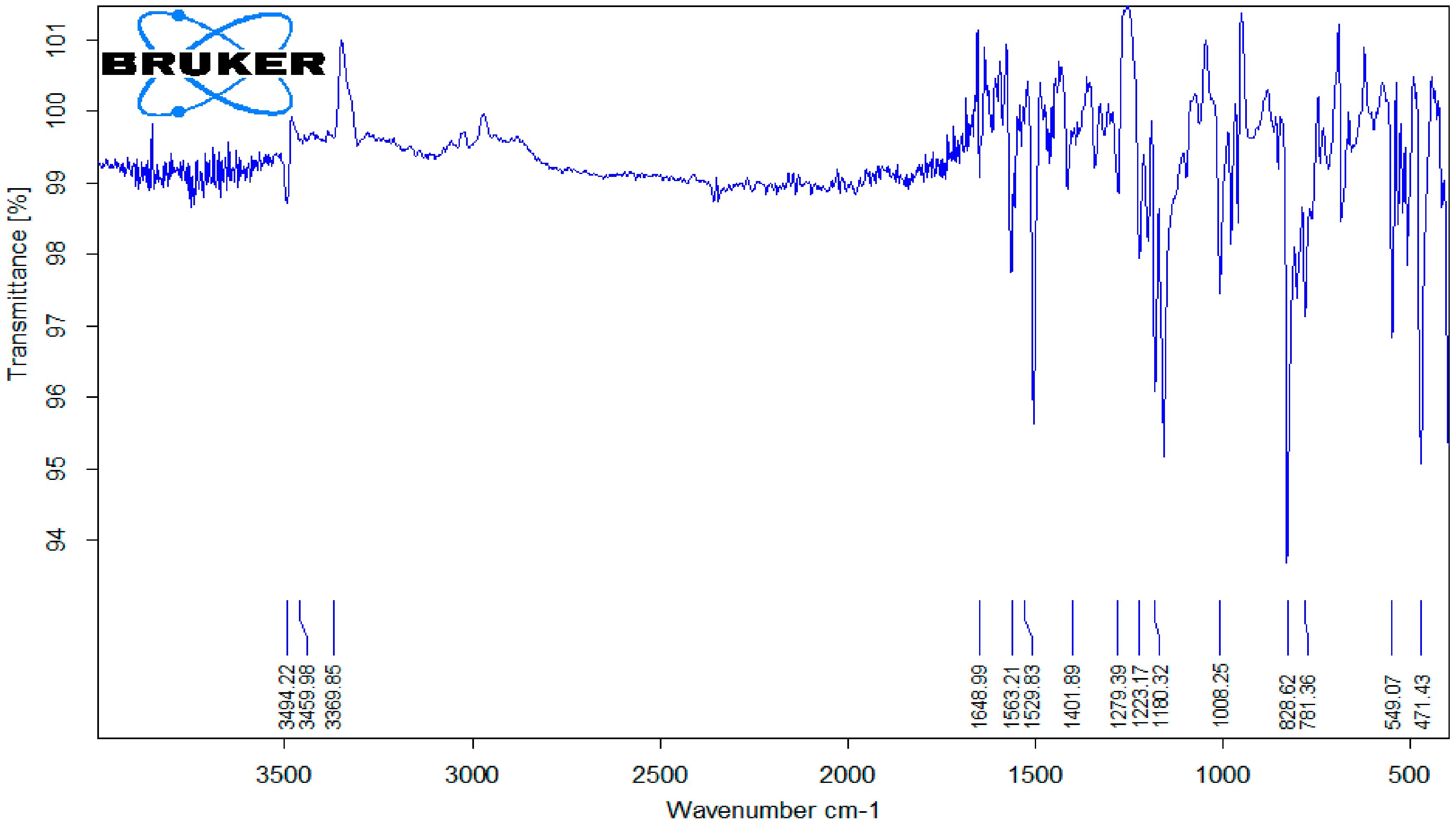

Figure S2.11: FT-IR spectrum of **3g**.

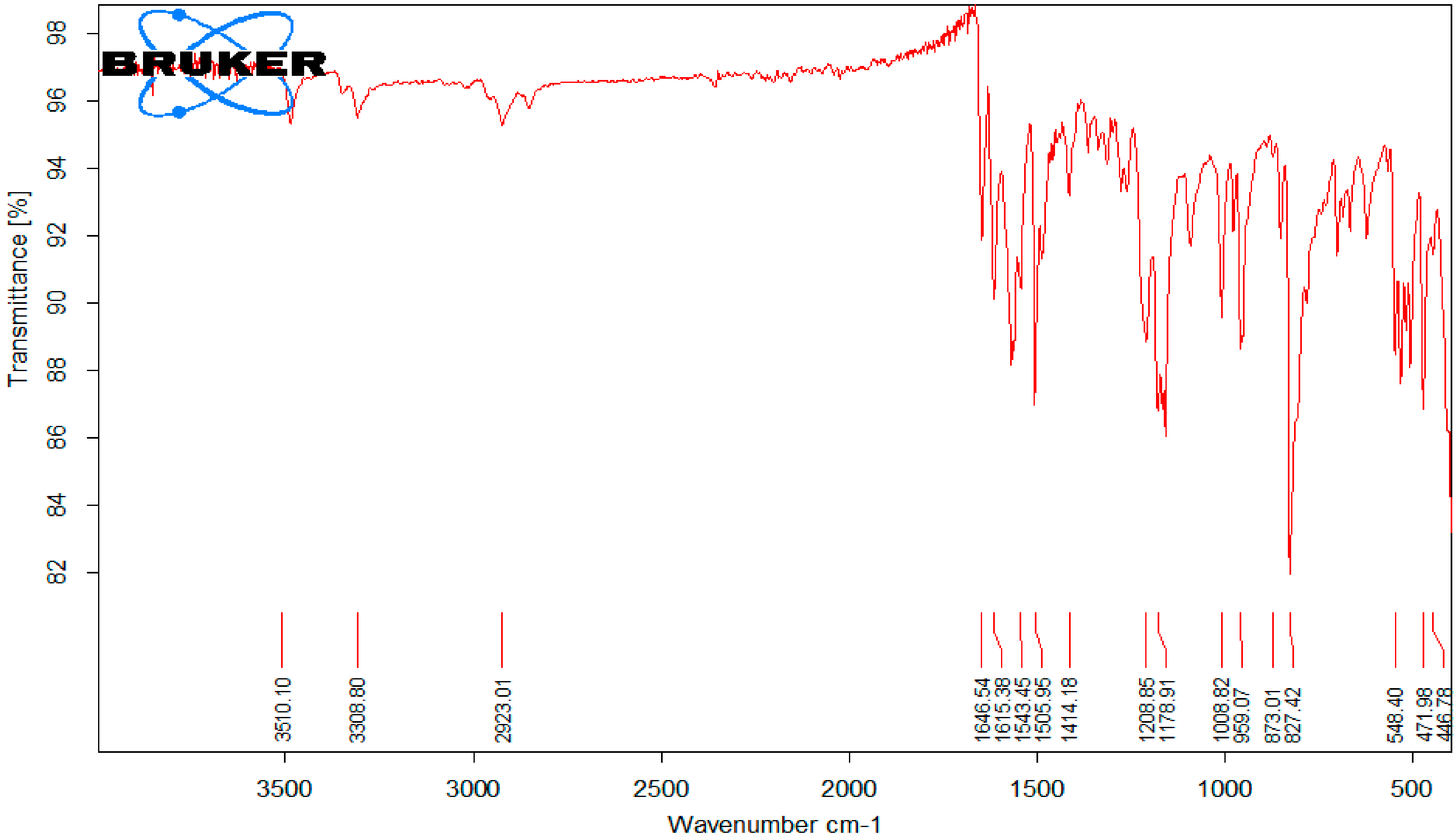

Figure S2.12: FT-IR spectrum of 3h.

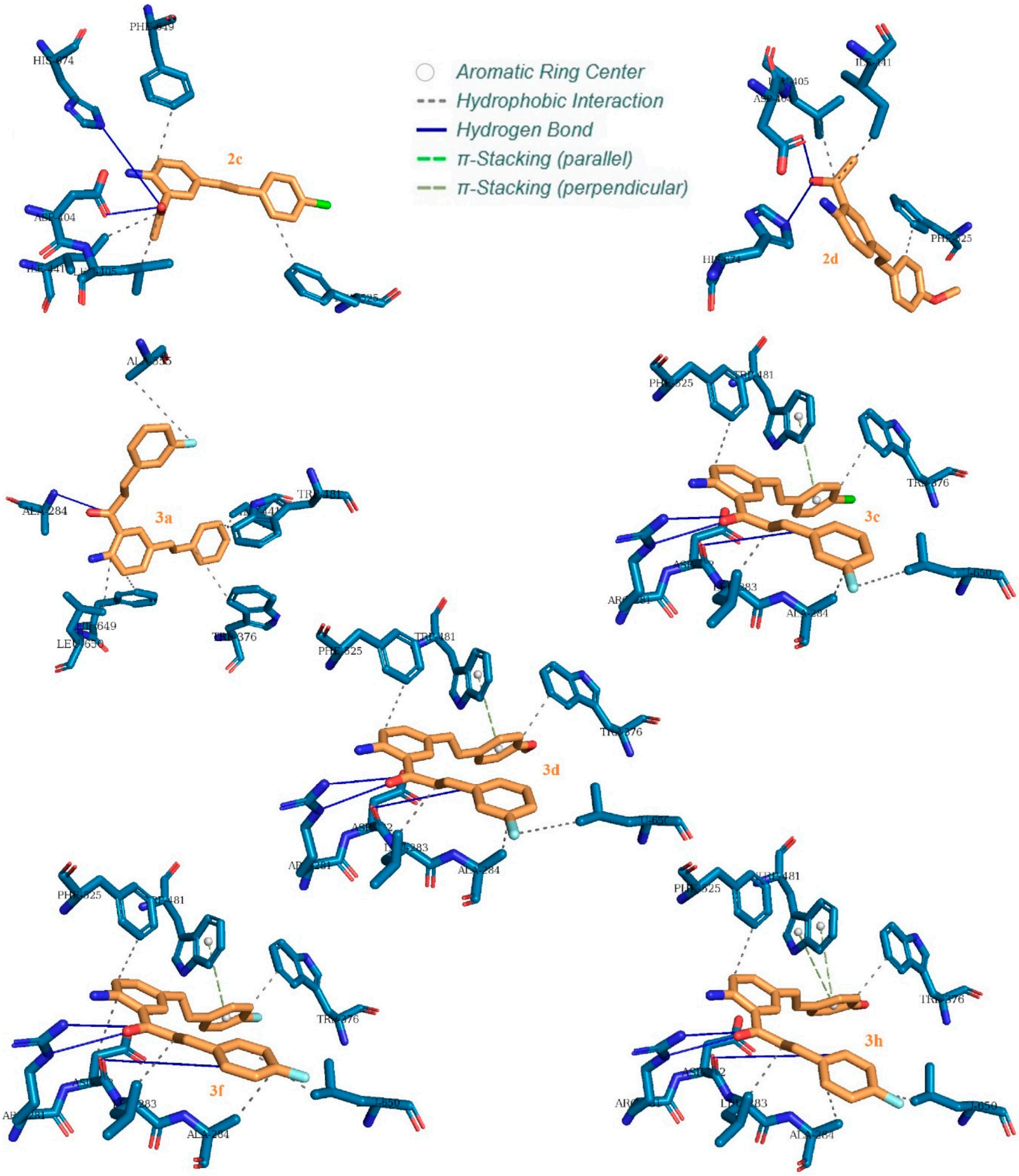

Figure S3. The interactions of compounds **2c, 2d**, **3a**, **3c**, **3d**, **3f** and **3h** against α-glucosidase.

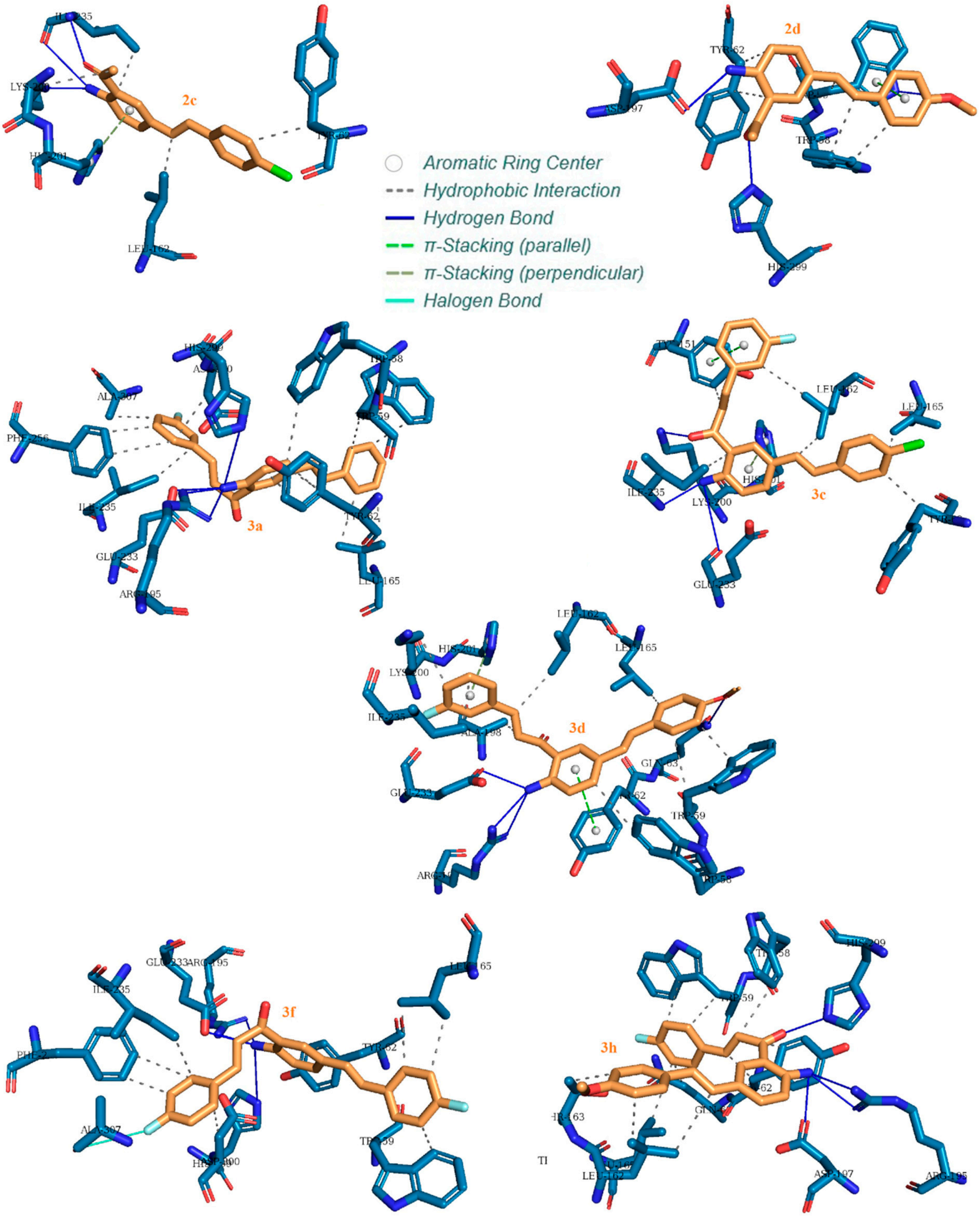

Figure S4. The interactions of compounds **2c, 2d**, **3a**, **3c**, **3d**, **3f** and **3h** against α-amylase.
